# Supplementary material for: Genetic and Pathogenic Characterization of Avian Influenza Virus in Migratory Birds between 2015 and 2019 in Central China
Source: Microbiol Spectr. 2022 Jul 12;10(4):e01652-22. doi: 10.1128/spectrum.01652-22 (PMC9431584; doi:10.1128/spectrum.01652-22)
Supplement: Supplemental file 3 — Fig. S1-4. Download spectrum.01652-22-s0001.pdf, PDF file, 3.1 MB [file spectrum.01652-22-s0001.pdf]

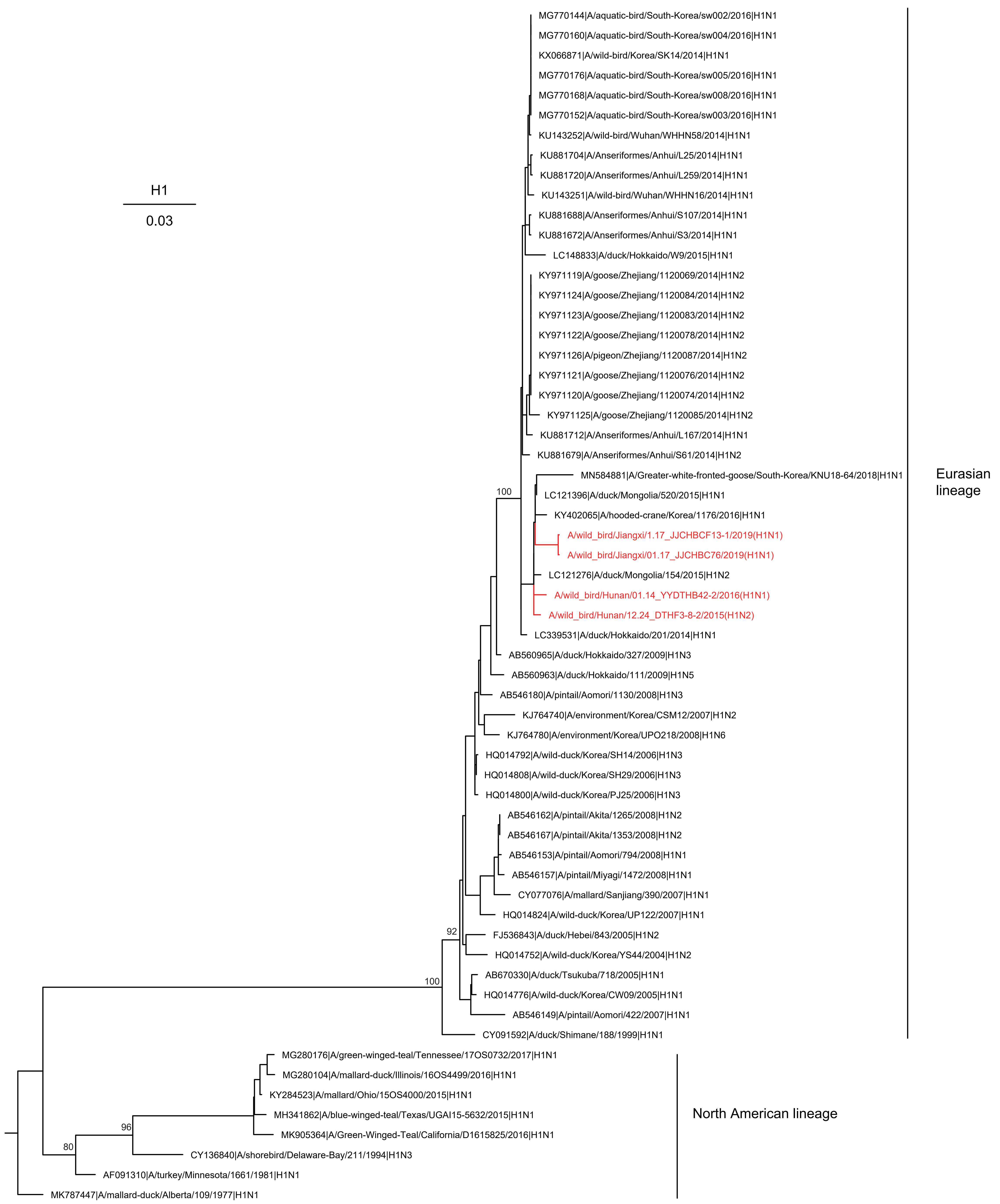

H1
0.03
MG770144|A/aquatic-bird/South-Korea/sw002/2016|H1N1
MG770160|A/aquatic-bird/South-Korea/sw004/2016|H1N1
KX066871|A/wild-bird/Korea/SK14/2014|H1N1
MG770176|A/aquatic-bird/South-Korea/sw005/2016|H1N1
MG770168|A/aquatic-bird/South-Korea/sw008/2016|H1N1
MG770152|A/aquatic-bird/South-Korea/sw003/2016|H1N1
KU143252|A/wild-bird/Wuhan/WHHN58/2014|H1N1
KU881704|A/Anseriformes/Anhui/L25/2014|H1N1
KU881720|A/Anseriformes/Anhui/L259/2014|H1N1
KU143251|A/wild-bird/Wuhan/WHHN16/2014|H1N1
KU881688|A/Anseriformes/Anhui/S107/2014|H1N1
KU881672|A/Anseriformes/Anhui/S3/2014|H1N1
LC148833|A/duck/Hokkaido/W9/2015|H1N1
KY971119|A/goose/Zhejiang/1120069/2014|H1N2
KY971124|A/goose/Zhejiang/1120084/2014|H1N2
KY971123|A/goose/Zhejiang/1120083/2014|H1N2
KY971122|A/goose/Zhejiang/1120078/2014|H1N2
KY971126|A/pigeon/Zhejiang/1120087/2014|H1N2
KY971121|A/goose/Zhejiang/1120076/2014|H1N2
KY971120|A/goose/Zhejiang/1120074/2014|H1N2
KY971125|A/goose/Zhejiang/1120085/2014|H1N2
KU881712|A/Anseriformes/Anhui/L167/2014|H1N1
KU881679|A/Anseriformes/Anhui/S61/2014|H1N2
MN584881|A/Greater-white-fronted-goose/South-Korea/KNU18-64/2018|H1N1
100
LC121396|A/duck/Mongolia/520/2015|H1N1
KY402065|A/hooded-crane/Korea/1176/2016|H1N1
A/wild_bird/Jiangxi/1.17_JJCHBCF13-1/2019(H1N1)
A/wild_bird/Jiangxi/01.17_JJCHBC76/2019(H1N1)
LC121276|A/duck/Mongolia/154/2015|H1N2
A/wild_bird/Hunan/01.14_YYDTHB42-2/2016(H1N1)
A/wild_bird/Hunan/12.24_DTHF3-8-2/2015(H1N2)
LC339531|A/duck/Hokkaido/201/2014|H1N1
AB560965|A/duck/Hokkaido/327/2009|H1N3
AB560963|A/duck/Hokkaido/111/2009|H1N5
AB546180|A/pintail/Aomori/1130/2008|H1N3
KJ764740|A/environment/Korea/CSM12/2007|H1N2
KJ764780|A/environment/Korea/UPO218/2008|H1N6
HQ014792|A/wild-duck/Korea/SH14/2006|H1N3
HQ014808|A/wild-duck/Korea/SH29/2006|H1N3
HQ014800|A/wild-duck/Korea/PJ25/2006|H1N3
AB546162|A/pintail/Akita/1265/2008|H1N2
AB546167|A/pintail/Akita/1353/2008|H1N2
AB546153|A/pintail/Aomori/794/2008|H1N1
AB546157|A/pintail/Miyagi/1472/2008|H1N1
CY077076|A/mallard/Sanjiang/390/2007|H1N1
HQ014824|A/wild-duck/Korea/UP122/2007|H1N1
92
FJ536843|A/duck/Hebei/843/2005|H1N2
HQ014752|A/wild-duck/Korea/YS44/2004|H1N2
100
AB670330|A/duck/Tsukuba/718/2005|H1N1
HQ014776|A/wild-duck/Korea/CW09/2005|H1N1
AB546149|A/pintail/Aomori/422/2007|H1N1
CY091592|A/duck/Shimane/188/1999|H1N1
Eurasian lineage
MG280176|A/green-winged-teal/Tennessee/17OS0732/2017|H1N1
MG280104|A/mallard-duck/Illinois/16OS4499/2016|H1N1
KY284523|A/mallard/Ohio/15OS4000/2015|H1N1
96
MH341862|A/blue-winged-teal/Texas/UGAI15-5632/2015|H1N1
MK905364|A/Green-Winged-Teal/California/D1615825/2016|H1N1
80
CY136840|A/shorebird/Delaware-Bay/211/1994|H1N3
AF091310|A/turkey/Minnesota/1661/1981|H1N1
MK787447|A/mallard-duck/Alberta/109/1977|H1N1
North American lineage

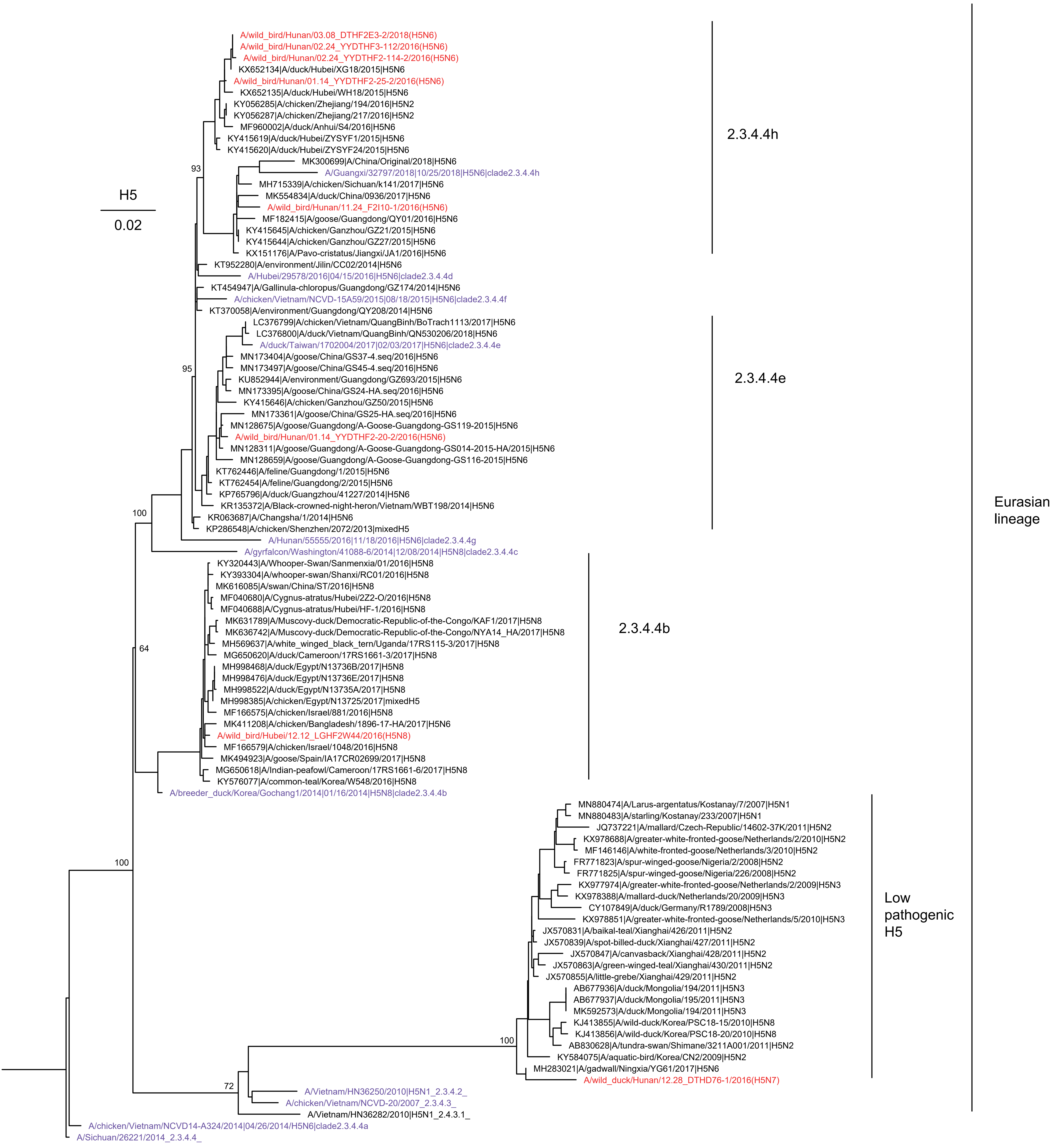
H5
0.02
A/wild_bird/Hunan/03.08_DTHF2E3-2/2018(H5N6)
A/wild_bird/Hunan/02.24_YYDTHF3-112/2016(H5N6)
A/wild_bird/Hunan/02.24_YYDTHF2-114-2/2016(H5N6)
KX652134|A/duck/Hubei/XG18/2015|H5N6
A/wild_bird/Hunan/01.14_YYDTHF2-25-2/2016(H5N6)
KX652135|A/duck/Hubei/WH18/2015|H5N6
KY056285|A/chicken/Zhejiang/194/2016|H5N2
KY056287|A/chicken/Zhejiang/217/2016|H5N2
MF960002|A/duck/Anhui/S4/2016|H5N6
KY415619|A/duck/Hubei/ZYSYF1/2015|H5N6
KY415620|A/duck/Hubei/ZYSYF24/2015|H5N6
93
MK300699|A/China/Original/2018|H5N6
A/Guangxi/32797/2018|10/25/2018|H5N6|clade2.3.4.4h
MH715339|A/chicken/Sichuan/k141/2017|H5N6
MK554834|A/duck/China/0936/2017|H5N6
A/wild_bird/Hunan/11.24_F2I10-1/2016(H5N6)
MF182415|A/goose/Guangdong/QY01/2016|H5N6
KY415645|A/chicken/Ganzhou/GZ21/2015|H5N6
KY415644|A/chicken/Ganzhou/GZ27/2015|H5N6
KX151176|A/Pavo-cristatus/Jiangxi/JA1/2016|H5N6
2.3.4.4h
KT952280|A/environment/Jilin/CC02/2014|H5N6
A/Hubei/29578/2016|04/15/2016|H5N6|clade2.3.4.4d
KT454947|A/Gallinula-chloropus/Guangdong/GZ174/2014|H5N6
A/chicken/Vietnam/NCVD-15A59/2015|08/18/2015|H5N6|clade2.3.4.4f
KT370058|A/environment/Guangdong/QY208/2014|H5N6
LC376799|A/chicken/Vietnam/QuangBinh/BoTrach1113/2017|H5N6
LC376800|A/duck/Vietnam/QuangBinh/QN530206/2018|H5N6
A/duck/Taiwan/1702004/2017|02/03/2017|H5N6|clade2.3.4.4e
MN173404|A/goose/China/GS37-4.seq/2016|H5N6
MN173497|A/goose/China/GS45-4.seq/2016|H5N6
95
KU852944|A/environment/Guangdong/GZ693/2015|H5N6
MN173395|A/goose/China/GS24-HA.seq/2016|H5N6
KY415646|A/chicken/Ganzhou/GZ50/2015|H5N6
MN173361|A/goose/China/GS25-HA.seq/2016|H5N6
MN128675|A/goose/Guangdong/A-Goose-Guangdong-GS119-2015|H5N6
A/wild_bird/Hunan/01.14_YYDTHF2-20-2/2016(H5N6)
MN128311|A/goose/Guangdong/A-Goose-Guangdong-GS014-2015-HA/2015|H5N6
MN128659|A/goose/Guangdong/A-Goose-Guangdong-GS116-2015|H5N6
KT762446|A/feline/Guangdong/1/2015|H5N6
KT762454|A/feline/Guangdong/2/2015|H5N6
KP765796|A/duck/Guangzhou/41227/2014|H5N6
KR135372|A/Black-crowned-night-heron/Vietnam/WBT198/2014|H5N6
100
KR063687|A/Changsha/1/2014|H5N6
KP286548|A/chicken/Shenzhen/2072/2013|mixedH5
2.3.4.4e
A/Hunan/55555/2016|11/18/2016|H5N6|clade2.3.4.4g
A/gyrfalcon/Washington/41088-6/2014|12/08/2014|H5N8|clade2.3.4.4c
KY320443|A/Whooper-Swan/Sanmenxia/01/2016|H5N8
KY393304|A/whooper-swan/Shanxi/RC01/2016|H5N8
MK616085|A/swan/China/ST/2016|H5N8
MF040680|A/Cygnus-atratus/Hubei/2Z2-O/2016|H5N8
MF040688|A/Cygnus-atratus/Hubei/HF-1/2016|H5N8
MK631789|A/Muscovy-duck/Democratic-Republic-of-the-Congo/KAF1/2017|H5N8
MK636742|A/Muscovy-duck/Democratic-Republic-of-the-Congo/NYA14_HA/2017|H5N8
64
MH569637|A/white_winged_black_tern/Uganda/17RS115-3/2017|H5N8
MG650620|A/duck/Cameroon/17RS1661-3/2017|H5N8
MH998468|A/duck/Egypt/N13736B/2017|H5N8
MH998476|A/duck/Egypt/N13736E/2017|H5N8
MH998522|A/duck/Egypt/N13735A/2017|H5N8
MH998385|A/chicken/Egypt/N13725/2017|mixedH5
MF166575|A/chicken/Israel/881/2016|H5N8
MK411208|A/chicken/Bangladesh/1896-17-HA/2017|H5N6
A/wild_bird/Hubei/12.12_LGHF2W44/2016(H5N8)
MF166579|A/chicken/Israel/1048/2016|H5N8
MK494923|A/goose/Spain/IA17CR02699/2017|H5N8
MG650618|A/Indian-peafowl/Cameroon/17RS1661-6/2017|H5N8
KY576077|A/common-teal/Korea/W548/2016|H5N8
2.3.4.4b
A/breeder_duck/Korea/Gochang1/2014|01/16/2014|H5N8|clade2.3.4.4b
MN880474|A/Larus-argentatus/Kostanay/7/2007|H5N1
MN880483|A/starling/Kostanay/233/2007|H5N1
JQ737221|A/mallard/Czech-Republic/14602-37K/2011|H5N2
KX978688|A/greater-white-fronted-goose/Netherlands/2/2010|H5N2
MF146146|A/white-fronted-goose/Netherlands/3/2010|H5N2
100
FR771823|A/spur-winged-goose/Nigeria/2/2008|H5N2
FR771825|A/spur-winged-goose/Nigeria/226/2008|H5N2
KX977974|A/greater-white-fronted-goose/Netherlands/2/2009|H5N3
KX978388|A/mallard-duck/Netherlands/20/2009|H5N3
CY107849|A/duck/Germany/R1789/2008|H5N3
KX978851|A/greater-white-fronted-goose/Netherlands/5/2010|H5N3
JX570831|A/baikal-teal/Xianghai/426/2011|H5N2
JX570839|A/spot-billed-duck/Xianghai/427/2011|H5N2
JX570847|A/canvasback/Xianghai/428/2011|H5N2
JX570863|A/green-winged-teal/Xianghai/430/2011|H5N2
JX570855|A/little-grebe/Xianghai/429/2011|H5N2
AB677936|A/duck/Mongolia/194/2011|H5N3
AB677937|A/duck/Mongolia/195/2011|H5N3
MK592573|A/duck/Mongolia/194/2011|H5N3
KJ413855|A/wild-duck/Korea/PSC18-15/2010|H5N8
KJ413856|A/wild-duck/Korea/PSC18-20/2010|H5N8
100
AB830628|A/tundra-swan/Shimane/3211A001/2011|H5N2
KY584075|A/aquatic-bird/Korea/CN2/2009|H5N2
MH283021|A/gadwall/Ningxia/YG61/2017|H5N6
A/wild_duck/Hunan/12.28_DTHD76-1/2016(H5N7)
Low pathogenic H5
72
A/Vietnam/HN36250/2010|H5N1_2.3.4.2_
A/chicken/Vietnam/NCVD-20/2007_2.3.4.3_
A/Vietnam/HN36282/2010|H5N1_2.4.3.1_
A/chicken/Vietnam/NCVD14-A324/2014|04/26/2014|H5N6|clade2.3.4.4a
A/Sichuan/26221/2014_2.3.4.4_
Eurasian lineage

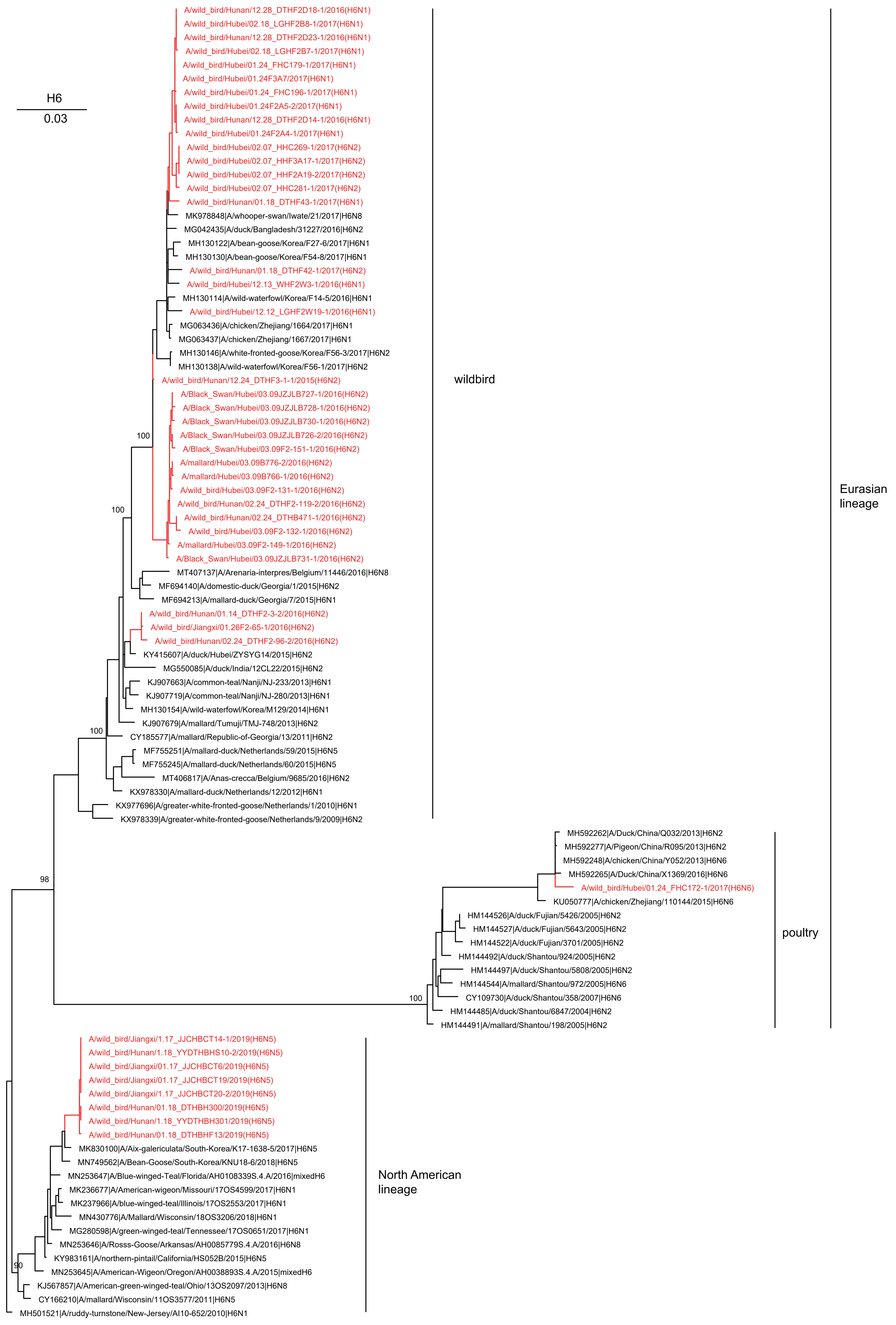

H6
0.03
A/wild_bird/Hunan/12.28_DTHF2D18-1/2016(H6N1)
A/wild_bird/Hubei/02.18_LGHF2B8-1/2017(H6N1)
A/wild_bird/Hunan/12.28_DTHF2D23-1/2016(H6N1)
A/wild_bird/Hubei/02.18_LGHF2B7-1/2017(H6N1)
A/wild_bird/Hubei/01.24_FHC179-1/2017(H6N1)
A/wild_bird/Hubei/01.24F3A7/2017(H6N1)
A/wild_bird/Hubei/01.24_FHC196-1/2017(H6N1)
A/wild_bird/Hubei/01.24F2A5-2/2017(H6N1)
A/wild_bird/Hunan/12.28_DTHF2D14-1/2016(H6N1)
A/wild_bird/Hubei/01.24F2A4-1/2017(H6N1)
A/wild_bird/Hubei/02.07_HHC269-1/2017(H6N2)
A/wild_bird/Hubei/02.07_HHF3A17-1/2017(H6N2)
A/wild_bird/Hubei/02.07_HHF2A19-2/2017(H6N2)
A/wild_bird/Hubei/02.07_HHC281-1/2017(H6N2)
A/wild_bird/Hunan/01.18_DTHF43-1/2017(H6N1)
MK978848|A/whooper-swan/Iwate/21/2017|H6N8
MG042435|A/duck/Bangladesh/31227/2016|H6N2
MH130122|A/bean-goose/Korea/F27-6/2017|H6N1
MH130130|A/bean-goose/Korea/F54-8/2017|H6N1
A/wild_bird/Hunan/01.18_DTHF42-1/2017(H6N2)
A/wild_bird/Hubei/12.13_WHF2W3-1/2016(H6N1)
MH130114|A/wild-waterfowl/Korea/F14-5/2016|H6N1
A/wild_bird/Hubei/12.12_LGHF2W19-1/2016(H6N1)
MG063436|A/chicken/Zhejiang/1664/2017|H6N1
MG063437|A/chicken/Zhejiang/1667/2017|H6N1
MH130146|A/white-fronted-goose/Korea/F56-3/2017|H6N2
MH130138|A/wild-waterfowl/Korea/F56-1/2017|H6N2
A/wild_bird/Hunan/12.24_DTHF3-1-1/2015(H6N2)
A/Black_Swan/Hubei/03.09JZJLB727-1/2016(H6N2)
A/Black_Swan/Hubei/03.09JZJLB728-1/2016(H6N2)
A/Black_Swan/Hubei/03.09JZJLB730-1/2016(H6N2)
A/Black_Swan/Hubei/03.09JZJLB726-2/2016(H6N2)
A/Black_Swan/Hubei/03.09F2-151-1/2016(H6N2)
A/mallard/Hubei/03.09B776-2/2016(H6N2)
A/mallard/Hubei/03.09B766-1/2016(H6N2)
A/wild_bird/Hubei/03.09F2-131-1/2016(H6N2)
A/wild_bird/Hunan/02.24_DTHF2-119-2/2016(H6N2)
A/wild_bird/Hunan/02.24_DTHB471-1/2016(H6N2)
A/wild_bird/Hubei/03.09F2-132-1/2016(H6N2)
A/mallard/Hubei/03.09F2-149-1/2016(H6N2)
A/Black_Swan/Hubei/03.09JZJLB731-1/2016(H6N2)
MT407137|A/Arenaria-interpres/Belgium/11446/2016|H6N8
MF694140|A/domestic-duck/Georgia/1/2015|H6N2
MF694213|A/mallard-duck/Georgia/7/2015|H6N1
A/wild_bird/Hunan/01.14_DTHF2-3-2/2016(H6N2)
A/wild_bird/Jiangxi/01.26F2-65-1/2016(H6N2)
A/wild_bird/Hunan/02.24_DTHF2-96-2/2016(H6N2)
KY415607|A/duck/Hubei/ZYSYG14/2015|H6N2
MG550085|A/duck/India/12CL22/2015|H6N2
KJ907663|A/common-teal/Nanji/NJ-233/2013|H6N1
KJ907719|A/common-teal/Nanji/NJ-280/2013|H6N1
MH130154|A/wild-waterfowl/Korea/M129/2014|H6N1
KJ907679|A/mallard/Tumuji/TMJ-748/2013|H6N2
CY185577|A/mallard/Republic-of-Georgia/13/2011|H6N2
MF755251|A/mallard-duck/Netherlands/59/2015|H6N5
MF755245|A/mallard-duck/Netherlands/60/2015|H6N5
MT406817|A/Anas-crecca/Belgium/9685/2016|H6N2
KX978330|A/mallard-duck/Netherlands/12/2012|H6N1
KX977696|A/greater-white-fronted-goose/Netherlands/1/2010|H6N1
KX978339|A/greater-white-fronted-goose/Netherlands/9/2009|H6N2
100
100
100
98
wildbird
Eurasian lineage
MH592262|A/Duck/China/Q032/2013|H6N2
MH592277|A/Pigeon/China/R095/2013|H6N2
MH592248|A/chicken/China/Y052/2013|H6N6
MH592265|A/Duck/China/X1369/2016|H6N6
A/wild_bird/Hubei/01.24_FHC172-1/2017(H6N6)
KU050777|A/chicken/Zhejiang/110144/2015|H6N6
HM144526|A/duck/Fujian/5426/2005|H6N2
HM144527|A/duck/Fujian/5643/2005|H6N2
HM144522|A/duck/Fujian/3701/2005|H6N2
HM144492|A/duck/Shantou/924/2005|H6N2
HM144497|A/duck/Shantou/5808/2005|H6N2
HM144544|A/mallard/Shantou/972/2005|H6N6
CY109730|A/duck/Shantou/358/2007|H6N6
HM144485|A/duck/Shantou/6847/2004|H6N2
HM144491|A/mallard/Shantou/198/2005|H6N2
100
poultry
A/wild_bird/Jiangxi/1.17_JJCHBCT14-1/2019(H6N5)
A/wild_bird/Hunan/1.18_YYDTHBHS10-2/2019(H6N5)
A/wild_bird/Jiangxi/01.17_JJCHBCT6/2019(H6N5)
A/wild_bird/Jiangxi/01.17_JJCHBCT19/2019(H6N5)
A/wild_bird/Jiangxi/1.17_JJCHBCT20-2/2019(H6N5)
A/wild_bird/Hunan/01.18_DTHBH300/2019(H6N5)
A/wild_bird/Hunan/1.18_YYDTHBH301/2019(H6N5)
A/wild_bird/Hunan/01.18_DTHBHF13/2019(H6N5)
MK830100|A/Aix-galericulata/South-Korea/K17-1638-5/2017|H6N5
MN749562|A/Bean-Goose/South-Korea/KNU18-6/2018|H6N5
MN253647|A/Blue-winged-Teal/Florida/AH0108339S.4.A/2016|mixedH6
MK236677|A/American-wigeon/Missouri/17OS4599/2017|H6N1
MK237966|A/blue-winged-teal/Illinois/17OS2553/2017|H6N1
MN430776|A/Mallard/Wisconsin/18OS3206/2018|H6N1
MG280598|A/green-winged-teal/Tennessee/17OS0651/2017|H6N1
MN253646|A/Rosss-Goose/Arkansas/AH0085779S.4.A/2016|H6N8
KY983161|A/northern-pintail/California/HS052B/2015|H6N5
MN253645|A/American-Wigeon/Oregon/AH0038893S.4.A/2015|mixedH6
KJ567857|A/American-green-winged-teal/Ohio/13OS2097/2013|H6N8
CY166210|A/mallard/Wisconsin/11OS3577/2011|H6N5
MH501521|A/ruddy-turnstone/New-Jersey/AI10-652/2010|H6N1
90
North American lineage

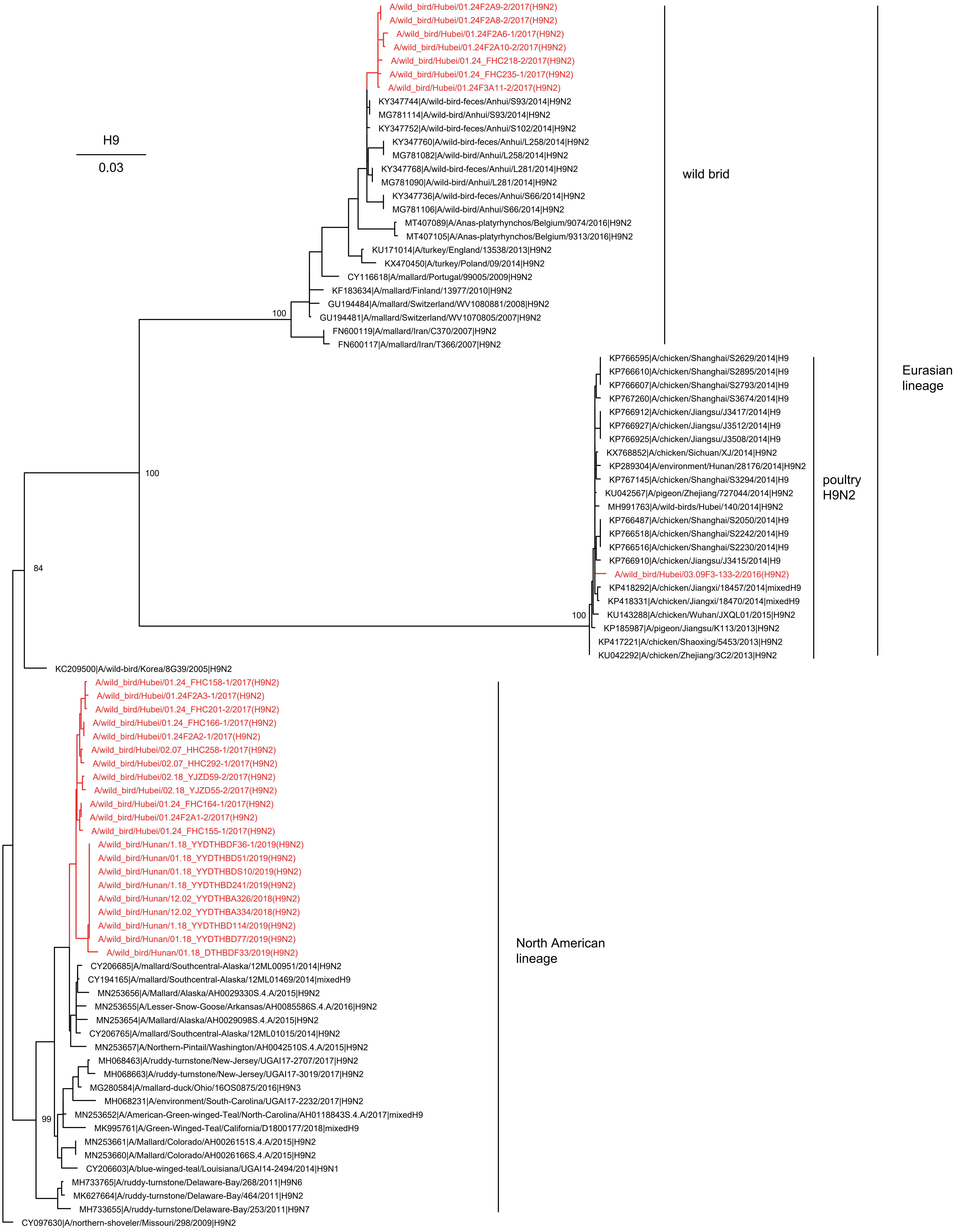

H9
0.03
A/wild_bird/Hubei/01.24F2A9-2/2017(H9N2)
A/wild_bird/Hubei/01.24F2A8-2/2017(H9N2)
A/wild_bird/Hubei/01.24F2A6-1/2017(H9N2)
A/wild_bird/Hubei/01.24F2A10-2/2017(H9N2)
A/wild_bird/Hubei/01.24_FHC218-2/2017(H9N2)
A/wild_bird/Hubei/01.24_FHC235-1/2017(H9N2)
A/wild_bird/Hubei/01.24F3A11-2/2017(H9N2)
KY347744|A/wild-bird-feces/Anhui/S93/2014|H9N2
MG781114|A/wild-bird/Anhui/S93/2014|H9N2
KY347752|A/wild-bird-feces/Anhui/S102/2014|H9N2
KY347760|A/wild-bird-feces/Anhui/L258/2014|H9N2
MG781082|A/wild-bird/Anhui/L258/2014|H9N2
KY347768|A/wild-bird-feces/Anhui/L281/2014|H9N2
MG781090|A/wild-bird/Anhui/L281/2014|H9N2
KY347736|A/wild-bird-feces/Anhui/S66/2014|H9N2
MG781106|A/wild-bird/Anhui/S66/2014|H9N2
MT407089|A/Anas-platyrhynchos/Belgium/9074/2016|H9N2
MT407105|A/Anas-platyrhynchos/Belgium/9313/2016|H9N2
KU171014|A/turkey/England/13538/2013|H9N2
KX470450|A/turkey/Poland/09/2014|H9N2
CY116618|A/mallard/Portugal/99005/2009|H9N2
KF183634|A/mallard/Finland/13977/2010|H9N2
GU194484|A/mallard/Switzerland/WV1080881/2008|H9N2
GU194481|A/mallard/Switzerland/WV1070805/2007|H9N2
FN600119|A/mallard/Iran/C370/2007|H9N2
FN600117|A/mallard/Iran/T366/2007|H9N2
100
wild brid
KP766595|A/chicken/Shanghai/S2629/2014|H9
KP766610|A/chicken/Shanghai/S2895/2014|H9
KP766607|A/chicken/Shanghai/S2793/2014|H9
KP767260|A/chicken/Shanghai/S3674/2014|H9
KP766912|A/chicken/Jiangsu/J3417/2014|H9
KP766927|A/chicken/Jiangsu/J3512/2014|H9
KP766925|A/chicken/Jiangsu/J3508/2014|H9
KX768852|A/chicken/Sichuan/XJ/2014|H9N2
KP289304|A/environment/Hunan/28176/2014|H9N2
KP767145|A/chicken/Shanghai/S3294/2014|H9
KU042567|A/pigeon/Zhejiang/727044/2014|H9N2
MH991763|A/wild-birds/Hubei/140/2014|H9N2
KP766487|A/chicken/Shanghai/S2050/2014|H9
KP766518|A/chicken/Shanghai/S2242/2014|H9
KP766516|A/chicken/Shanghai/S2230/2014|H9
KP766910|A/chicken/Jiangsu/J3415/2014|H9
A/wild_bird/Hubei/03.09F3-133-2/2016(H9N2)
KP418292|A/chicken/Jiangxi/18457/2014|mixedH9
KP418331|A/chicken/Jiangxi/18470/2014|mixedH9
KU143288|A/chicken/Wuhan/JXQL01/2015|H9N2
KP185987|A/pigeon/Jiangsu/K113/2013|H9N2
KP417221|A/chicken/Shaoxing/5453/2013|H9N2
KU042292|A/chicken/Zhejiang/3C2/2013|H9N2
100
100
poultry
H9N2
Eurasian
lineage
84
KC209500|A/wild-bird/Korea/8G39/2005|H9N2
A/wild_bird/Hubei/01.24_FHC158-1/2017(H9N2)
A/wild_bird/Hubei/01.24F2A3-1/2017(H9N2)
A/wild_bird/Hubei/01.24_FHC201-2/2017(H9N2)
A/wild_bird/Hubei/01.24_FHC166-1/2017(H9N2)
A/wild_bird/Hubei/01.24F2A2-1/2017(H9N2)
A/wild_bird/Hubei/02.07_HHC258-1/2017(H9N2)
A/wild_bird/Hubei/02.07_HHC292-1/2017(H9N2)
A/wild_bird/Hubei/02.18_YJZD59-2/2017(H9N2)
A/wild_bird/Hubei/02.18_YJZD55-2/2017(H9N2)
A/wild_bird/Hubei/01.24_FHC164-1/2017(H9N2)
A/wild_bird/Hubei/01.24F2A1-2/2017(H9N2)
A/wild_bird/Hubei/01.24_FHC155-1/2017(H9N2)
A/wild_bird/Hunan/1.18_YYDTHBDF36-1/2019(H9N2)
A/wild_bird/Hunan/01.18_YYDTHBD51/2019(H9N2)
A/wild_bird/Hunan/01.18_YYDTHBDS10/2019(H9N2)
A/wild_bird/Hunan/1.18_YYDTHBD241/2019(H9N2)
A/wild_bird/Hunan/12.02_YYDTHBA326/2018(H9N2)
A/wild_bird/Hunan/12.02_YYDTHBA334/2018(H9N2)
A/wild_bird/Hunan/1.18_YYDTHBD114/2019(H9N2)
A/wild_bird/Hunan/01.18_YYDTHBD77/2019(H9N2)
A/wild_bird/Hunan/01.18_DTHBDF33/2019(H9N2)
CY206685|A/mallard/Southcentral-Alaska/12ML00951/2014|H9N2
CY194165|A/mallard/Southcentral-Alaska/12ML01469/2014|mixedH9
MN253656|A/Mallard/Alaska/AH0029330S.4.A/2015|H9N2
MN253655|A/Lesser-Snow-Goose/Arkansas/AH0085586S.4.A/2016|H9N2
MN253654|A/Mallard/Alaska/AH0029098S.4.A/2015|H9N2
CY206765|A/mallard/Southcentral-Alaska/12ML01015/2014|H9N2
MN253657|A/Northern-Pintail/Washington/AH0042510S.4.A/2015|H9N2
MH068463|A/ruddy-turnstone/New-Jersey/UGAI17-2707/2017|H9N2
MH068663|A/ruddy-turnstone/New-Jersey/UGAI17-3019/2017|H9N2
MG280584|A/mallard-duck/Ohio/16OS0875/2016|H9N3
MH068231|A/environment/South-Carolina/UGAI17-2232/2017|H9N2
MN253652|A/American-Green-winged-Teal/North-Carolina/AH0118843S.4.A/2017|mixedH9
MK995761|A/Green-Winged-Teal/California/D1800177/2018|mixedH9
MN253661|A/Mallard/Colorado/AH0026151S.4.A/2015|H9N2
MN253660|A/Mallard/Colorado/AH0026166S.4.A/2015|H9N2
CY206603|A/blue-winged-teal/Louisiana/UGAI14-2494/2014|H9N1
MH733765|A/ruddy-turnstone/Delaware-Bay/268/2011|H9N6
MK627664|A/ruddy-turnstone/Delaware-Bay/464/2011|H9N2
MH733655|A/ruddy-turnstone/Delaware-Bay/253/2011|H9N7
99
North American
lineage
CY097630|A/northern-shoveler/Missouri/298/2009|H9N2

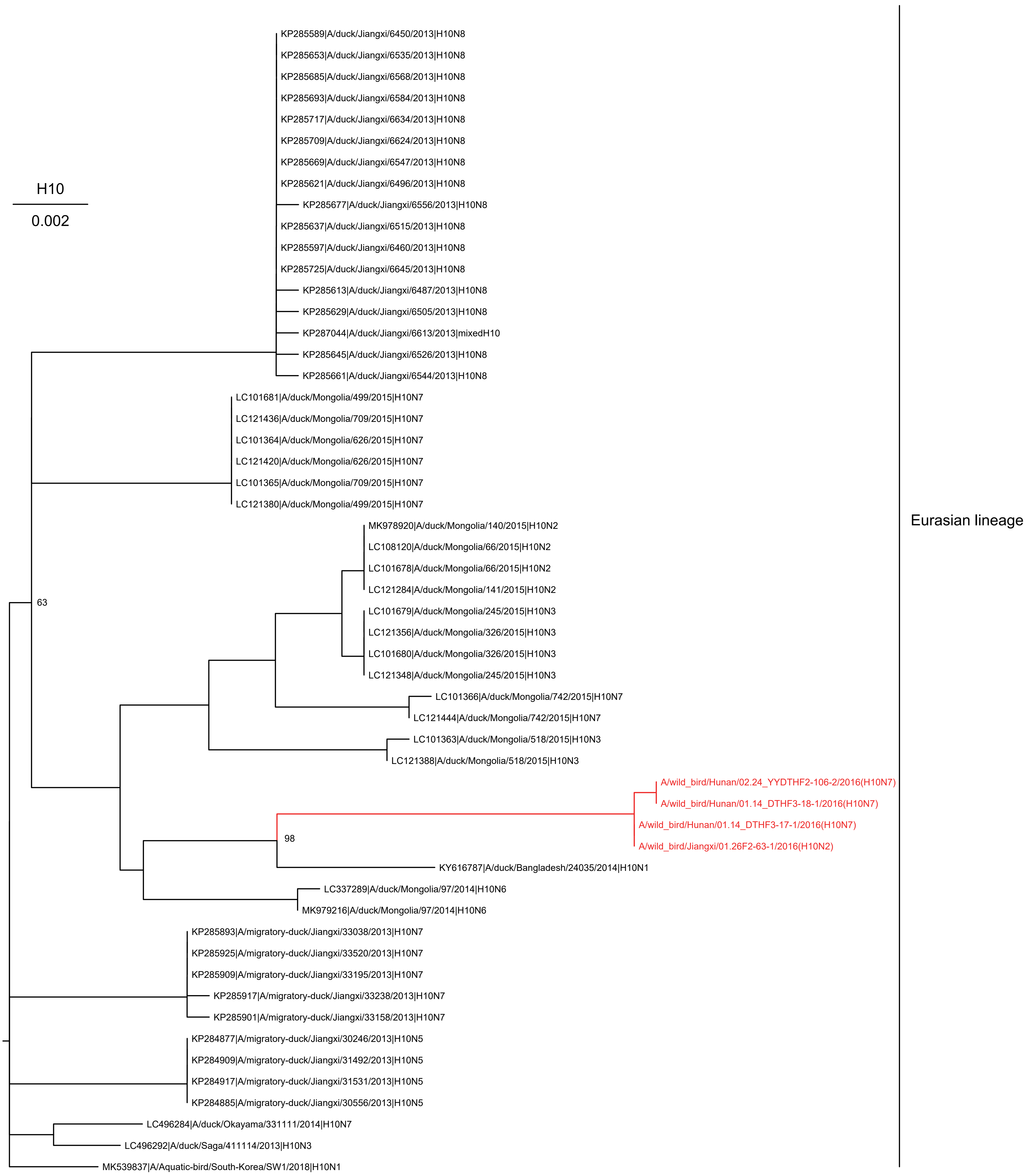
H10
0.002
KP285589|A/duck/Jiangxi/6450/2013|H10N8
KP285653|A/duck/Jiangxi/6535/2013|H10N8
KP285685|A/duck/Jiangxi/6568/2013|H10N8
KP285693|A/duck/Jiangxi/6584/2013|H10N8
KP285717|A/duck/Jiangxi/6634/2013|H10N8
KP285709|A/duck/Jiangxi/6624/2013|H10N8
KP285669|A/duck/Jiangxi/6547/2013|H10N8
KP285621|A/duck/Jiangxi/6496/2013|H10N8
KP285677|A/duck/Jiangxi/6556/2013|H10N8
KP285637|A/duck/Jiangxi/6515/2013|H10N8
KP285597|A/duck/Jiangxi/6460/2013|H10N8
KP285725|A/duck/Jiangxi/6645/2013|H10N8
KP285613|A/duck/Jiangxi/6487/2013|H10N8
KP285629|A/duck/Jiangxi/6505/2013|H10N8
KP287044|A/duck/Jiangxi/6613/2013|mixedH10
KP285645|A/duck/Jiangxi/6526/2013|H10N8
KP285661|A/duck/Jiangxi/6544/2013|H10N8
LC101681|A/duck/Mongolia/499/2015|H10N7
LC121436|A/duck/Mongolia/709/2015|H10N7
LC101364|A/duck/Mongolia/626/2015|H10N7
LC121420|A/duck/Mongolia/626/2015|H10N7
LC101365|A/duck/Mongolia/709/2015|H10N7
LC121380|A/duck/Mongolia/499/2015|H10N7
MK978920|A/duck/Mongolia/140/2015|H10N2
LC108120|A/duck/Mongolia/66/2015|H10N2
LC101678|A/duck/Mongolia/66/2015|H10N2
LC121284|A/duck/Mongolia/141/2015|H10N2
LC101679|A/duck/Mongolia/245/2015|H10N3
LC121356|A/duck/Mongolia/326/2015|H10N3
LC101680|A/duck/Mongolia/326/2015|H10N3
LC121348|A/duck/Mongolia/245/2015|H10N3
63
LC101366|A/duck/Mongolia/742/2015|H10N7
LC121444|A/duck/Mongolia/742/2015|H10N7
LC101363|A/duck/Mongolia/518/2015|H10N3
LC121388|A/duck/Mongolia/518/2015|H10N3
A/wild_bird/Hunan/02.24_YYDTHF2-106-2/2016(H10N7)
A/wild_bird/Hunan/01.14_DTHF3-18-1/2016(H10N7)
A/wild_bird/Hunan/01.14_DTHF3-17-1/2016(H10N7)
A/wild_bird/Jiangxi/01.26F2-63-1/2016(H10N2)
98
KY616787|A/duck/Bangladesh/24035/2014|H10N1
LC337289|A/duck/Mongolia/97/2014|H10N6
MK979216|A/duck/Mongolia/97/2014|H10N6
KP285893|A/migratory-duck/Jiangxi/33038/2013|H10N7
KP285925|A/migratory-duck/Jiangxi/33520/2013|H10N7
KP285909|A/migratory-duck/Jiangxi/33195/2013|H10N7
KP285917|A/migratory-duck/Jiangxi/33238/2013|H10N7
KP285901|A/migratory-duck/Jiangxi/33158/2013|H10N7
KP284877|A/migratory-duck/Jiangxi/30246/2013|H10N5
KP284909|A/migratory-duck/Jiangxi/31492/2013|H10N5
KP284917|A/migratory-duck/Jiangxi/31531/2013|H10N5
KP284885|A/migratory-duck/Jiangxi/30556/2013|H10N5
LC496284|A/duck/Okayama/331111/2014|H10N7
LC496292|A/duck/Saga/411114/2013|H10N3
MK539837|A/Aquatic-bird/South-Korea/SW1/2018|H10N1
Eurasian lineage

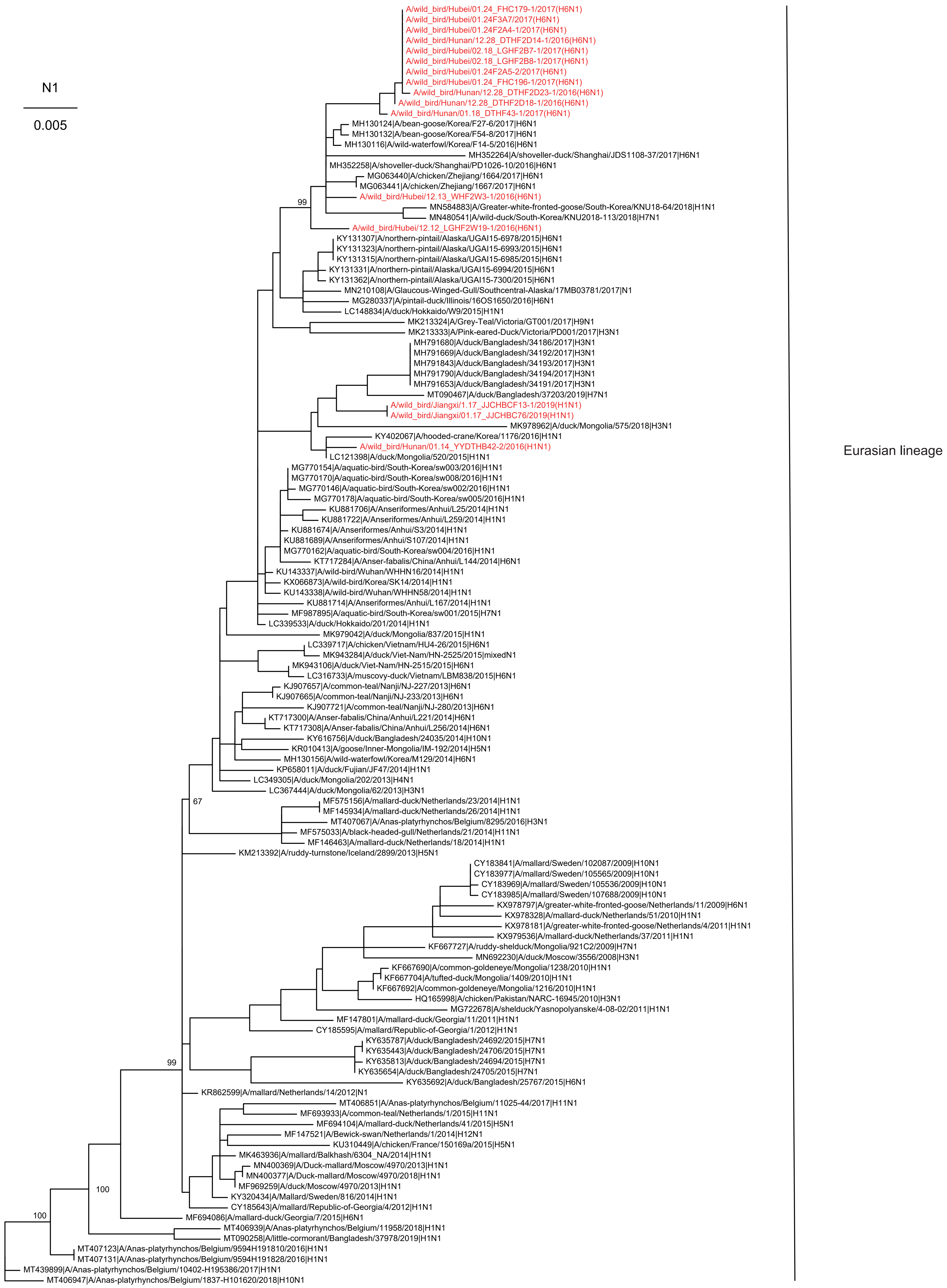

N1
0.005
A/wild_bird/Hubei/01.24_FHC179-1/2017(H6N1)
A/wild_bird/Hubei/01.24F3A7/2017(H6N1)
A/wild_bird/Hubei/01.24F2A4-1/2017(H6N1)
A/wild_bird/Hunan/12.28_DTHF2D14-1/2016(H6N1)
A/wild_bird/Hubei/02.18_LGHF2B7-1/2017(H6N1)
A/wild_bird/Hubei/02.18_LGHF2B8-1/2017(H6N1)
A/wild_bird/Hubei/01.24F2A5-2/2017(H6N1)
A/wild_bird/Hubei/01.24_FHC196-1/2017(H6N1)
A/wild_bird/Hunan/12.28_DTHF2D23-1/2016(H6N1)
A/wild_bird/Hunan/12.28_DTHF2D18-1/2016(H6N1)
A/wild_bird/Hunan/01.18_DTHF43-1/2017(H6N1)
MH130124|A/bean-goose/Korea/F27-6/2017|H6N1
MH130132|A/bean-goose/Korea/F54-8/2017|H6N1
MH130116|A/wild-waterfowl/Korea/F14-5/2016|H6N1
MH352264|A/shoveller-duck/Shanghai/JDS1108-37/2017|H6N1
MH352258|A/shoveller-duck/Shanghai/PD1026-10/2016|H6N1
MG063440|A/chicken/Zhejiang/1664/2017|H6N1
MG063441|A/chicken/Zhejiang/1667/2017|H6N1
A/wild_bird/Hubei/12.13_WHF2W3-1/2016(H6N1)
99
MN584883|A/Greater-white-fronted-goose/South-Korea/KNU18-64/2018|H1N1
MN480541|A/wild-duck/South-Korea/KNU2018-113/2018|H7N1
A/wild_bird/Hubei/12.12_LGHF2W19-1/2016(H6N1)
KY131307|A/northern-pintail/Alaska/UGAI15-6978/2015|H6N1
KY131323|A/northern-pintail/Alaska/UGAI15-6993/2015|H6N1
KY131315|A/northern-pintail/Alaska/UGAI15-6985/2015|H6N1
KY131331|A/northern-pintail/Alaska/UGAI15-6994/2015|H6N1
KY131362|A/northern-pintail/Alaska/UGAI15-7300/2015|H6N1
MN210108|A/Glaucous-Winged-Gull/Southcentral-Alaska/17MB03781/2017|N1
MG280337|A/pintail-duck/Illinois/16OS1650/2016|H6N1
LC148834|A/duck/Hokkaido/W9/2015|H1N1
MK213324|A/Grey-Teal/Victoria/GT001/2017|H9N1
MK213333|A/Pink-eared-Duck/Victoria/PD001/2017|H3N1
MH791680|A/duck/Bangladesh/34186/2017|H3N1
MH791669|A/duck/Bangladesh/34192/2017|H3N1
MH791843|A/duck/Bangladesh/34193/2017|H3N1
MH791790|A/duck/Bangladesh/34194/2017|H3N1
MH791653|A/duck/Bangladesh/34191/2017|H3N1
MT090467|A/duck/Bangladesh/37203/2019|H7N1
A/wild_bird/Jiangxi/1.17_JJCHBCF13-1/2019(H1N1)
A/wild_bird/Jiangxi/01.17_JJCHBC76/2019(H1N1)
MK978962|A/duck/Mongolia/575/2018|H3N1
KY402067|A/hooded-crane/Korea/1176/2016|H1N1
A/wild_bird/Hunan/01.14_YYDTHB42-2/2016(H1N1)
LC121398|A/duck/Mongolia/520/2015|H1N1
MG770154|A/aquatic-bird/South-Korea/sw003/2016|H1N1
MG770170|A/aquatic-bird/South-Korea/sw008/2016|H1N1
MG770146|A/aquatic-bird/South-Korea/sw002/2016|H1N1
MG770178|A/aquatic-bird/South-Korea/sw005/2016|H1N1
KU881706|A/Anseriformes/Anhui/L25/2014|H1N1
KU881722|A/Anseriformes/Anhui/L259/2014|H1N1
KU881674|A/Anseriformes/Anhui/S3/2014|H1N1
KU881689|A/Anseriformes/Anhui/S107/2014|H1N1
MG770162|A/aquatic-bird/South-Korea/sw004/2016|H1N1
KT717284|A/Anser-fabalis/China/Anhui/L144/2014|H6N1
KU143337|A/wild-bird/Wuhan/WHHN16/2014|H1N1
KX066873|A/wild-bird/Korea/SK14/2014|H1N1
KU143338|A/wild-bird/Wuhan/WHHN58/2014|H1N1
KU881714|A/Anseriformes/Anhui/L167/2014|H1N1
MF987895|A/aquatic-bird/South-Korea/sw001/2015|H7N1
LC339533|A/duck/Hokkaido/201/2014|H1N1
MK979042|A/duck/Mongolia/837/2015|H1N1
LC339717|A/chicken/Vietnam/HU4-26/2015|H6N1
MK943284|A/duck/Viet-Nam/HN-2525/2015|mixedN1
MK943106|A/duck/Viet-Nam/HN-2515/2015|H6N1
LC316733|A/muscovy-duck/Vietnam/LBM838/2015|H6N1
KJ907657|A/common-teal/Nanji/NJ-227/2013|H6N1
KJ907665|A/common-teal/Nanji/NJ-233/2013|H6N1
KJ907721|A/common-teal/Nanji/NJ-280/2013|H6N1
KT717300|A/Anser-fabalis/China/Anhui/L221/2014|H6N1
KT717308|A/Anser-fabalis/China/Anhui/L256/2014|H6N1
KY616756|A/duck/Bangladesh/24035/2014|H10N1
KR010413|A/goose/Inner-Mongolia/IM-192/2014|H5N1
MH130156|A/wild-waterfowl/Korea/M129/2014|H6N1
KP658011|A/duck/Fujian/JF47/2014|H1N1
LC349305|A/duck/Mongolia/202/2013|H4N1
LC367444|A/duck/Mongolia/62/2013|H3N1
67
MF575156|A/mallard-duck/Netherlands/23/2014|H1N1
MF145934|A/mallard-duck/Netherlands/26/2014|H1N1
MT407067|A/Anas-platyrhynchos/Belgium/8295/2016|H3N1
MF575033|A/black-headed-gull/Netherlands/21/2014|H11N1
MF146463|A/mallard-duck/Netherlands/18/2014|H1N1
KM213392|A/ruddy-turnstone/Iceland/2899/2013|H5N1
CY183841|A/mallard/Sweden/102087/2009|H10N1
CY183977|A/mallard/Sweden/105565/2009|H10N1
CY183969|A/mallard/Sweden/105536/2009|H10N1
CY183985|A/mallard/Sweden/107688/2009|H10N1
KX978797|A/greater-white-fronted-goose/Netherlands/11/2009|H6N1
KX978328|A/mallard-duck/Netherlands/51/2010|H1N1
KX978181|A/greater-white-fronted-goose/Netherlands/4/2011|H1N1
KX979536|A/mallard-duck/Netherlands/37/2011|H1N1
KF667727|A/ruddy-shelduck/Mongolia/921C2/2009|H7N1
MN692230|A/duck/Moscow/3556/2008|H3N1
KF667690|A/common-goldeneye/Mongolia/1238/2010|H1N1
KF667704|A/tufted-duck/Mongolia/1409/2010|H1N1
KF667692|A/common-goldeneye/Mongolia/1216/2010|H1N1
HQ165998|A/chicken/Pakistan/NARC-16945/2010|H3N1
MG722678|A/shelduck/Yasnopolyanske/4-08-02/2011|H1N1
MF147801|A/mallard-duck/Georgia/11/2011|H1N1
CY185595|A/mallard/Republic-of-Georgia/1/2012|H1N1
KY635787|A/duck/Bangladesh/24692/2015|H7N1
KY635443|A/duck/Bangladesh/24706/2015|H7N1
KY635813|A/duck/Bangladesh/24694/2015|H7N1
KY635654|A/duck/Bangladesh/24705/2015|H7N1
KY635692|A/duck/Bangladesh/25767/2015|H6N1
99
KR862599|A/mallard/Netherlands/14/2012|N1
MT406851|A/Anas-platyrhynchos/Belgium/11025-44/2017|H11N1
MF693933|A/common-teal/Netherlands/1/2015|H11N1
MF694104|A/mallard-duck/Netherlands/41/2015|H5N1
MF147521|A/Bewick-swan/Netherlands/1/2014|H12N1
KU310449|A/chicken/France/150169a/2015|H5N1
MK463936|A/mallard/Balkhash/6304_NA/2014|H1N1
MN400369|A/Duck-mallard/Moscow/4970/2013|H1N1
MN400377|A/Duck-mallard/Moscow/4970/2018|H1N1
MF969259|A/duck/Moscow/4970/2013|H1N1
KY320434|A/Mallard/Sweden/816/2014|H1N1
CY185643|A/mallard/Republic-of-Georgia/4/2012|H1N1
100
MF694086|A/mallard-duck/Georgia/7/2015|H6N1
100
MT406939|A/Anas-platyrhynchos/Belgium/11958/2018|H1N1
MT090258|A/little-cormorant/Bangladesh/37978/2019|H1N1
MT407123|A/Anas-platyrhynchos/Belgium/9594H191810/2016|H1N1
MT407131|A/Anas-platyrhynchos/Belgium/9594H191828/2016|H1N1
MT439899|A/Anas-platyrhynchos/Belgium/10402-H195386/2017|H1N1
MT406947|A/Anas-platyrhynchos/Belgium/1837-H101620/2018|H10N1
Eurasian lineage

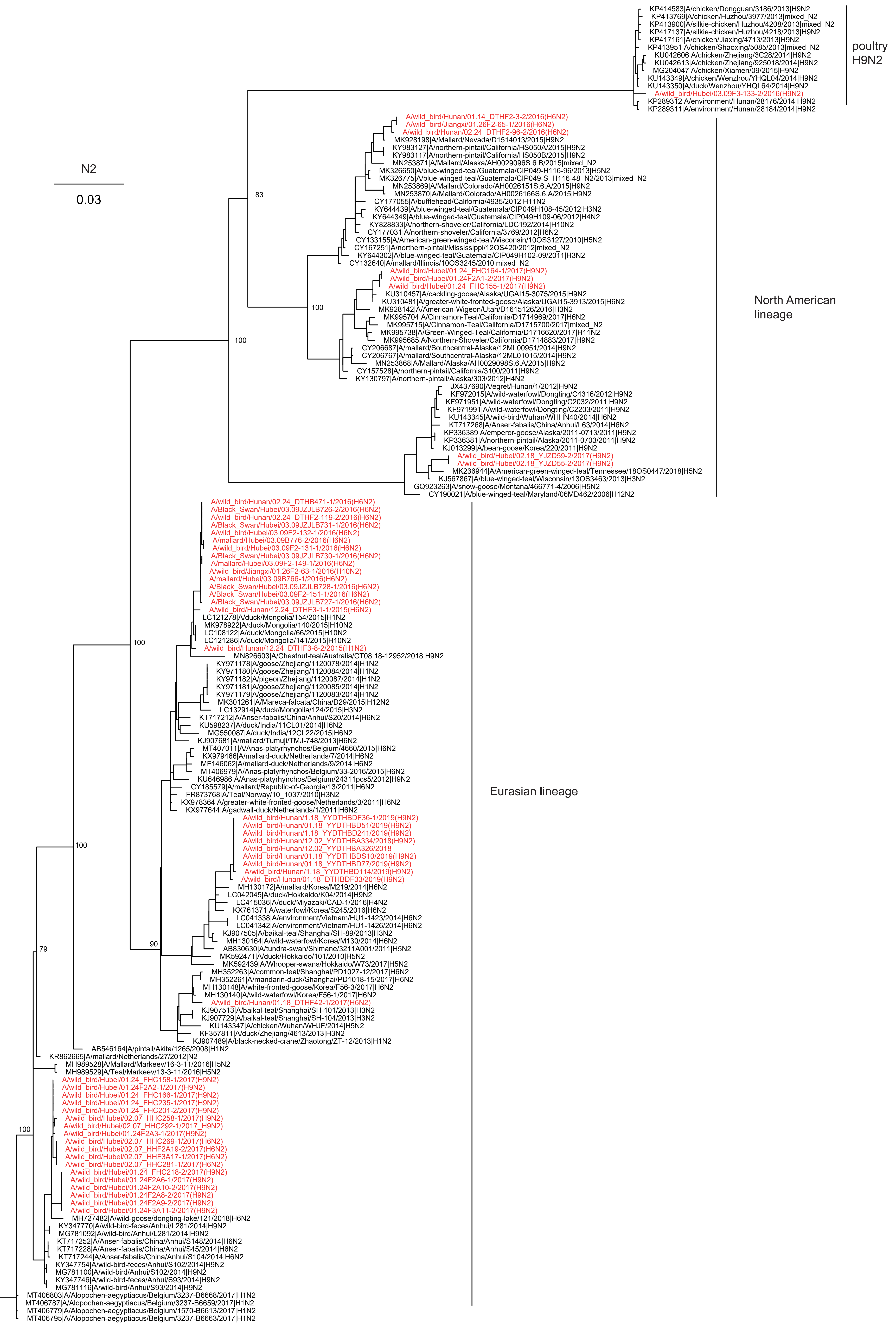

N2
0.03
poultry H9N2
North American lineage
Eurasian lineage
83
100
100
100
100
90
79
100
KP414583|A/chicken/Dongguan/3186/2013|H9N2
KP413769|A/chicken/Huzhou/3977/2013|mixed_N2
KP413900|A/silkie-chicken/Huzhou/4208/2013|mixed_N2
KP417137|A/silkie-chicken/Huzhou/4218/2013|H9N2
KP417161|A/chicken/Jiaxing/4713/2013|H9N2
KP413951|A/chicken/Shaoxing/5085/2013|mixed_N2
KU042606|A/chicken/Zhejiang/3C28/2014|H9N2
KU042613|A/chicken/Zhejiang/925018/2014|H9N2
MG204047|A/chicken/Xiamen/09/2015|H9N2
KU143349|A/chicken/Wenzhou/YHQL04/2014|H9N2
KU143350|A/duck/Wenzhou/YHQL64/2014|H9N2
A/wild_bird/Hubei/03.09F3-133-2/2016(H9N2)
KP289312|A/environment/Hunan/28176/2014|H9N2
KP289311|A/environment/Hunan/28184/2014|H9N2
A/wild_bird/Hunan/01.14_DTHF2-3-2/2016(H6N2)
A/wild_bird/Jiangxi/01.26F2-65-1/2016(H6N2)
A/wild_bird/Hunan/02.24_DTHF2-96-2/2016(H6N2)
MK928198|A/Mallard/Nevada/D1514013/2015|H9N2
KY983127|A/northern-pintail/California/HS050A/2015|H9N2
KY983117|A/northern-pintail/California/HS050B/2015|H9N2
MN253871|A/Mallard/Alaska/AH0029096S.6.B/2015|mixed_N2
MK326650|A/blue-winged-teal/Guatemala/CIP049-H116-96/2013|H5N2
MK326775|A/blue-winged-teal/Guatemala/CIP049-S_H116-48_N2/2013|mixed_N2
MN253869|A/Mallard/Colorado/AH0026151S.6.A/2015|H9N2
MN253870|A/Mallard/Colorado/AH0026166S.6.A/2015|H9N2
CY177055|A/bufflehead/California/4935/2012|H11N2
KY644439|A/blue-winged-teal/Guatemala/CIP049H108-45/2012|H3N2
KY644349|A/blue-winged-teal/Guatemala/CIP049H109-06/2012|H4N2
KY828833|A/northern-shoveler/California/LDC192/2014|H10N2
CY177031|A/northern-shoveler/California/3769/2012|H6N2
CY133155|A/American-green-winged-teal/Wisconsin/10OS3127/2010|H5N2
CY167251|A/northern-pintail/Mississippi/12OS420/2012|mixed_N2
KY644302|A/blue-winged-teal/Guatemala/CIP049H102-09/2011|H3N2
CY132640|A/mallard/Illinois/10OS3245/2010|mixed_N2
A/wild_bird/Hubei/01.24_FHC164-1/2017(H9N2)
A/wild_bird/Hubei/01.24F2A1-2/2017(H9N2)
A/wild_bird/Hubei/01.24_FHC155-1/2017(H9N2)
KU310457|A/cackling-goose/Alaska/UGAI15-3075/2015|H9N2
KU310481|A/greater-white-fronted-goose/Alaska/UGAI15-3913/2015|H6N2
MK928142|A/American-Wigeon/Utah/D1615126/2016|H3N2
MK995704|A/Cinnamon-Teal/California/D1714969/2017|H6N2
MK995715|A/Cinnamon-Teal/California/D1715700/2017|mixed_N2
MK995738|A/Green-Winged-Teal/California/D1716620/2017|H11N2
MK995685|A/Northern-Shoveler/California/D1714883/2017|H9N2
CY206687|A/mallard/Southcentral-Alaska/12ML00951/2014|H9N2
CY206767|A/mallard/Southcentral-Alaska/12ML01015/2014|H9N2
MN253868|A/Mallard/Alaska/AH0029098S.6.A/2015|H9N2
CY157528|A/northern-pintail/California/3100/2011|H9N2
KY130797|A/northern-pintail/Alaska/303/2012|H4N2
JX437690|A/egret/Hunan/1/2012|H9N2
KF972015|A/wild-waterfowl/Dongting/C4316/2012|H9N2
KF971951|A/wild-waterfowl/Dongting/C2032/2011|H9N2
KF971991|A/wild-waterfowl/Dongting/C2203/2011|H9N2
KU143345|A/wild-bird/Wuhan/WHHN40/2014|H6N2
KT717268|A/Anser-fabalis/China/Anhui/L63/2014|H6N2
KP336389|A/emperor-goose/Alaska/2011-0713/2011|H9N2
KP336381|A/northern-pintail/Alaska/2011-0703/2011|H9N2
KJ013299|A/bean-goose/Korea/220/2011|H9N2
A/wild_bird/Hubei/02.18_YJZD59-2/2017(H9N2)
A/wild_bird/Hubei/02.18_YJZD55-2/2017(H9N2)
MK236944|A/American-green-winged-teal/Tennessee/18OS0447/2018|H5N2
KJ567867|A/blue-winged-teal/Wisconsin/13OS3463/2013|H3N2
GQ923263|A/snow-goose/Montana/466771-4/2006|H5N2
CY190021|A/blue-winged-teal/Maryland/06MD462/2006|H12N2
A/wild_bird/Hunan/02.24_DTHB471-1/2016(H6N2)
A/Black_Swan/Hubei/03.09JZJLB726-2/2016(H6N2)
A/wild_bird/Hunan/02.24_DTHF2-119-2/2016(H6N2)
A/Black_Swan/Hubei/03.09JZJLB731-1/2016(H6N2)
A/wild_bird/Hubei/03.09F2-132-1/2016(H6N2)
A/mallard/Hubei/03.09B776-2/2016(H6N2)
A/wild_bird/Hubei/03.09F2-131-1/2016(H6N2)
A/Black_Swan/Hubei/03.09JZJLB730-1/2016(H6N2)
A/mallard/Hubei/03.09F2-149-1/2016(H6N2)
A/wild_bird/Jiangxi/01.26F2-63-1/2016(H10N2)
A/mallard/Hubei/03.09B766-1/2016(H6N2)
A/Black_Swan/Hubei/03.09JZJLB728-1/2016(H6N2)
A/Black_Swan/Hubei/03.09F2-151-1/2016(H6N2)
A/Black_Swan/Hubei/03.09JZJLB727-1/2016(H6N2)
A/wild_bird/Hunan/12.24_DTHF3-1-1/2015(H6N2)
LC121278|A/duck/Mongolia/154/2015|H1N2
MK978922|A/duck/Mongolia/140/2015|H10N2
LC108122|A/duck/Mongolia/66/2015|H10N2
LC121286|A/duck/Mongolia/141/2015|H10N2
A/wild_bird/Hunan/12.24_DTHF3-8-2/2015(H1N2)
MN826603|A/Chestnut-teal/Australia/CT08.18-12952/2018|H9N2
KY971178|A/goose/Zhejiang/1120078/2014|H1N2
KY971180|A/goose/Zhejiang/1120084/2014|H1N2
KY971182|A/pigeon/Zhejiang/1120087/2014|H1N2
KY971181|A/goose/Zhejiang/1120085/2014|H1N2
KY971179|A/goose/Zhejiang/1120083/2014|H1N2
MK301261|A/Mareca-falcata/China/D29/2015|H12N2
LC132914|A/duck/Mongolia/124/2015|H3N2
KT717212|A/Anser-fabalis/China/Anhui/S20/2014|H6N2
KU598237|A/duck/India/11CL01/2014|H6N2
MG550087|A/duck/India/12CL22/2015|H6N2
KJ907681|A/mallard/Tumuji/TMJ-748/2013|H6N2
MT407011|A/Anas-platyrhynchos/Belgium/4660/2015|H6N2
KX979466|A/mallard-duck/Netherlands/7/2014|H6N2
MF146062|A/mallard-duck/Netherlands/9/2014|H6N2
MT406979|A/Anas-platyrhynchos/Belgium/33-2016/2015|H6N2
KU646986|A/Anas-platyrhynchos/Belgium/24311pcs5/2012|H9N2
CY185579|A/mallard/Republic-of-Georgia/13/2011|H6N2
FR873768|A/Teal/Norway/10_1037/2010|H3N2
KX978364|A/greater-white-fronted-goose/Netherlands/3/2011|H6N2
KX977644|A/gadwall-duck/Netherlands/1/2011|H6N2
A/wild_bird/Hunan/1.18_YYDTHBDF36-1/2019(H9N2)
A/wild_bird/Hunan/01.18_YYDTHBD51/2019(H9N2)
A/wild_bird/Hunan/1.18_YYDTHBD241/2019(H9N2)
A/wild_bird/Hunan/12.02_YYDTHBA334/2018(H9N2)
A/wild_bird/Hunan/12.02_YYDTHBA326/2018
A/wild_bird/Hunan/01.18_YYDTHBDS10/2019(H9N2)
A/wild_bird/Hunan/01.18_YYDTHBD77/2019(H9N2)
A/wild_bird/Hunan/1.18_YYDTHBD114/2019(H9N2)
A/wild_bird/Hunan/01.18_DTHBDF33/2019(H9N2)
MH130172|A/mallard/Korea/M219/2014|H6N2
LC042045|A/duck/Hokkaido/K04/2014|H9N2
LC415036|A/duck/Miyazaki/CAD-1/2016|H4N2
KX761371|A/waterfowl/Korea/S245/2016|H6N2
LC041338|A/environment/Vietnam/HU1-1423/2014|H6N2
LC041342|A/environment/Vietnam/HU1-1426/2014|H6N2
KJ907505|A/baikal-teal/Shanghai/SH-89/2013|H3N2
MH130164|A/wild-waterfowl/Korea/M130/2014|H6N2
AB830630|A/tundra-swan/Shimane/3211A001/2011|H5N2
MK592471|A/duck/Hokkaido/101/2010|H5N2
MK592439|A/Whooper-swans/Hokkaido/W73/2017|H5N2
MH352263|A/common-teal/Shanghai/PD1027-12/2017|H6N2
MH352261|A/mandarin-duck/Shanghai/PD1018-15/2017|H6N2
MH130148|A/white-fronted-goose/Korea/F56-3/2017|H6N2
MH130140|A/wild-waterfowl/Korea/F56-1/2017|H6N2
A/wild_bird/Hunan/01.18_DTHF42-1/2017(H6N2)
KJ907513|A/baikal-teal/Shanghai/SH-101/2013|H3N2
KJ907729|A/baikal-teal/Shanghai/SH-104/2013|H3N2
KU143347|A/chicken/Wuhan/WHJF/2014|H5N2
KF357811|A/duck/Zhejiang/4613/2013|H3N2
KJ907489|A/black-necked-crane/Zhaotong/ZT-12/2013|H1N2
AB546164|A/pintail/Akita/1265/2008|H1N2
KR862665|A/mallard/Netherlands/27/2012|N2
MH989528|A/Mallard/Markeev/16-3-11/2016|H5N2
MH989529|A/Teal/Markeev/13-3-11/2016|H5N2
A/wild_bird/Hubei/01.24_FHC158-1/2017(H9N2)
A/wild_bird/Hubei/01.24F2A2-1/2017(H9N2)
A/wild_bird/Hubei/01.24_FHC166-1/2017(H9N2)
A/wild_bird/Hubei/01.24_FHC235-1/2017(H9N2)
A/wild_bird/Hubei/01.24_FHC201-2/2017(H9N2)
A/wild_bird/Hubei/02.07_HHC258-1/2017(H9N2)
A/wild_bird/Hubei/02.07_HHC292-1/2017_H9N2)
A/wild_bird/Hubei/01.24F2A3-1/2017(H9N2)
A/wild_bird/Hubei/02.07_HHC269-1/2017(H6N2)
A/wild_bird/Hubei/02.07_HHF2A19-2/2017(H6N2)
A/wild_bird/Hubei/02.07_HHF3A17-1/2017(H6N2)
A/wild_bird/Hubei/02.07_HHC281-1/2017(H6N2)
A/wild_bird/Hubei/01.24_FHC218-2/2017(H9N2)
A/wild_bird/Hubei/01.24F2A6-1/2017(H9N2)
A/wild_bird/Hubei/01.24F2A10-2/2017(H9N2)
A/wild_bird/Hubei/01.24F2A8-2/2017(H9N2)
A/wild_bird/Hubei/01.24F2A9-2/2017(H9N2)
A/wild_bird/Hubei/01.24F3A11-2/2017(H9N2)
MH727482|A/wild-goose/dongting-lake/121/2018|H6N2
KY347770|A/wild-bird-feces/Anhui/L281/2014|H9N2
MG781092|A/wild-bird/Anhui/L281/2014|H9N2
KT717252|A/Anser-fabalis/China/Anhui/S148/2014|H6N2
KT717228|A/Anser-fabalis/China/Anhui/S45/2014|H6N2
KT717244|A/Anser-fabalis/China/Anhui/S104/2014|H6N2
KY347754|A/wild-bird-feces/Anhui/S102/2014|H9N2
MG781100|A/wild-bird/Anhui/S102/2014|H9N2
KY347746|A/wild-bird-feces/Anhui/S93/2014|H9N2
MG781116|A/wild-bird/Anhui/S93/2014|H9N2
MT406803|A/Alopochen-aegyptiacus/Belgium/3237-B6668/2017|H1N2
MT406787|A/Alopochen-aegyptiacus/Belgium/3237-B6659/2017|H1N2
MT406779|A/Alopochen-aegyptiacus/Belgium/1570-B6613/2017|H1N2
MT406795|A/Alopochen-aegyptiacus/Belgium/3237-B6663/2017|H1N2

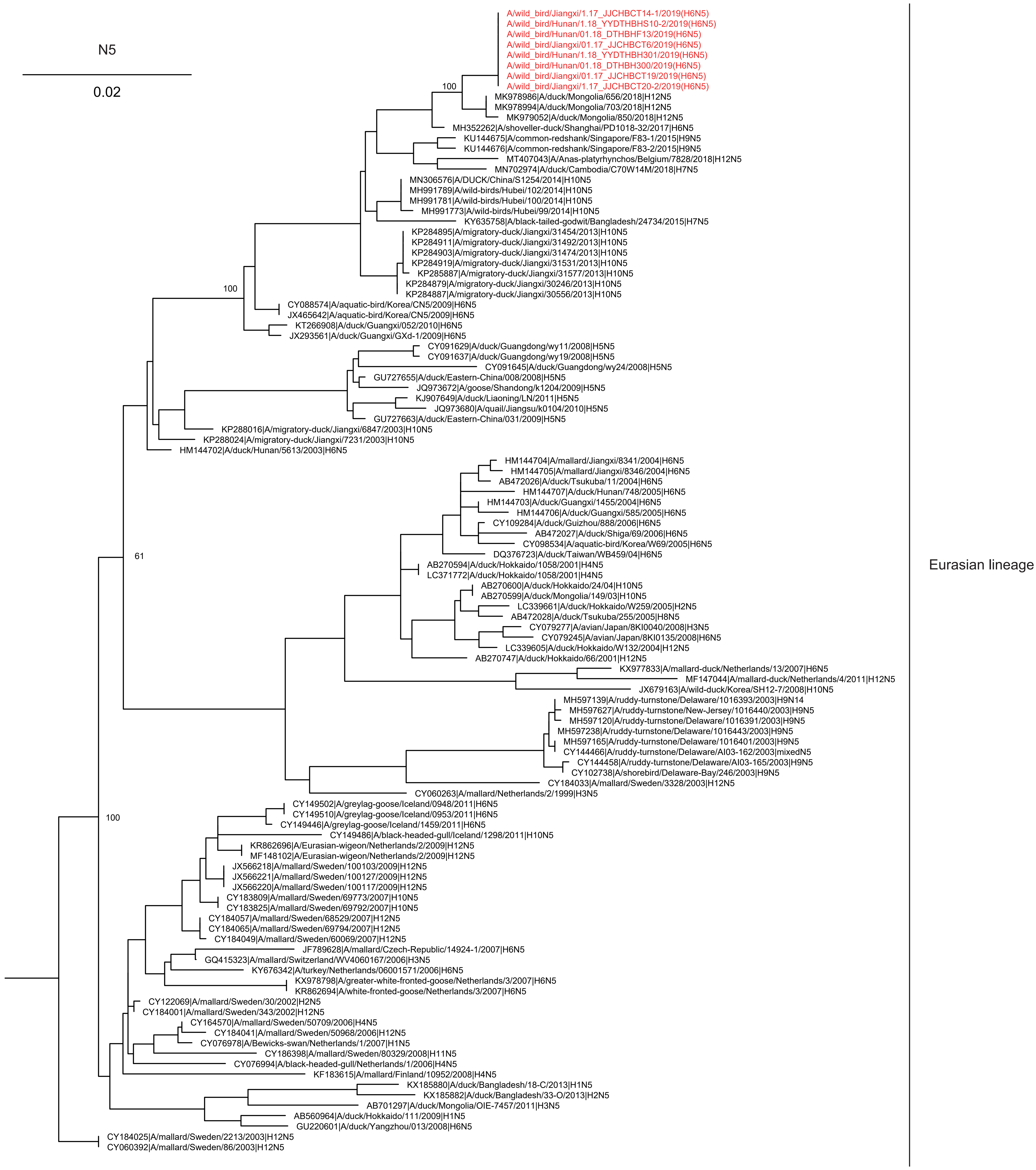
N5
0.02
A/wild_bird/Jiangxi/1.17_JJCHBCT14-1/2019(H6N5)
A/wild_bird/Hunan/1.18_YYDTHBHS10-2/2019(H6N5)
A/wild_bird/Hunan/01.18_DTHBHF13/2019(H6N5)
A/wild_bird/Jiangxi/01.17_JJCHBCT6/2019(H6N5)
A/wild_bird/Hunan/1.18_YYDTHBH301/2019(H6N5)
A/wild_bird/Hunan/01.18_DTHBH300/2019(H6N5)
A/wild_bird/Jiangxi/01.17_JJCHBCT19/2019(H6N5)
A/wild_bird/Jiangxi/1.17_JJCHBCT20-2/2019(H6N5)
100
MK978986|A/duck/Mongolia/656/2018|H12N5
MK978994|A/duck/Mongolia/703/2018|H12N5
MK979052|A/duck/Mongolia/850/2018|H12N5
MH352262|A/shoveller-duck/Shanghai/PD1018-32/2017|H6N5
KU144675|A/common-redshank/Singapore/F83-1/2015|H9N5
KU144676|A/common-redshank/Singapore/F83-2/2015|H9N5
MT407043|A/Anas-platyrhynchos/Belgium/7828/2018|H12N5
MN702974|A/duck/Cambodia/C70W14M/2018|H7N5
MN306576|A/DUCK/China/S1254/2014|H10N5
MH991789|A/wild-birds/Hubei/102/2014|H10N5
MH991781|A/wild-birds/Hubei/100/2014|H10N5
MH991773|A/wild-birds/Hubei/99/2014|H10N5
KY635758|A/black-tailed-godwit/Bangladesh/24734/2015|H7N5
KP284895|A/migratory-duck/Jiangxi/31454/2013|H10N5
KP284911|A/migratory-duck/Jiangxi/31492/2013|H10N5
KP284903|A/migratory-duck/Jiangxi/31474/2013|H10N5
KP284919|A/migratory-duck/Jiangxi/31531/2013|H10N5
KP285887|A/migratory-duck/Jiangxi/31577/2013|H10N5
KP284879|A/migratory-duck/Jiangxi/30246/2013|H10N5
KP284887|A/migratory-duck/Jiangxi/30556/2013|H10N5
100
CY088574|A/aquatic-bird/Korea/CN5/2009|H6N5
JX465642|A/aquatic-bird/Korea/CN5/2009|H6N5
KT266908|A/duck/Guangxi/052/2010|H6N5
JX293561|A/duck/Guangxi/GXd-1/2009|H6N5
CY091629|A/duck/Guangdong/wy11/2008|H5N5
CY091637|A/duck/Guangdong/wy19/2008|H5N5
CY091645|A/duck/Guangdong/wy24/2008|H5N5
GU727655|A/duck/Eastern-China/008/2008|H5N5
JQ973672|A/goose/Shandong/k1204/2009|H5N5
KJ907649|A/duck/Liaoning/LN/2011|H5N5
JQ973680|A/quail/Jiangsu/k0104/2010|H5N5
GU727663|A/duck/Eastern-China/031/2009|H5N5
KP288016|A/migratory-duck/Jiangxi/6847/2003|H10N5
KP288024|A/migratory-duck/Jiangxi/7231/2003|H10N5
HM144702|A/duck/Hunan/5613/2003|H6N5
HM144704|A/mallard/Jiangxi/8341/2004|H6N5
HM144705|A/mallard/Jiangxi/8346/2004|H6N5
AB472026|A/duck/Tsukuba/11/2004|H6N5
HM144707|A/duck/Hunan/748/2005|H6N5
HM144703|A/duck/Guangxi/1455/2004|H6N5
HM144706|A/duck/Guangxi/585/2005|H6N5
CY109284|A/duck/Guizhou/888/2006|H6N5
AB472027|A/duck/Shiga/69/2006|H6N5
CY098534|A/aquatic-bird/Korea/W69/2005|H6N5
DQ376723|A/duck/Taiwan/WB459/04|H6N5
61
AB270594|A/duck/Hokkaido/1058/2001|H4N5
LC371772|A/duck/Hokkaido/1058/2001|H4N5
AB270600|A/duck/Hokkaido/24/04|H10N5
AB270599|A/duck/Mongolia/149/03|H10N5
LC339661|A/duck/Hokkaido/W259/2005|H2N5
AB472028|A/duck/Tsukuba/255/2005|H8N5
CY079277|A/avian/Japan/8KI0040/2008|H3N5
CY079245|A/avian/Japan/8KI0135/2008|H6N5
LC339605|A/duck/Hokkaido/W132/2004|H12N5
AB270747|A/duck/Hokkaido/66/2001|H12N5
KX977833|A/mallard-duck/Netherlands/13/2007|H6N5
MF147044|A/mallard-duck/Netherlands/4/2011|H12N5
JX679163|A/wild-duck/Korea/SH12-7/2008|H10N5
MH597139|A/ruddy-turnstone/Delaware/1016393/2003|H9N14
MH597627|A/ruddy-turnstone/New-Jersey/1016440/2003|H9N5
MH597120|A/ruddy-turnstone/Delaware/1016391/2003|H9N5
MH597238|A/ruddy-turnstone/Delaware/1016443/2003|H9N5
MH597165|A/ruddy-turnstone/Delaware/1016401/2003|H9N5
CY144466|A/ruddy-turnstone/Delaware/AI03-162/2003|mixedN5
CY144458|A/ruddy-turnstone/Delaware/AI03-165/2003|H9N5
CY102738|A/shorebird/Delaware-Bay/246/2003|H9N5
CY184033|A/mallard/Sweden/3328/2003|H12N5
CY060263|A/mallard/Netherlands/2/1999|H3N5
100
CY149502|A/greylag-goose/Iceland/0948/2011|H6N5
CY149510|A/greylag-goose/Iceland/0953/2011|H6N5
CY149446|A/greylag-goose/Iceland/1459/2011|H6N5
CY149486|A/black-headed-gull/Iceland/1298/2011|H10N5
KR862696|A/Eurasian-wigeon/Netherlands/2/2009|H12N5
MF148102|A/Eurasian-wigeon/Netherlands/2/2009|H12N5
JX566218|A/mallard/Sweden/100103/2009|H12N5
JX566221|A/mallard/Sweden/100127/2009|H12N5
JX566220|A/mallard/Sweden/100117/2009|H12N5
CY183809|A/mallard/Sweden/69773/2007|H10N5
CY183825|A/mallard/Sweden/69792/2007|H10N5
CY184057|A/mallard/Sweden/68529/2007|H12N5
CY184065|A/mallard/Sweden/69794/2007|H12N5
CY184049|A/mallard/Sweden/60069/2007|H12N5
JF789628|A/mallard/Czech-Republic/14924-1/2007|H6N5
GQ415323|A/mallard/Switzerland/WV4060167/2006|H3N5
KY676342|A/turkey/Netherlands/06001571/2006|H6N5
KX978798|A/greater-white-fronted-goose/Netherlands/3/2007|H6N5
KR862694|A/white-fronted-goose/Netherlands/3/2007|H6N5
CY122069|A/mallard/Sweden/30/2002|H2N5
CY184001|A/mallard/Sweden/343/2002|H12N5
CY164570|A/mallard/Sweden/50709/2006|H4N5
CY184041|A/mallard/Sweden/50968/2006|H12N5
CY076978|A/Bewicks-swan/Netherlands/1/2007|H1N5
CY186398|A/mallard/Sweden/80329/2008|H11N5
CY076994|A/black-headed-gull/Netherlands/1/2006|H4N5
KF183615|A/mallard/Finland/10952/2008|H4N5
KX185880|A/duck/Bangladesh/18-C/2013|H1N5
KX185882|A/duck/Bangladesh/33-O/2013|H2N5
AB701297|A/duck/Mongolia/OIE-7457/2011|H3N5
AB560964|A/duck/Hokkaido/111/2009|H1N5
GU220601|A/duck/Yangzhou/013/2008|H6N5
CY184025|A/mallard/Sweden/2213/2003|H12N5
CY060392|A/mallard/Sweden/86/2003|H12N5
Eurasian lineage

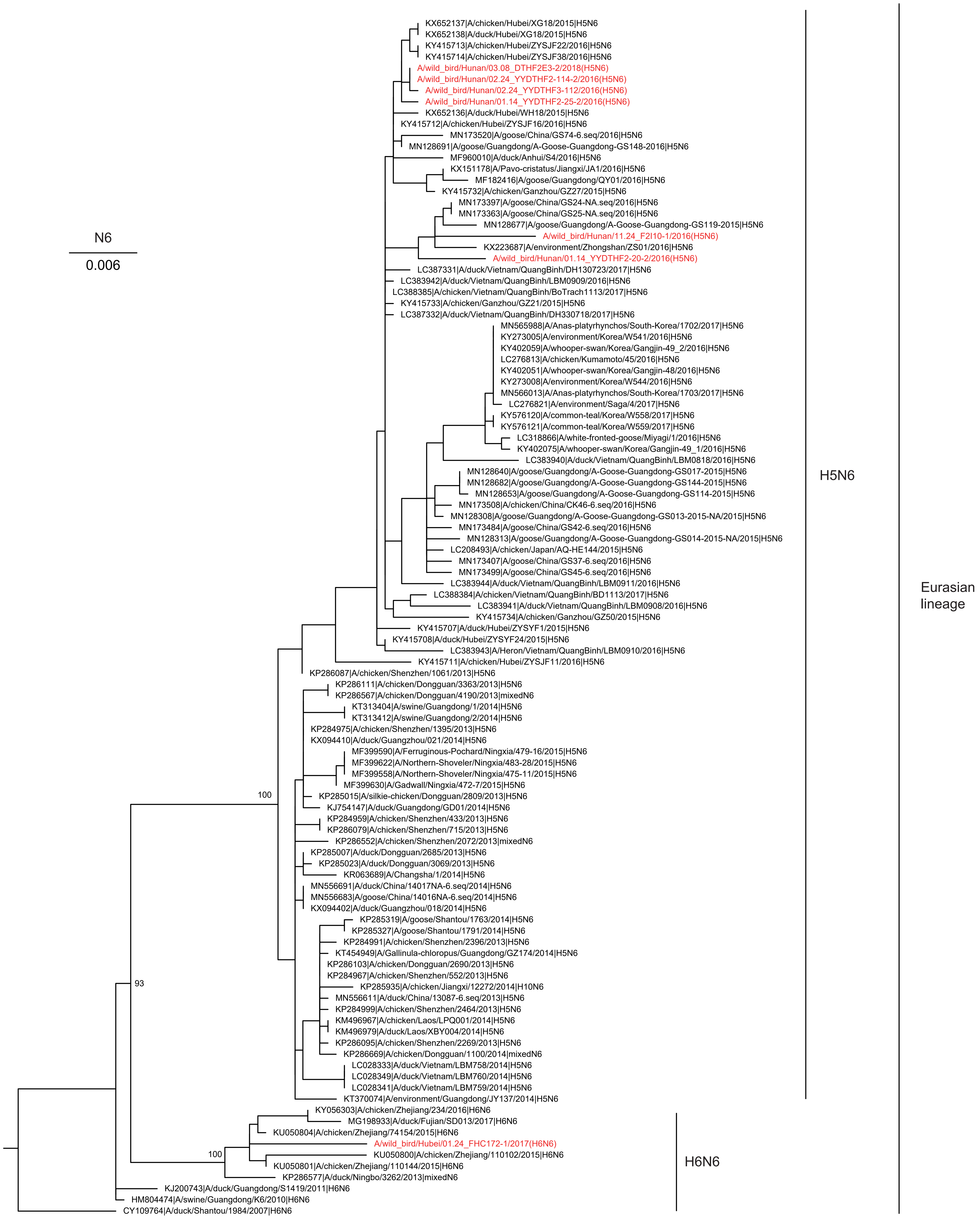

N6
0.006
KX652137|A/chicken/Hubei/XG18/2015|H5N6
KX652138|A/duck/Hubei/XG18/2015|H5N6
KY415713|A/chicken/Hubei/ZYSJF22/2016|H5N6
KY415714|A/chicken/Hubei/ZYSJF38/2016|H5N6
A/wild_bird/Hunan/03.08_DTHF2E3-2/2018(H5N6)
A/wild_bird/Hunan/02.24_YYDTHF2-114-2/2016(H5N6)
A/wild_bird/Hunan/02.24_YYDTHF3-112/2016(H5N6)
A/wild_bird/Hunan/01.14_YYDTHF2-25-2/2016(H5N6)
KX652136|A/duck/Hubei/WH18/2015|H5N6
KY415712|A/chicken/Hubei/ZYSJF16/2016|H5N6
MN173520|A/goose/China/GS74-6.seq/2016|H5N6
MN128691|A/goose/Guangdong/A-Goose-Guangdong-GS148-2016|H5N6
MF960010|A/duck/Anhui/S4/2016|H5N6
KX151178|A/Pavo-cristatus/Jiangxi/JA1/2016|H5N6
MF182416|A/goose/Guangdong/QY01/2016|H5N6
KY415732|A/chicken/Ganzhou/GZ27/2015|H5N6
MN173397|A/goose/China/GS24-NA.seq/2016|H5N6
MN173363|A/goose/China/GS25-NA.seq/2016|H5N6
MN128677|A/goose/Guangdong/A-Goose-Guangdong-GS119-2015|H5N6
A/wild_bird/Hunan/11.24_F2I10-1/2016(H5N6)
KX223687|A/environment/Zhongshan/ZS01/2016|H5N6
A/wild_bird/Hunan/01.14_YYDTHF2-20-2/2016(H5N6)
LC387331|A/duck/Vietnam/QuangBinh/DH130723/2017|H5N6
LC383942|A/duck/Vietnam/QuangBinh/LBM0909/2016|H5N6
LC388385|A/chicken/Vietnam/QuangBinh/BoTrach1113/2017|H5N6
KY415733|A/chicken/Ganzhou/GZ21/2015|H5N6
LC387332|A/duck/Vietnam/QuangBinh/DH330718/2017|H5N6
MN565988|A/Anas-platyrhynchos/South-Korea/1702/2017|H5N6
KY273005|A/environment/Korea/W541/2016|H5N6
KY402059|A/whooper-swan/Korea/Gangjin-49_2/2016|H5N6
LC276813|A/chicken/Kumamoto/45/2016|H5N6
KY402051|A/whooper-swan/Korea/Gangjin-48/2016|H5N6
KY273008|A/environment/Korea/W544/2016|H5N6
MN566013|A/Anas-platyrhynchos/South-Korea/1703/2017|H5N6
LC276821|A/environment/Saga/4/2017|H5N6
KY576120|A/common-teal/Korea/W558/2017|H5N6
KY576121|A/common-teal/Korea/W559/2017|H5N6
LC318866|A/white-fronted-goose/Miyagi/1/2016|H5N6
KY402075|A/whooper-swan/Korea/Gangjin-49_1/2016|H5N6
LC383940|A/duck/Vietnam/QuangBinh/LBM0818/2016|H5N6
MN128640|A/goose/Guangdong/A-Goose-Guangdong-GS017-2015|H5N6
MN128682|A/goose/Guangdong/A-Goose-Guangdong-GS144-2015|H5N6
MN128653|A/goose/Guangdong/A-Goose-Guangdong-GS114-2015|H5N6
MN173508|A/chicken/China/CK46-6.seq/2016|H5N6
MN128308|A/goose/Guangdong/A-Goose-Guangdong-GS013-2015-NA/2015|H5N6
MN173484|A/goose/China/GS42-6.seq/2016|H5N6
MN128313|A/goose/Guangdong/A-Goose-Guangdong-GS014-2015-NA/2015|H5N6
LC208493|A/chicken/Japan/AQ-HE144/2015|H5N6
MN173407|A/goose/China/GS37-6.seq/2016|H5N6
MN173499|A/goose/China/GS45-6.seq/2016|H5N6
LC383944|A/duck/Vietnam/QuangBinh/LBM0911/2016|H5N6
LC388384|A/chicken/Vietnam/QuangBinh/BD1113/2017|H5N6
LC383941|A/duck/Vietnam/QuangBinh/LBM0908/2016|H5N6
KY415734|A/chicken/Ganzhou/GZ50/2015|H5N6
KY415707|A/duck/Hubei/ZYSYF1/2015|H5N6
KY415708|A/duck/Hubei/ZYSYF24/2015|H5N6
LC383943|A/Heron/Vietnam/QuangBinh/LBM0910/2016|H5N6
KY415711|A/chicken/Hubei/ZYSJF11/2016|H5N6
KP286087|A/chicken/Shenzhen/1061/2013|H5N6
KP286111|A/chicken/Dongguan/3363/2013|H5N6
KP286567|A/chicken/Dongguan/4190/2013|mixedN6
KT313404|A/swine/Guangdong/1/2014|H5N6
KT313412|A/swine/Guangdong/2/2014|H5N6
KP284975|A/chicken/Shenzhen/1395/2013|H5N6
KX094410|A/duck/Guangzhou/021/2014|H5N6
MF399590|A/Ferruginous-Pochard/Ningxia/479-16/2015|H5N6
MF399622|A/Northern-Shoveler/Ningxia/483-28/2015|H5N6
MF399558|A/Northern-Shoveler/Ningxia/475-11/2015|H5N6
MF399630|A/Gadwall/Ningxia/472-7/2015|H5N6
100
KP285015|A/silkie-chicken/Dongguan/2809/2013|H5N6
KJ754147|A/duck/Guangdong/GD01/2014|H5N6
KP284959|A/chicken/Shenzhen/433/2013|H5N6
KP286079|A/chicken/Shenzhen/715/2013|H5N6
KP286552|A/chicken/Shenzhen/2072/2013|mixedN6
KP285007|A/duck/Dongguan/2685/2013|H5N6
KP285023|A/duck/Dongguan/3069/2013|H5N6
KR063689|A/Changsha/1/2014|H5N6
MN556691|A/duck/China/14017NA-6.seq/2014|H5N6
MN556683|A/goose/China/14016NA-6.seq/2014|H5N6
KX094402|A/duck/Guangzhou/018/2014|H5N6
KP285319|A/goose/Shantou/1763/2014|H5N6
KP285327|A/goose/Shantou/1791/2014|H5N6
KP284991|A/chicken/Shenzhen/2396/2013|H5N6
KT454949|A/Gallinula-chloropus/Guangdong/GZ174/2014|H5N6
KP286103|A/chicken/Dongguan/2690/2013|H5N6
KP284967|A/chicken/Shenzhen/552/2013|H5N6
93
KP285935|A/chicken/Jiangxi/12272/2014|H10N6
MN556611|A/duck/China/13087-6.seq/2013|H5N6
KP284999|A/chicken/Shenzhen/2464/2013|H5N6
KM496967|A/chicken/Laos/LPQ001/2014|H5N6
KM496979|A/duck/Laos/XBY004/2014|H5N6
KP286095|A/chicken/Shenzhen/2269/2013|H5N6
KP286669|A/chicken/Dongguan/1100/2014|mixedN6
LC028333|A/duck/Vietnam/LBM758/2014|H5N6
LC028349|A/duck/Vietnam/LBM760/2014|H5N6
LC028341|A/duck/Vietnam/LBM759/2014|H5N6
KT370074|A/environment/Guangdong/JY137/2014|H5N6
KY056303|A/chicken/Zhejiang/234/2016|H6N6
MG198933|A/duck/Fujian/SD013/2017|H6N6
KU050804|A/chicken/Zhejiang/74154/2015|H6N6
A/wild_bird/Hubei/01.24_FHC172-1/2017(H6N6)
100
KU050800|A/chicken/Zhejiang/110102/2015|H6N6
KU050801|A/chicken/Zhejiang/110144/2015|H6N6
KP286577|A/duck/Ningbo/3262/2013|mixedN6
KJ200743|A/duck/Guangdong/S1419/2011|H6N6
HM804474|A/swine/Guangdong/K6/2010|H6N6
CY109764|A/duck/Shantou/1984/2007|H6N6
H5N6
H6N6
Eurasian lineage

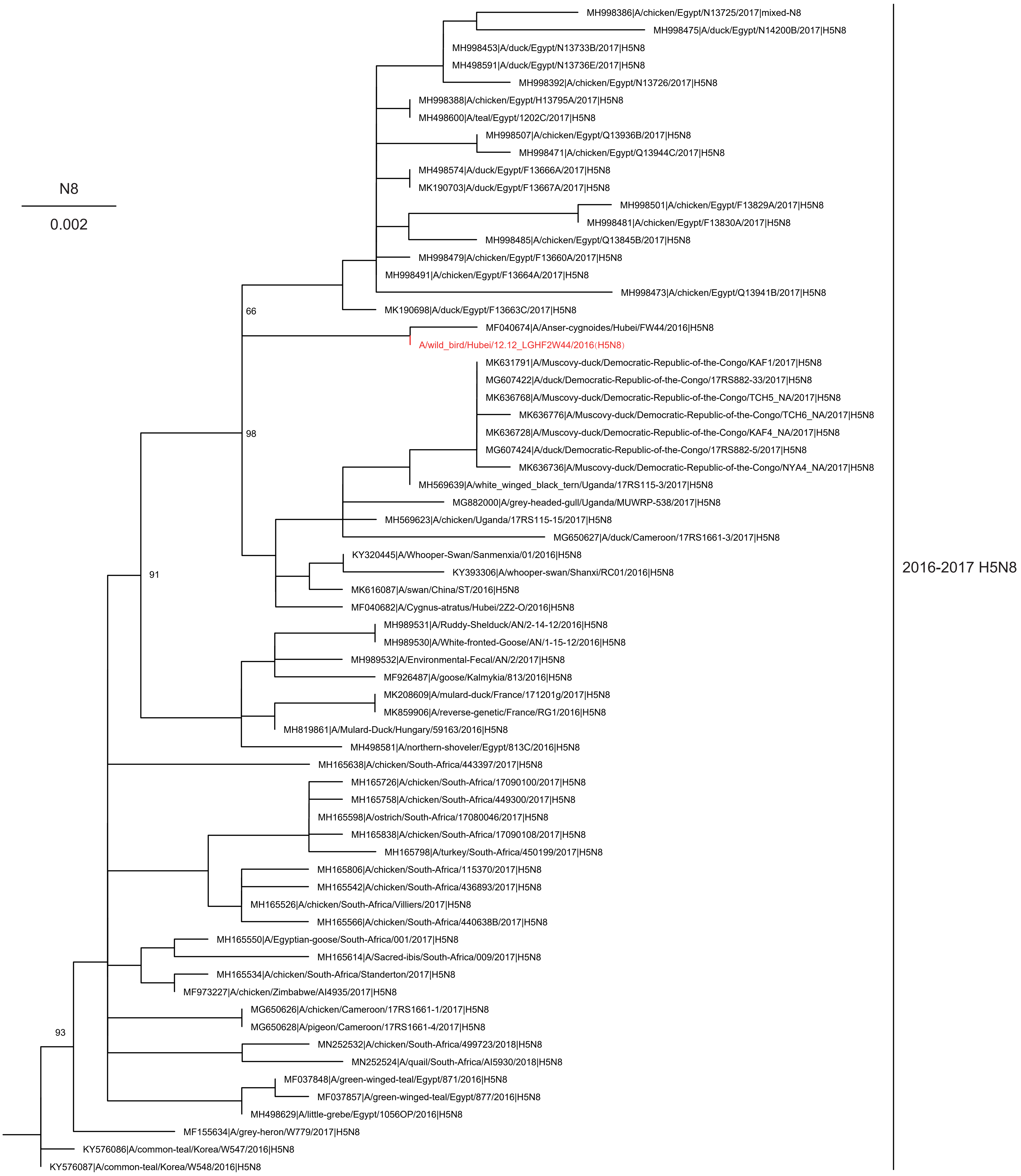

N8
0.002
MH998386|A/chicken/Egypt/N13725/2017|mixed-N8
MH998475|A/duck/Egypt/N14200B/2017|H5N8
MH998453|A/duck/Egypt/N13733B/2017|H5N8
MH498591|A/duck/Egypt/N13736E/2017|H5N8
MH998392|A/chicken/Egypt/N13726/2017|H5N8
MH998388|A/chicken/Egypt/H13795A/2017|H5N8
MH498600|A/teal/Egypt/1202C/2017|H5N8
MH998507|A/chicken/Egypt/Q13936B/2017|H5N8
MH998471|A/chicken/Egypt/Q13944C/2017|H5N8
MH498574|A/duck/Egypt/F13666A/2017|H5N8
MK190703|A/duck/Egypt/F13667A/2017|H5N8
MH998501|A/chicken/Egypt/F13829A/2017|H5N8
MH998481|A/chicken/Egypt/F13830A/2017|H5N8
MH998485|A/chicken/Egypt/Q13845B/2017|H5N8
MH998479|A/chicken/Egypt/F13660A/2017|H5N8
MH998491|A/chicken/Egypt/F13664A/2017|H5N8
MH998473|A/chicken/Egypt/Q13941B/2017|H5N8
MK190698|A/duck/Egypt/F13663C/2017|H5N8
66
MF040674|A/Anser-cygnoides/Hubei/FW44/2016|H5N8
A/wild_bird/Hubei/12.12_LGHF2W44/2016(H5N8)
MK631791|A/Muscovy-duck/Democratic-Republic-of-the-Congo/KAF1/2017|H5N8
MG607422|A/duck/Democratic-Republic-of-the-Congo/17RS882-33/2017|H5N8
MK636768|A/Muscovy-duck/Democratic-Republic-of-the-Congo/TCH5_NA/2017|H5N8
MK636776|A/Muscovy-duck/Democratic-Republic-of-the-Congo/TCH6_NA/2017|H5N8
98
MK636728|A/Muscovy-duck/Democratic-Republic-of-the-Congo/KAF4_NA/2017|H5N8
MG607424|A/duck/Democratic-Republic-of-the-Congo/17RS882-5/2017|H5N8
MK636736|A/Muscovy-duck/Democratic-Republic-of-the-Congo/NYA4_NA/2017|H5N8
MH569639|A/white_winged_black_tern/Uganda/17RS115-3/2017|H5N8
MG882000|A/grey-headed-gull/Uganda/MUWRP-538/2017|H5N8
MH569623|A/chicken/Uganda/17RS115-15/2017|H5N8
MG650627|A/duck/Cameroon/17RS1661-3/2017|H5N8
KY320445|A/Whooper-Swan/Sanmenxia/01/2016|H5N8
91
KY393306|A/whooper-swan/Shanxi/RC01/2016|H5N8
2016-2017 H5N8
MK616087|A/swan/China/ST/2016|H5N8
MF040682|A/Cygnus-atratus/Hubei/2Z2-O/2016|H5N8
MH989531|A/Ruddy-Shelduck/AN/2-14-12/2016|H5N8
MH989530|A/White-fronted-Goose/AN/1-15-12/2016|H5N8
MH989532|A/Environmental-Fecal/AN/2/2017|H5N8
MF926487|A/goose/Kalmykia/813/2016|H5N8
MK208609|A/mulard-duck/France/171201g/2017|H5N8
MK859906|A/reverse-genetic/France/RG1/2016|H5N8
MH819861|A/Mulard-Duck/Hungary/59163/2016|H5N8
MH498581|A/northern-shoveler/Egypt/813C/2016|H5N8
MH165638|A/chicken/South-Africa/443397/2017|H5N8
MH165726|A/chicken/South-Africa/17090100/2017|H5N8
MH165758|A/chicken/South-Africa/449300/2017|H5N8
MH165598|A/ostrich/South-Africa/17080046/2017|H5N8
MH165838|A/chicken/South-Africa/17090108/2017|H5N8
MH165798|A/turkey/South-Africa/450199/2017|H5N8
MH165806|A/chicken/South-Africa/115370/2017|H5N8
MH165542|A/chicken/South-Africa/436893/2017|H5N8
MH165526|A/chicken/South-Africa/Villiers/2017|H5N8
MH165566|A/chicken/South-Africa/440638B/2017|H5N8
MH165550|A/Egyptian-goose/South-Africa/001/2017|H5N8
MH165614|A/Sacred-ibis/South-Africa/009/2017|H5N8
MH165534|A/chicken/South-Africa/Standerton/2017|H5N8
MF973227|A/chicken/Zimbabwe/AI4935/2017|H5N8
MG650626|A/chicken/Cameroon/17RS1661-1/2017|H5N8
93
MG650628|A/pigeon/Cameroon/17RS1661-4/2017|H5N8
MN252532|A/chicken/South-Africa/499723/2018|H5N8
MN252524|A/quail/South-Africa/AI5930/2018|H5N8
MF037848|A/green-winged-teal/Egypt/871/2016|H5N8
MF037857|A/green-winged-teal/Egypt/877/2016|H5N8
MH498629|A/little-grebe/Egypt/1056OP/2016|H5N8
MF155634|A/grey-heron/W779/2017|H5N8
KY576086|A/common-teal/Korea/W547/2016|H5N8
KY576087|A/common-teal/Korea/W548/2016|H5N8

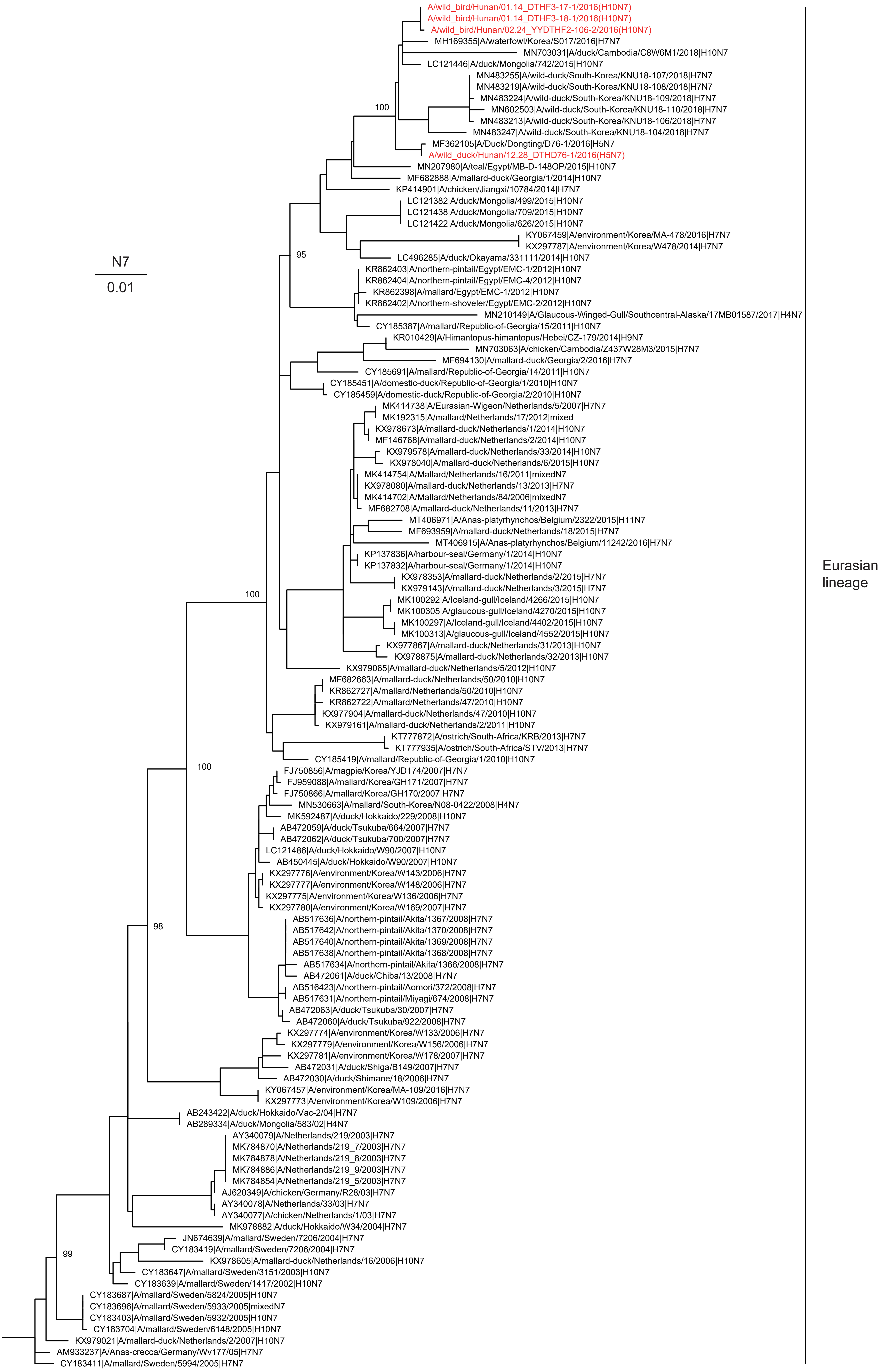

A/wild_bird/Hunan/01.14_DTHF3-17-1/2016(H10N7)
A/wild_bird/Hunan/01.14_DTHF3-18-1/2016(H10N7)
A/wild_bird/Hunan/02.24_YYDTHF2-106-2/2016(H10N7)
MH169355|A/waterfowl/Korea/S017/2016|H7N7
MN703031|A/duck/Cambodia/C8W6M1/2018|H10N7
LC121446|A/duck/Mongolia/742/2015|H10N7
MN483255|A/wild-duck/South-Korea/KNU18-107/2018|H7N7
MN483219|A/wild-duck/South-Korea/KNU18-108/2018|H7N7
MN483224|A/wild-duck/South-Korea/KNU18-109/2018|H7N7
MN602503|A/wild-duck/South-Korea/KNU18-110/2018|H7N7
MN483213|A/wild-duck/South-Korea/KNU18-106/2018|H7N7
MN483247|A/wild-duck/South-Korea/KNU18-104/2018|H7N7
100
MF362105|A/Duck/Dongting/D76-1/2016|H5N7
A/wild_duck/Hunan/12.28_DTHD76-1/2016(H5N7)
MN207980|A/teal/Egypt/MB-D-148OP/2015|H10N7
MF682888|A/mallard-duck/Georgia/1/2014|H10N7
KP414901|A/chicken/Jiangxi/10784/2014|H7N7
LC121382|A/duck/Mongolia/499/2015|H10N7
LC121438|A/duck/Mongolia/709/2015|H10N7
LC121422|A/duck/Mongolia/626/2015|H10N7
KY067459|A/environment/Korea/MA-478/2016|H7N7
KX297787|A/environment/Korea/W478/2014|H7N7
95
LC496285|A/duck/Okayama/331111/2014|H10N7
N7
0.01
KR862403|A/northern-pintail/Egypt/EMC-1/2012|H10N7
KR862404|A/northern-pintail/Egypt/EMC-4/2012|H10N7
KR862398|A/mallard/Egypt/EMC-1/2012|H10N7
KR862402|A/northern-shoveler/Egypt/EMC-2/2012|H10N7
MN210149|A/Glaucous-Winged-Gull/Southcentral-Alaska/17MB01587/2017|H4N7
CY185387|A/mallard/Republic-of-Georgia/15/2011|H10N7
KR010429|A/Himantopus-himantopus/Hebei/CZ-179/2014|H9N7
MN703063|A/chicken/Cambodia/Z437W28M3/2015|H7N7
MF694130|A/mallard-duck/Georgia/2/2016|H7N7
CY185691|A/mallard/Republic-of-Georgia/14/2011|H10N7
CY185451|A/domestic-duck/Republic-of-Georgia/1/2010|H10N7
CY185459|A/domestic-duck/Republic-of-Georgia/2/2010|H10N7
MK414738|A/Eurasian-Wigeon/Netherlands/5/2007|H7N7
MK192315|A/mallard/Netherlands/17/2012|mixed
KX978673|A/mallard-duck/Netherlands/1/2014|H10N7
MF146768|A/mallard-duck/Netherlands/2/2014|H10N7
KX979578|A/mallard-duck/Netherlands/33/2014|H10N7
KX978040|A/mallard-duck/Netherlands/6/2015|H10N7
MK414754|A/Mallard/Netherlands/16/2011|mixedN7
KX978080|A/mallard-duck/Netherlands/13/2013|H7N7
MK414702|A/Mallard/Netherlands/84/2006|mixedN7
MF682708|A/mallard-duck/Netherlands/11/2013|H7N7
MT406971|A/Anas-platyrhynchos/Belgium/2322/2015|H11N7
MF693959|A/mallard-duck/Netherlands/18/2015|H7N7
MT406915|A/Anas-platyrhynchos/Belgium/11242/2016|H7N7
KP137836|A/harbour-seal/Germany/1/2014|H10N7
KP137832|A/harbour-seal/Germany/1/2014|H10N7
KX978353|A/mallard-duck/Netherlands/2/2015|H7N7
KX979143|A/mallard-duck/Netherlands/3/2015|H7N7
Eurasian lineage
100
MK100292|A/Iceland-gull/Iceland/4266/2015|H10N7
MK100305|A/glaucous-gull/Iceland/4270/2015|H10N7
MK100297|A/Iceland-gull/Iceland/4402/2015|H10N7
MK100313|A/glaucous-gull/Iceland/4552/2015|H10N7
KX977867|A/mallard-duck/Netherlands/31/2013|H10N7
KX978875|A/mallard-duck/Netherlands/32/2013|H10N7
KX979065|A/mallard-duck/Netherlands/5/2012|H10N7
MF682663|A/mallard-duck/Netherlands/50/2010|H10N7
KR862727|A/mallard/Netherlands/50/2010|H10N7
KR862722|A/mallard/Netherlands/47/2010|H10N7
KX977904|A/mallard-duck/Netherlands/47/2010|H10N7
KX979161|A/mallard-duck/Netherlands/2/2011|H10N7
KT777872|A/ostrich/South-Africa/KRB/2013|H7N7
KT777935|A/ostrich/South-Africa/STV/2013|H7N7
CY185419|A/mallard/Republic-of-Georgia/1/2010|H10N7
100
FJ750856|A/magpie/Korea/YJD174/2007|H7N7
FJ959088|A/mallard/Korea/GH171/2007|H7N7
FJ750866|A/mallard/Korea/GH170/2007|H7N7
MN530663|A/mallard/South-Korea/N08-0422/2008|H4N7
MK592487|A/duck/Hokkaido/229/2008|H10N7
AB472059|A/duck/Tsukuba/664/2007|H7N7
AB472062|A/duck/Tsukuba/700/2007|H7N7
LC121486|A/duck/Hokkaido/W90/2007|H10N7
AB450445|A/duck/Hokkaido/W90/2007|H10N7
KX297776|A/environment/Korea/W143/2006|H7N7
KX297777|A/environment/Korea/W148/2006|H7N7
KX297775|A/environment/Korea/W136/2006|H7N7
KX297780|A/environment/Korea/W169/2007|H7N7
98
AB517636|A/northern-pintail/Akita/1367/2008|H7N7
AB517642|A/northern-pintail/Akita/1370/2008|H7N7
AB517640|A/northern-pintail/Akita/1369/2008|H7N7
AB517638|A/northern-pintail/Akita/1368/2008|H7N7
AB517634|A/northern-pintail/Akita/1366/2008|H7N7
AB472061|A/duck/Chiba/13/2008|H7N7
AB516423|A/northern-pintail/Aomori/372/2008|H7N7
AB517631|A/northern-pintail/Miyagi/674/2008|H7N7
AB472063|A/duck/Tsukuba/30/2007|H7N7
AB472060|A/duck/Tsukuba/922/2008|H7N7
KX297774|A/environment/Korea/W133/2006|H7N7
KX297779|A/environment/Korea/W156/2006|H7N7
KX297781|A/environment/Korea/W178/2007|H7N7
AB472031|A/duck/Shiga/B149/2007|H7N7
AB472030|A/duck/Shimane/18/2006|H7N7
KY067457|A/environment/Korea/MA-109/2016|H7N7
KX297773|A/environment/Korea/W109/2006|H7N7
AB243422|A/duck/Hokkaido/Vac-2/04|H7N7
AB289334|A/duck/Mongolia/583/02|H4N7
AY340079|A/Netherlands/219/2003|H7N7
MK784870|A/Netherlands/219_7/2003|H7N7
MK784878|A/Netherlands/219_8/2003|H7N7
MK784886|A/Netherlands/219_9/2003|H7N7
MK784854|A/Netherlands/219_5/2003|H7N7
AJ620349|A/chicken/Germany/R28/03|H7N7
AY340078|A/Netherlands/33/03|H7N7
AY340077|A/chicken/Netherlands/1/03|H7N7
MK978882|A/duck/Hokkaido/W34/2004|H7N7
JN674639|A/mallard/Sweden/7206/2004|H7N7
CY183419|A/mallard/Sweden/7206/2004|H7N7
99
KX978605|A/mallard-duck/Netherlands/16/2006|H10N7
CY183647|A/mallard/Sweden/3151/2003|H10N7
CY183639|A/mallard/Sweden/1417/2002|H10N7
CY183687|A/mallard/Sweden/5824/2005|H10N7
CY183696|A/mallard/Sweden/5933/2005|mixedN7
CY183403|A/mallard/Sweden/5932/2005|H10N7
CY183704|A/mallard/Sweden/6148/2005|H10N7
KX979021|A/mallard-duck/Netherlands/2/2007|H10N7
AM933237|A/Anas-crecca/Germany/Wv177/05|H7N7
CY183411|A/mallard/Sweden/5994/2005|H7N7

Fig S1. Phylogeny of HA and NA genes of 89AIVs. Maximum likelihood trees were generated by IQ-tree software under the GTR-GAMMA model with 1000 bootstrap replicates. Only CDS regions of gene sequences were used for the phylogenetic analyses. Phylogenetic tree of H1 was rooted to A/mallard-duck/Alberta/109/1977(H1N1); Phylogenetic tree of H5 was rooted to A/Sichuan/26221/2014(H5N6); Phylogenetic tree of H6 was rooted to A/ruddy-turnstone/New-Jersey/AI10-652/2010(H6N1); Phylogenetic tree of H9 was rooted to A/northern-shoveler/Missouri/298/2009(H9N2); Phylogenetic tree of H10 was rooted to A/Aquatic-bird/South-Korea/SW1/2018(H10N1); Phylogenetic tree of N1 was rooted to A/Anas-platyrhynchos/Belgium/1837-H101620/2018(H10N1); Phylogenetic tree of N2 was rooted to A/Alopochen-aegyptiacus/Belgium/3237-B6663/2017(H1N2); Phylogenetic tree of N5 was rooted to A/mallard/Sweden/86/2003(H12N5); Phylogenetic tree of N7 was rooted to A/mallard/Sweden/5994/2005(H7N7); Phylogenetic tree of N8 was rooted to A/common-teal/Korea/W548/2016(H5N8). Sequences reported in this study are colored with red. The WHO classification was used to describe the subclades of clade 2.3.4.4 HPAIVs, and reference sequences are colored with purple.

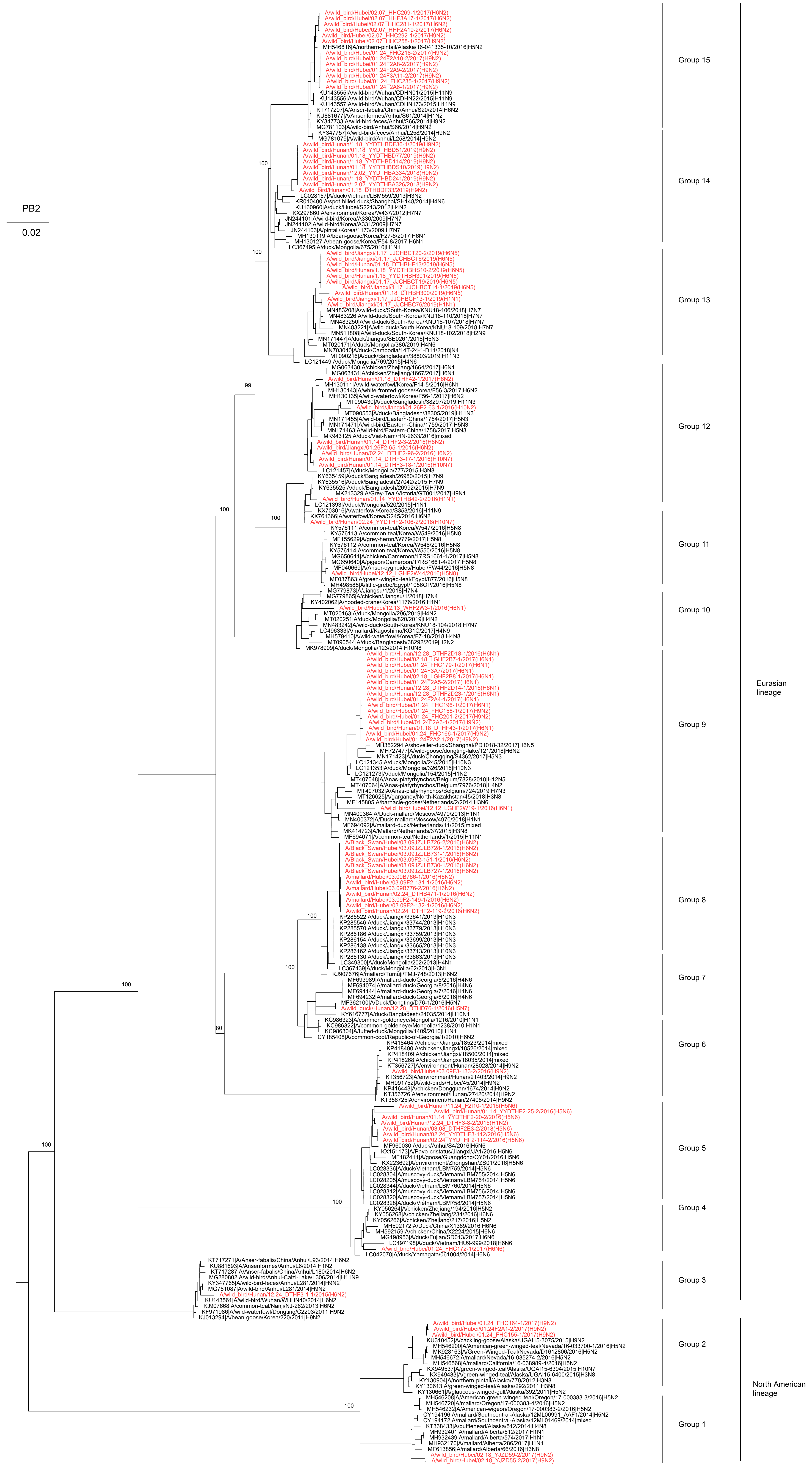
PB2
0.02
Group 15
Group 14
Group 13
Group 12
Group 11
Group 10
Group 9
Group 8
Group 7
Group 6
Group 5
Group 4
Group 3
Group 2
Group 1
Eurasian lineage
North American lineage
100
100
99
100
100
100
100
100
80
100
100
100
100

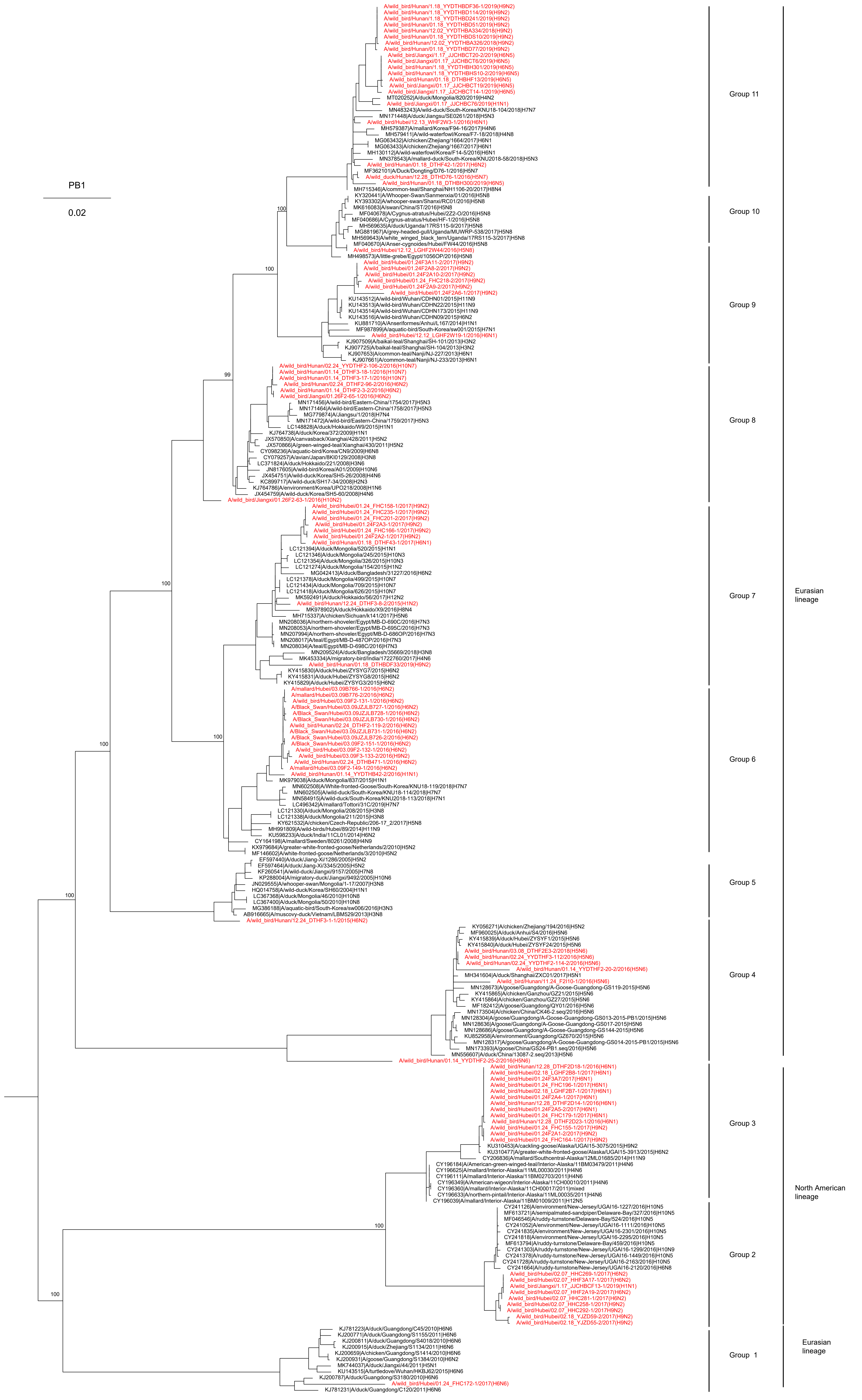
PB1
0.02
Group 11
Group 10
Group 9
Group 8
Group 7
Group 6
Group 5
Group 4
Group 3
Group 2
Group 1
Eurasian lineage
North American lineage
Eurasian lineage

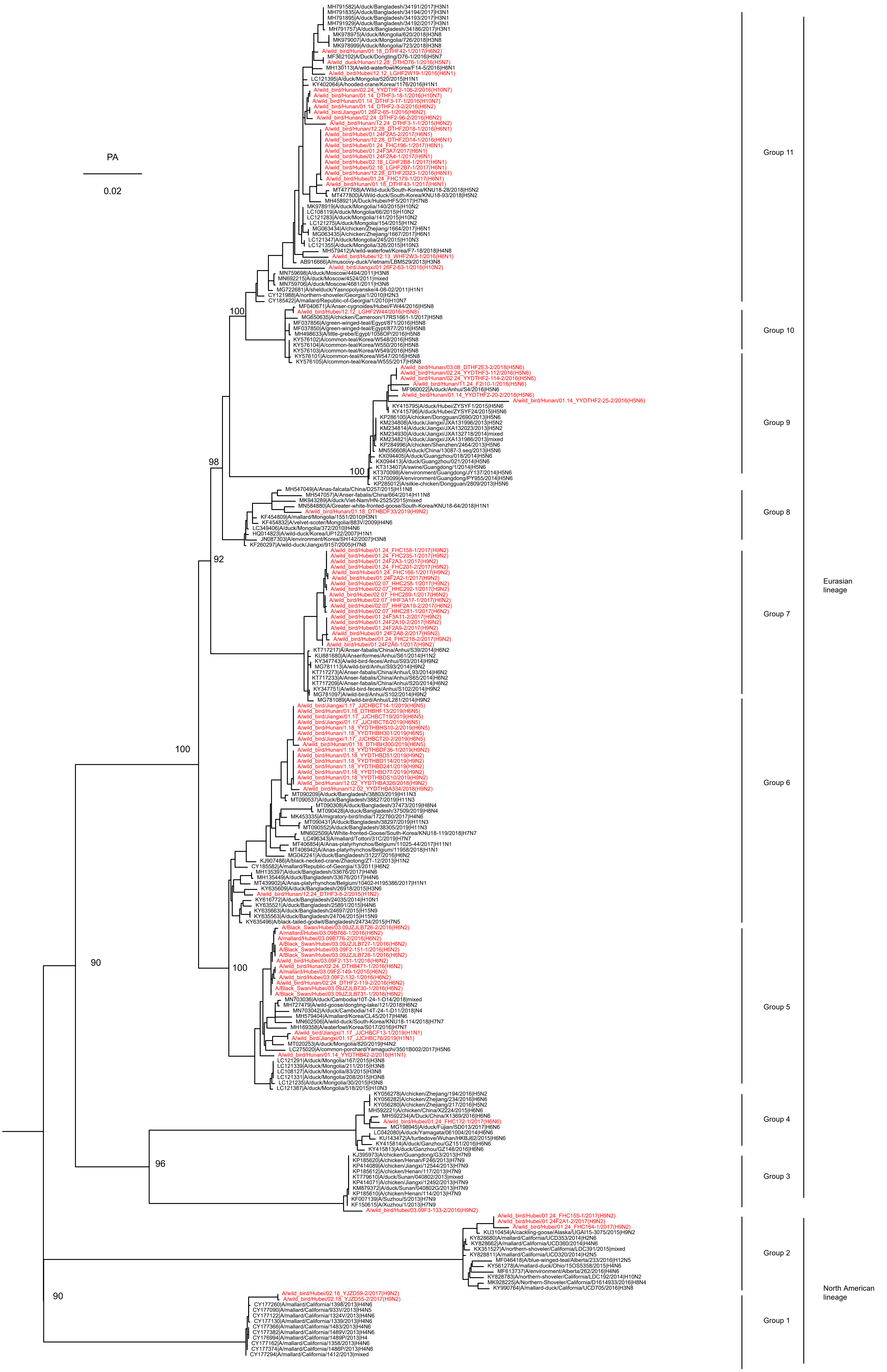
PA
0.02
100
98
92
100
90
90
96
100
100
Group 11
Group 10
Group 9
Group 8
Group 7
Group 6
Group 5
Group 4
Group 3
Group 2
Group 1
Eurasian lineage
North American lineage

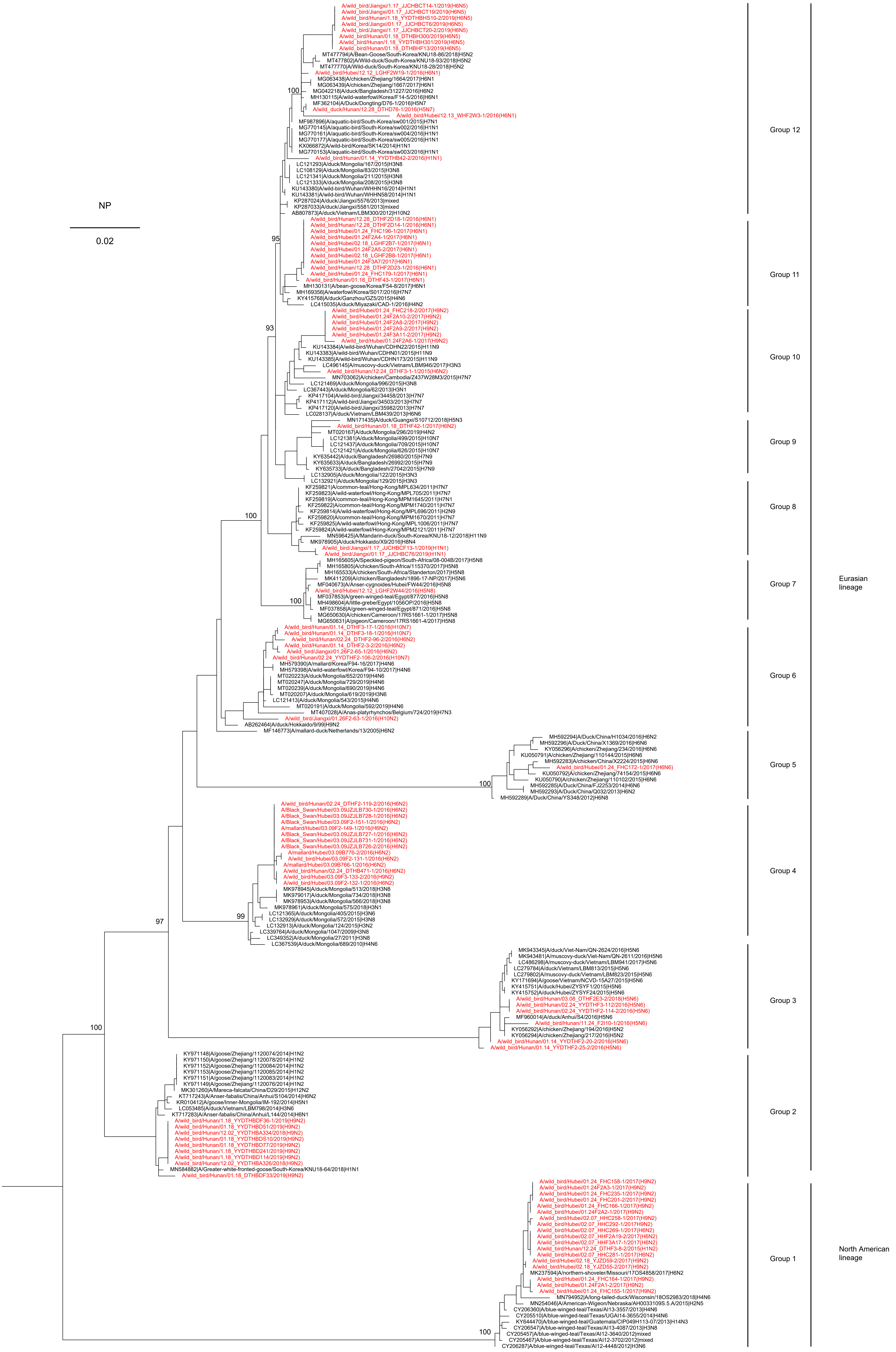

NP
0.02
Group 12
Group 11
Group 10
Group 9
Group 8
Group 7
Group 6
Group 5
Group 4
Group 3
Group 2
Group 1
Eurasian lineage
North American lineage
100
95
93
100
100
100
99
97
100
100

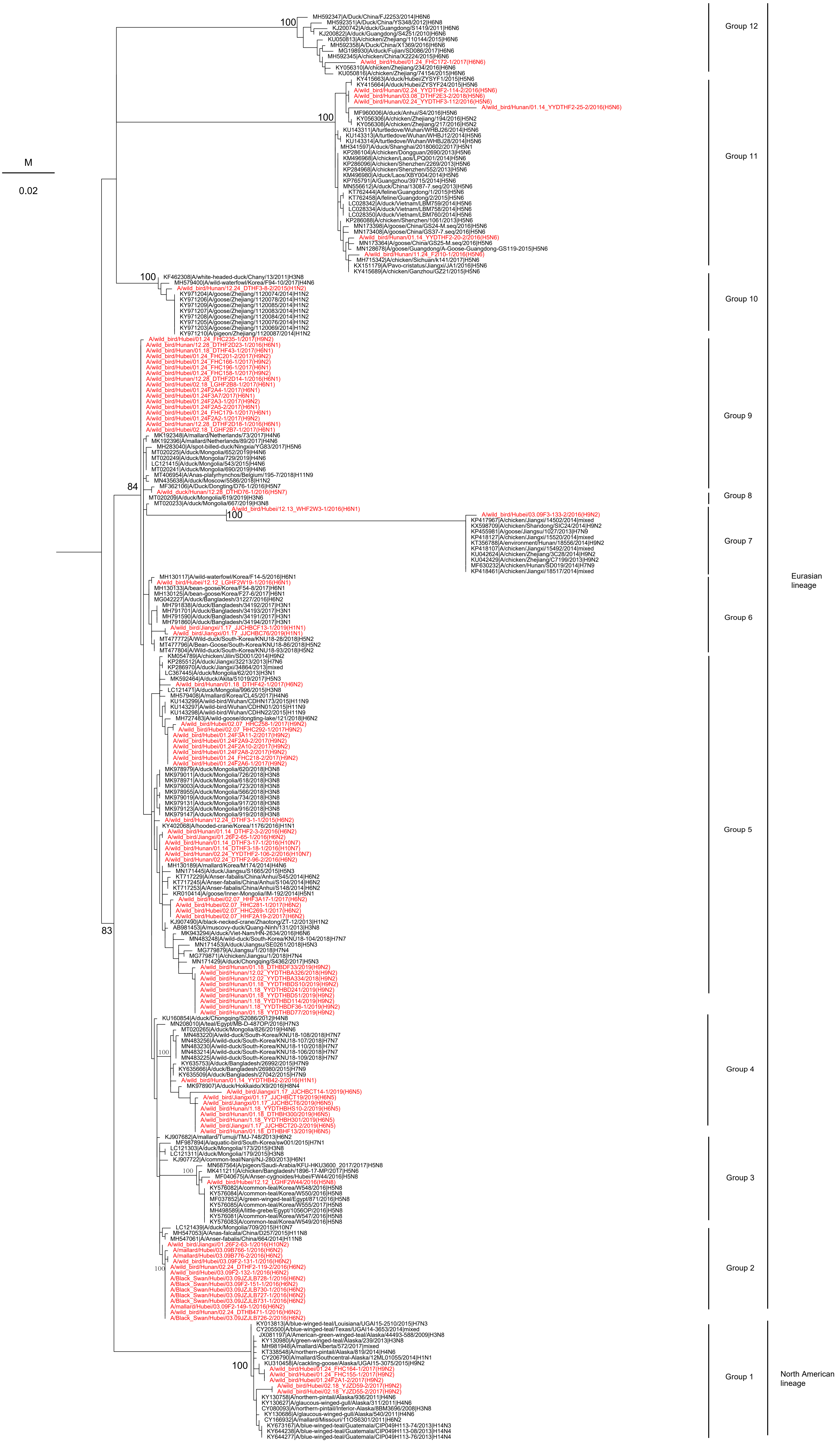
M
0.02
100
100
100
84
100
83
100
100
100
100
Group 12
Group 11
Group 10
Group 9
Group 8
Group 7
Group 6
Group 5
Group 4
Group 3
Group 2
Group 1
Eurasian lineage
North American lineage

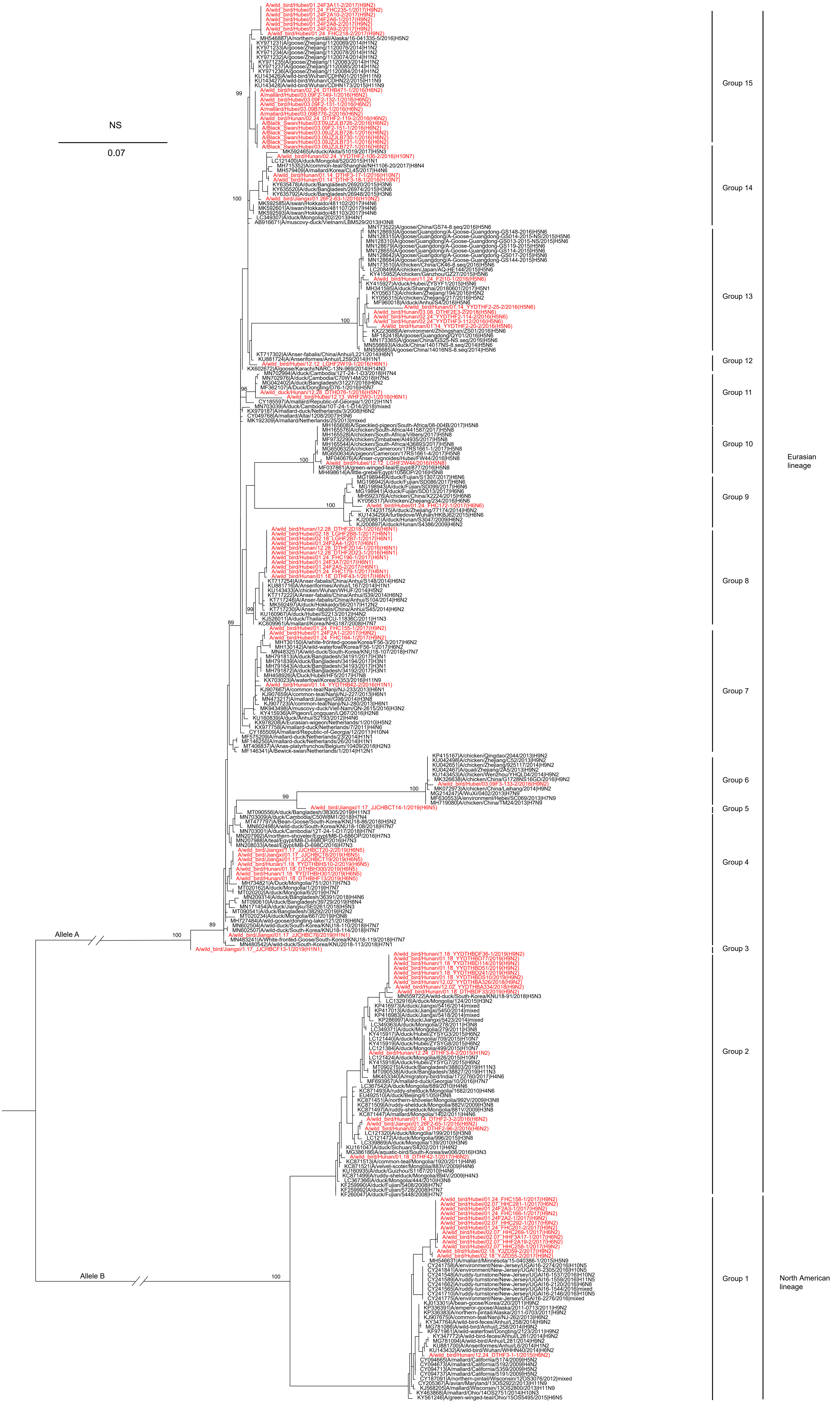
NS
0.07
Group 15
Group 14
Group 13
Group 12
Group 11
Group 10
Group 9
Group 8
Group 7
Group 6
Group 5
Group 4
Group 3
Group 2
Group 1
Eurasian lineage
North American lineage
Allele A
Allele B

Fig S2. Phylogenetic trees of internal genes of 89 AIVs. Maximum likelihood trees of PB2 (n=254), PB1 (n=228), PA (n=244), NP (n=221), MP (n=252), and NS (n=296) genes were generated by IQ-tree software under the GTR-GAMMA model. Only CDS regions of gene sequences were used for the phylogenetic analyses. All trees are midpoint rooted and bootstrap values (%) of 1,000 pseudo-replicates are shown at relevant nodes. Sequences reported in this study are marked in red. The phylogenetic classification of each internal gene tree was defined by strong bootstrap support (>80%) and similarity of different clades (< 98%).

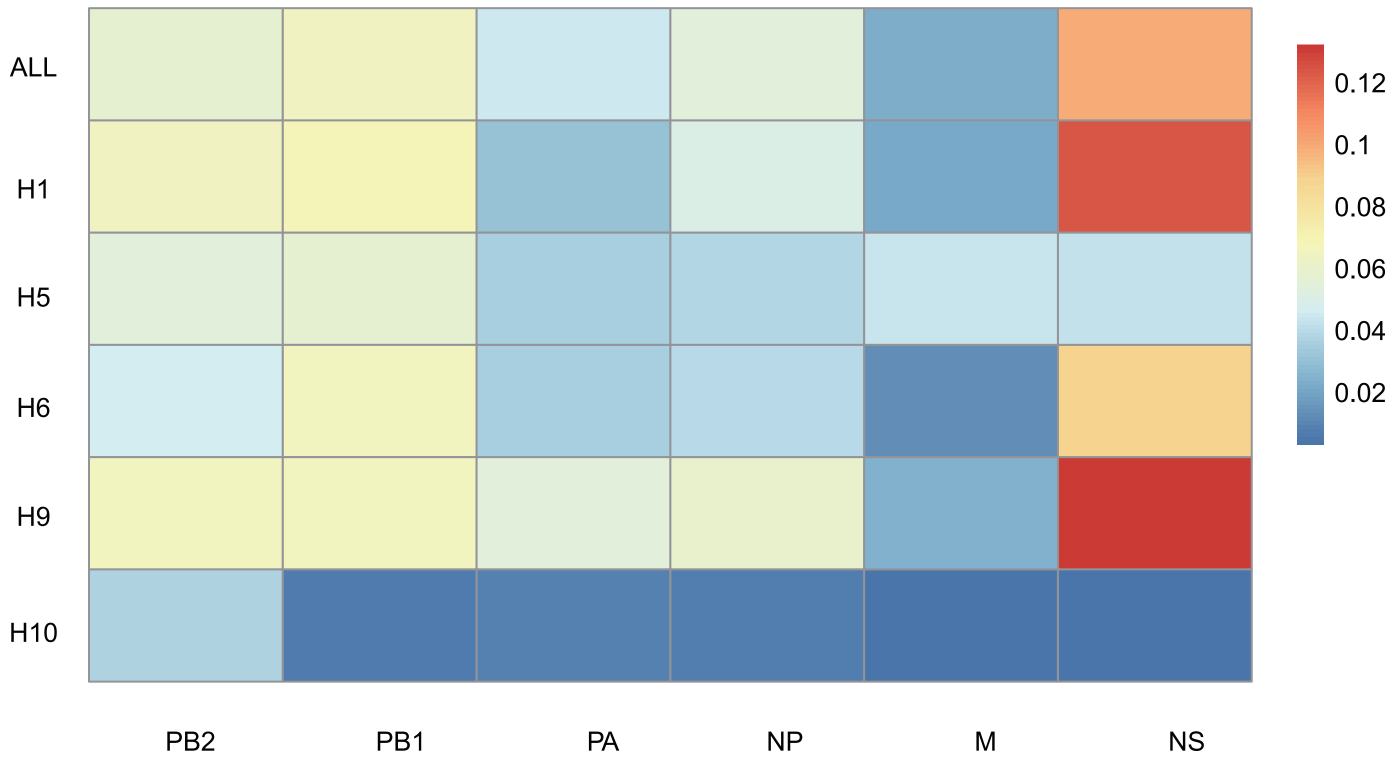

Fig S3. Genetic diversity of avian influenza viruses. Comparison of nucleotide diversity among HA subtypes. Per-site nucleotide sequence diversity, defined as the average number of nucleotide differences per site between two sequences in all possible pairs. Higher diversity in accordance with higher value.

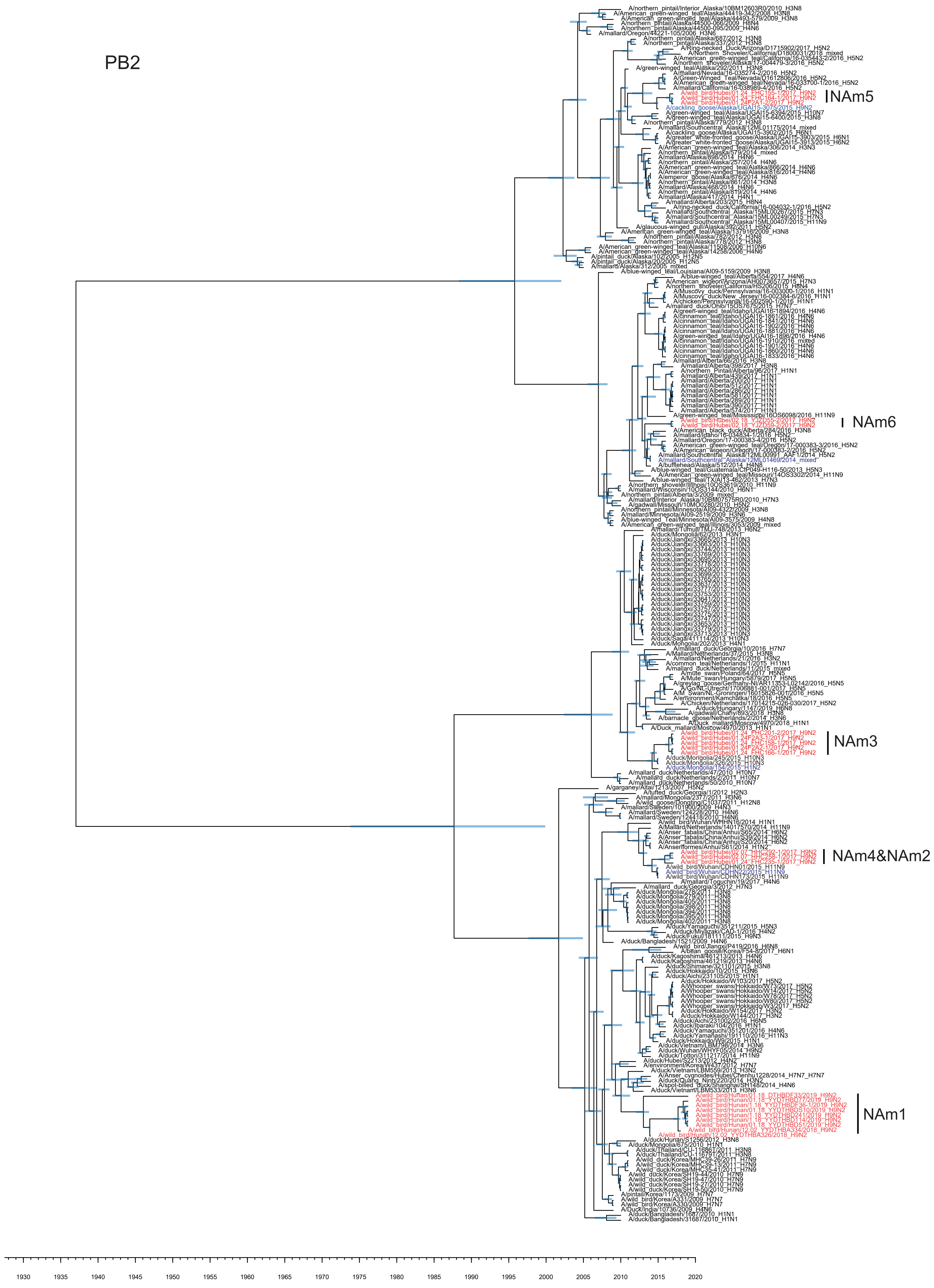
PB2
NAm5
NAm6
NAm3
NAm4&NAm2
NAm1
1930
1935
1940
1945
1950
1955
1960
1965
1970
1975
1980
1985
1990
1995
2000
2005
2010
2015
2020

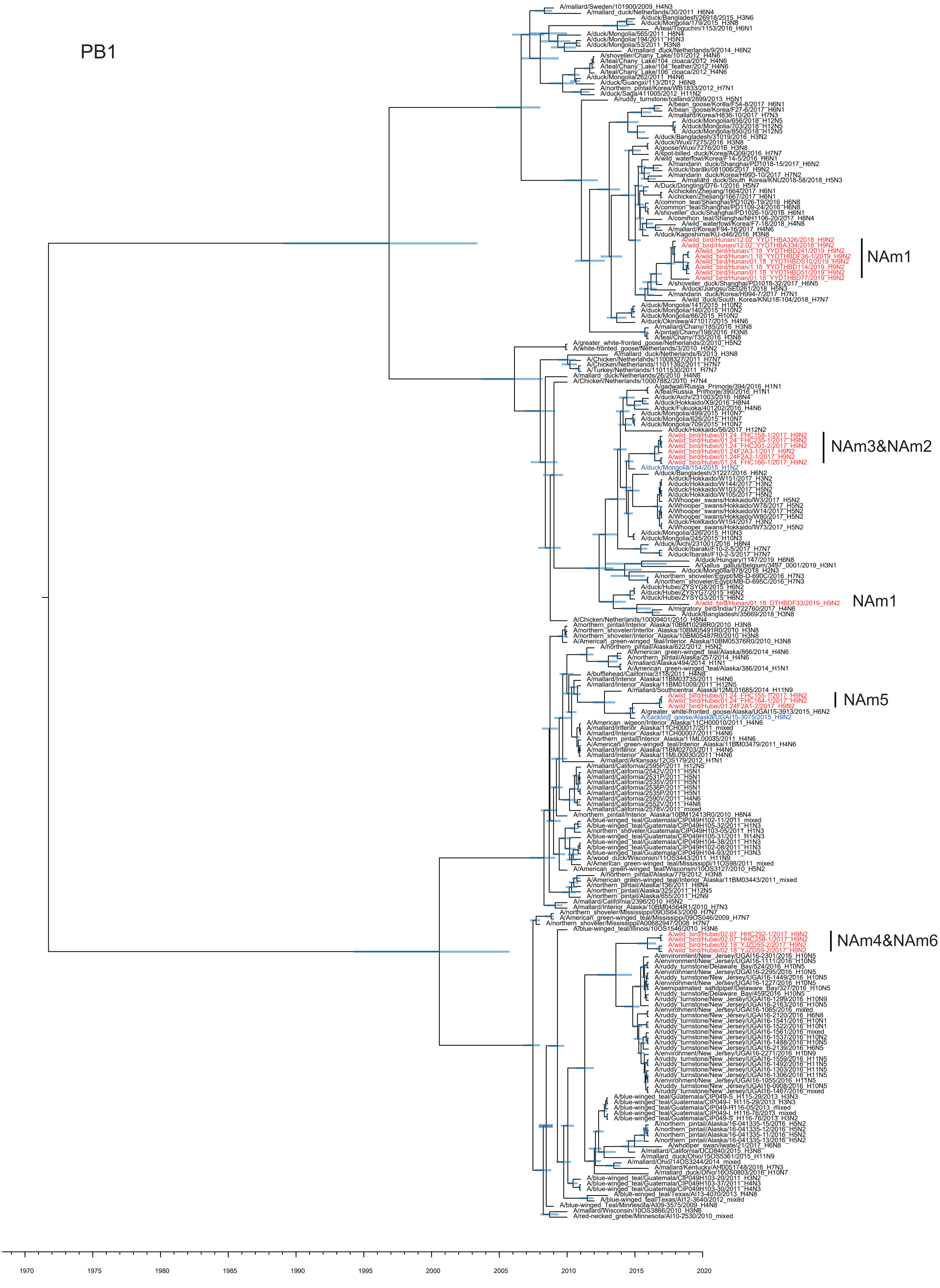
PB1
NAm1
NAm3&NAm2
NAm1
NAm5
NAm4&NAm6
1970
1975
1980
1985
1990
1995
2000
2005
2010
2015
2020

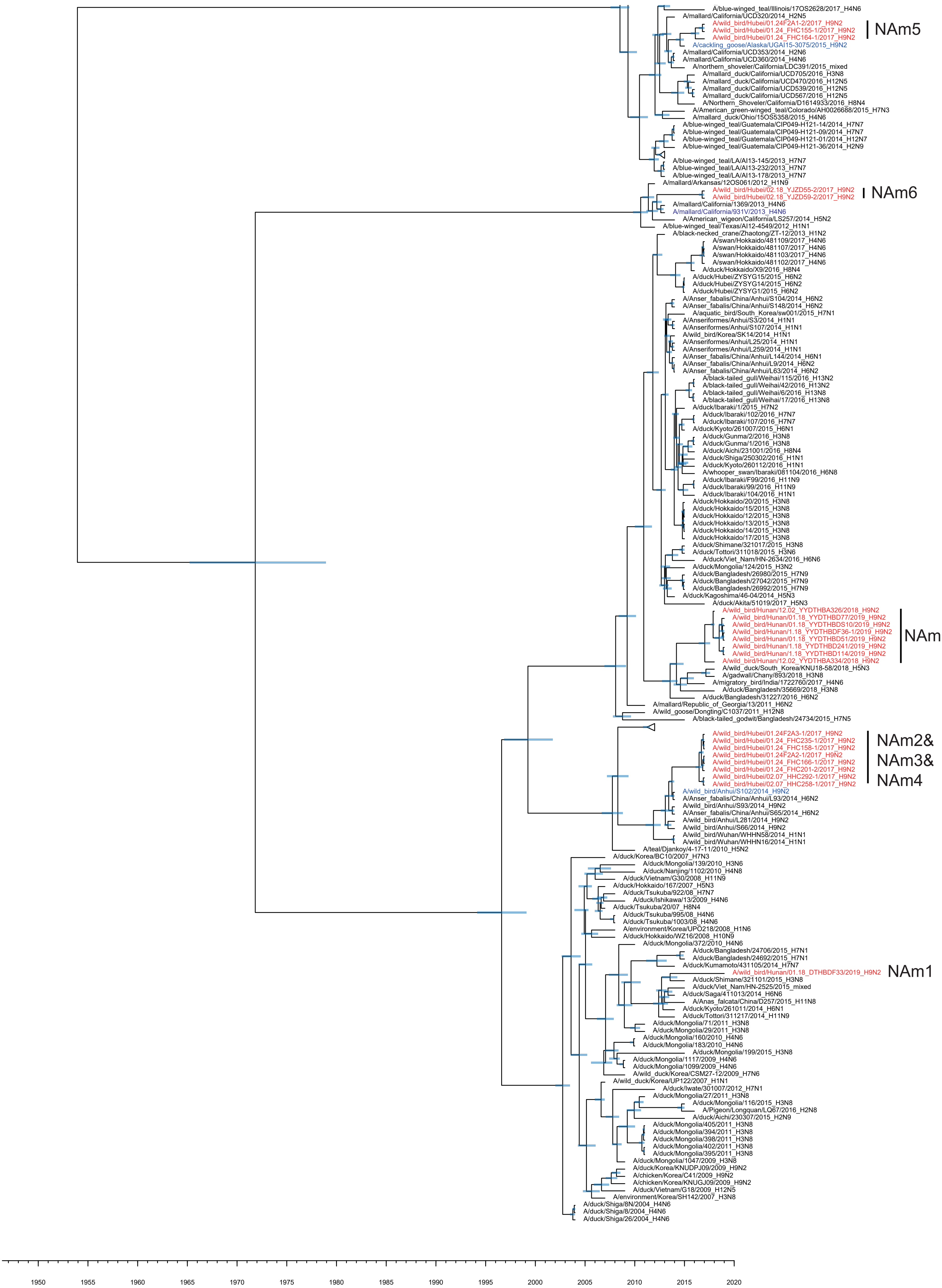

A/blue-winged_teal/Illinois/17OS2628/2017_H4N6
A/mallard/California/UCD320/2014_H2N5
A/wild_bird/Hubei/01.24F2A1-2/2017_H9N2
A/wild_bird/Hubei/01.24_FHC155-1/2017_H9N2
A/wild_bird/Hubei/01.24_FHC164-1/2017_H9N2
A/cackling_goose/Alaska/UGAI15-3075/2015_H9N2
A/mallard/California/UCD353/2014_H2N6
A/mallard/California/UCD360/2014_H4N6
A/northern_shoveler/California/LDC391/2015_mixed
A/mallard_duck/California/UCD705/2016_H3N8
A/mallard_duck/California/UCD470/2016_H12N5
A/mallard_duck/California/UCD539/2016_H12N5
A/mallard_duck/California/UCD567/2016_H12N5
A/Northern_Shoveler/California/D1614933/2016_H8N4
A/American_green-winged_teal/Colorado/AH0026688/2015_H7N3
A/mallard_duck/Ohio/15OS5358/2015_H4N6
A/blue-winged_teal/Guatemala/CIP049-H121-14/2014_H7N7
A/blue-winged_teal/Guatemala/CIP049-H121-09/2014_H7N7
A/blue-winged_teal/Guatemala/CIP049-H121-01/2014_H12N7
A/blue-winged_teal/Guatemala/CIP049-H121-36/2014_H2N9
A/blue-winged_teal/LA/AI13-145/2013_H7N7
A/blue-winged_teal/LA/AI13-232/2013_H7N7
A/blue-winged_teal/LA/AI13-178/2013_H7N7
A/mallard/Arkansas/12OS061/2012_H1N9
A/wild_bird/Hubei/02.18_YJZD55-2/2017_H9N2
A/wild_bird/Hubei/02.18_YJZD59-2/2017_H9N2
A/mallard/California/1369/2013_H4N6
A/mallard/California/931V/2013_H4N6
A/American_wigeon/California/LS257/2014_H5N2
A/blue-winged_teal/Texas/AI12-4549/2012_H1N1
A/black-necked_crane/Zhaotong/ZT-12/2013_H1N2
A/swan/Hokkaido/481109/2017_H4N6
A/swan/Hokkaido/481107/2017_H4N6
A/swan/Hokkaido/481103/2017_H4N6
A/swan/Hokkaido/481102/2017_H4N6
A/duck/Hokkaido/X9/2016_H8N4
A/duck/Hubei/ZYSYG15/2015_H6N2
A/duck/Hubei/ZYSYG14/2015_H6N2
A/duck/Hubei/ZYSYG1/2015_H6N2
A/Anser_fabalis/China/Anhui/S104/2014_H6N2
A/Anser_fabalis/China/Anhui/S148/2014_H6N2
A/aquatic_bird/South_Korea/sw001/2015_H7N1
A/Anseriformes/Anhui/S3/2014_H1N1
A/Anseriformes/Anhui/S107/2014_H1N1
A/wild_bird/Korea/SK14/2014_H1N1
A/Anseriformes/Anhui/L25/2014_H1N1
A/Anseriformes/Anhui/L259/2014_H1N1
A/Anser_fabalis/China/Anhui/L144/2014_H6N1
A/Anser_fabalis/China/Anhui/L9/2014_H6N2
A/Anser_fabalis/China/Anhui/L63/2014_H6N2
A/black-tailed_gull/Weihai/115/2016_H13N2
A/black-tailed_gull/Weihai/42/2016_H13N2
A/black-tailed_gull/Weihai/6/2016_H13N8
A/black-tailed_gull/Weihai/17/2016_H13N8
A/duck/Ibaraki/1/2015_H7N2
A/duck/Ibaraki/102/2016_H7N7
A/duck/Ibaraki/107/2016_H7N7
A/duck/Kyoto/261007/2015_H6N1
A/duck/Gunma/2/2016_H3N8
A/duck/Gunma/1/2016_H3N8
A/duck/Aichi/231001/2016_H8N4
A/duck/Shiga/250302/2016_H1N1
A/duck/Kyoto/260112/2016_H1N1
A/whooper_swan/Ibaraki/081104/2016_H6N8
A/duck/Ibaraki/F99/2016_H11N9
A/duck/Ibaraki/99/2016_H11N9
A/duck/Ibaraki/104/2016_H1N1
A/duck/Hokkaido/20/2015_H3N8
A/duck/Hokkaido/15/2015_H3N8
A/duck/Hokkaido/12/2015_H3N8
A/duck/Hokkaido/13/2015_H3N8
A/duck/Hokkaido/14/2015_H3N8
A/duck/Hokkaido/17/2015_H3N8
A/duck/Shimane/321017/2015_H3N8
A/duck/Tottori/311018/2015_H3N6
A/duck/Viet_Nam/HN-2634/2016_H6N6
A/duck/Mongolia/124/2015_H3N2
A/duck/Bangladesh/26980/2015_H7N9
A/duck/Bangladesh/27042/2015_H7N9
A/duck/Bangladesh/26992/2015_H7N9
A/duck/Kagoshima/46-04/2014_H5N3
A/duck/Akita/51019/2017_H5N3
A/wild_bird/Hunan/12.02_YYDTHBA326/2018_H9N2
A/wild_bird/Hunan/01.18_YYDTHBD77/2019_H9N2
A/wild_bird/Hunan/01.18_YYDTHBDS10/2019_H9N2
A/wild_bird/Hunan/1.18_YYDTHBDF36-1/2019_H9N2
A/wild_bird/Hunan/01.18_YYDTHBD51/2019_H9N2
A/wild_bird/Hunan/1.18_YYDTHBD241/2019_H9N2
A/wild_bird/Hunan/1.18_YYDTHBD114/2019_H9N2
A/wild_bird/Hunan/12.02_YYDTHBA334/2018_H9N2
A/wild_duck/South_Korea/KNU18-58/2018_H5N3
A/gadwall/Chany/893/2018_H3N8
A/migratory_bird/India/1722760/2017_H4N6
A/duck/Bangladesh/35669/2018_H3N8
A/duck/Bangladesh/31227/2016_H6N2
A/mallard/Republic_of_Georgia/13/2011_H6N2
A/wild_goose/Dongting/C1037/2011_H12N8
A/black-tailed_godwit/Bangladesh/24734/2015_H7N5
A/wild_bird/Hubei/01.24F2A3-1/2017_H9N2
A/wild_bird/Hubei/01.24_FHC235-1/2017_H9N2
A/wild_bird/Hubei/01.24_FHC158-1/2017_H9N2
A/wild_bird/Hubei/01.24F2A2-1/2017_H9N2
A/wild_bird/Hubei/01.24_FHC166-1/2017_H9N2
A/wild_bird/Hubei/01.24_FHC201-2/2017_H9N2
A/wild_bird/Hubei/02.07_HHC292-1/2017_H9N2
A/wild_bird/Hubei/02.07_HHC258-1/2017_H9N2
A/wild_bird/Anhui/S102/2014_H9N2
A/Anser_fabalis/China/Anhui/L93/2014_H6N2
A/wild_bird/Anhui/S93/2014_H9N2
A/Anser_fabalis/China/Anhui/S65/2014_H6N2
A/wild_bird/Anhui/L281/2014_H9N2
A/wild_bird/Anhui/S66/2014_H9N2
A/wild_bird/Wuhan/WHHN58/2014_H1N1
A/wild_bird/Wuhan/WHHN16/2014_H1N1
A/teal/Djankoy/4-17-11/2010_H5N2
A/duck/Korea/BC10/2007_H7N3
A/duck/Mongolia/139/2010_H3N6
A/duck/Nanjing/1102/2010_H4N8
A/duck/Vietnam/G30/2008_H11N9
A/duck/Hokkaido/167/2007_H5N3
A/duck/Tsukuba/922/08_H7N7
A/duck/Ishikawa/13/2009_H4N6
A/duck/Tsukuba/20/07_H8N4
A/duck/Tsukuba/995/08_H4N6
A/duck/Tsukuba/1003/08_H4N6
A/environment/Korea/UPO218/2008_H1N6
A/duck/Hokkaido/WZ16/2008_H10N9
A/duck/Mongolia/372/2010_H4N6
A/duck/Bangladesh/24706/2015_H7N1
A/duck/Bangladesh/24692/2015_H7N1
A/duck/Kumamoto/431105/2014_H7N7
A/wild_bird/Hunan/01.18_DTHBDF33/2019_H9N2
A/duck/Shimane/321101/2015_H3N8
A/duck/Viet_Nam/HN-2525/2015_mixed
A/duck/Saga/411013/2014_H6N6
A/Anas_falcata/China/D257/2015_H11N8
A/duck/Kyoto/261011/2014_H6N1
A/duck/Tottori/311217/2014_H11N9
A/duck/Mongolia/71/2011_H3N8
A/duck/Mongolia/29/2011_H3N8
A/duck/Mongolia/160/2010_H4N6
A/duck/Mongolia/183/2010_H4N6
A/duck/Mongolia/199/2015_H3N8
A/duck/Mongolia/1117/2009_H4N6
A/duck/Mongolia/1099/2009_H4N6
A/wild_duck/Korea/CSM27-12/2009_H7N6
A/wild_duck/Korea/UP122/2007_H1N1
A/duck/Iwate/301007/2012_H7N1
A/duck/Mongolia/27/2011_H3N8
A/duck/Mongolia/116/2015_H3N8
A/Pigeon/Longquan/LQ67/2016_H2N8
A/duck/Aichi/230307/2015_H2N9
A/duck/Mongolia/405/2011_H3N8
A/duck/Mongolia/394/2011_H3N8
A/duck/Mongolia/398/2011_H3N8
A/duck/Mongolia/402/2011_H3N8
A/duck/Mongolia/395/2011_H3N8
A/duck/Mongolia/1047/2009_H3N8
A/duck/Korea/KNUDPJ09/2009_H9N2
A/chicken/Korea/C41/2009_H9N2
A/chicken/Korea/KNUGJ09/2009_H9N2
A/duck/Vietnam/G18/2009_H12N5
A/environment/Korea/SH142/2007_H3N8
A/duck/Shiga/8N/2004_H4N6
A/duck/Shiga/8/2004_H4N6
A/duck/Shiga/26/2004_H4N6
NAm5
NAm6
NAm1
NAm2&
NAm3&
NAm4
NAm1
1950
1955
1960
1965
1970
1975
1980
1985
1990
1995
2000
2005
2010
2015
2020

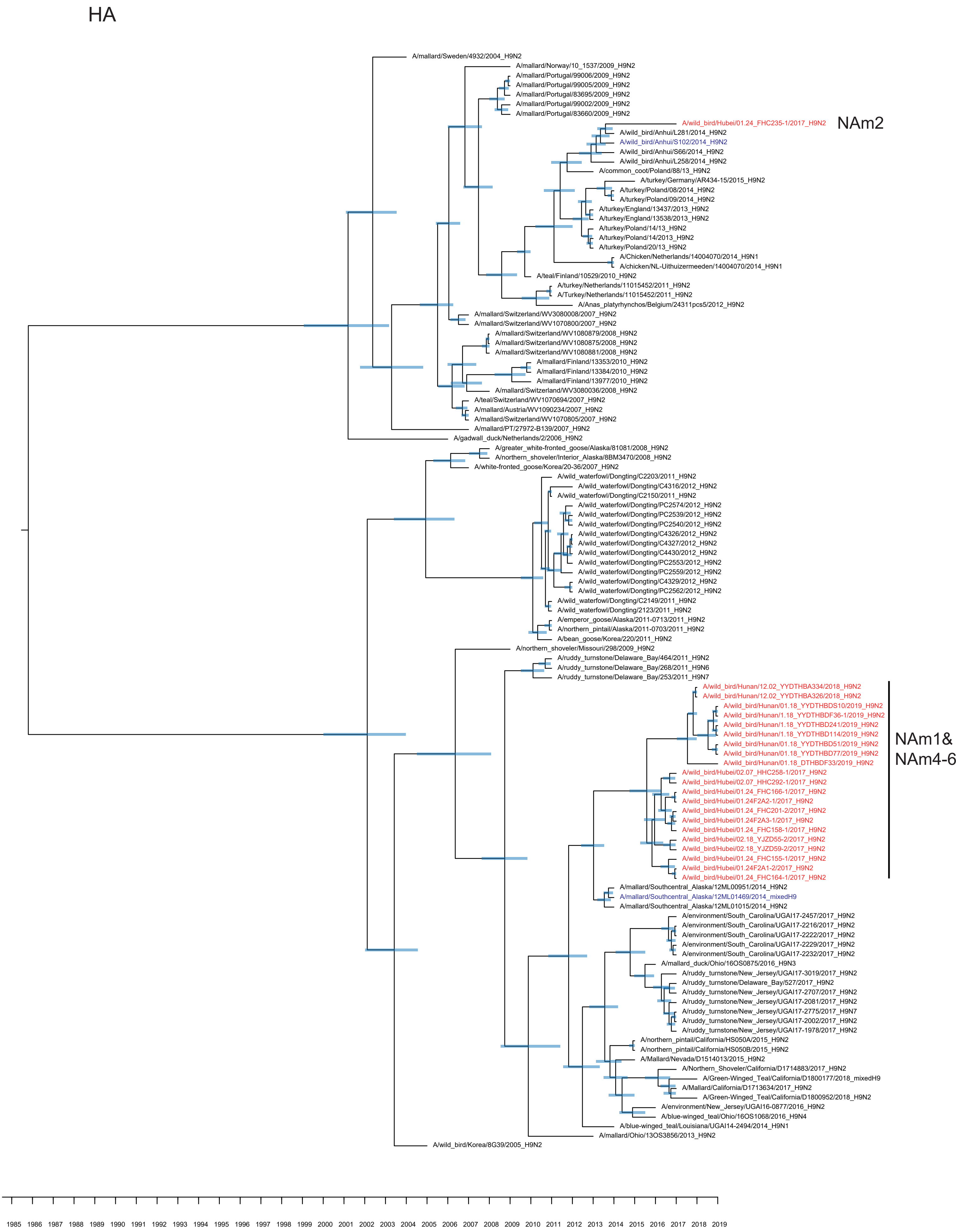
HA
A/mallard/Sweden/4932/2004_H9N2
A/mallard/Norway/10_1537/2009_H9N2
A/mallard/Portugal/99006/2009_H9N2
A/mallard/Portugal/99005/2009_H9N2
A/mallard/Portugal/83695/2009_H9N2
A/mallard/Portugal/99002/2009_H9N2
A/mallard/Portugal/83660/2009_H9N2
A/wild_bird/Hubei/01.24_FHC235-1/2017_H9N2
NAm2
A/wild_bird/Anhui/L281/2014_H9N2
A/wild_bird/Anhui/S102/2014_H9N2
A/wild_bird/Anhui/S66/2014_H9N2
A/wild_bird/Anhui/L258/2014_H9N2
A/common_coot/Poland/88/13_H9N2
A/turkey/Germany/AR434-15/2015_H9N2
A/turkey/Poland/08/2014_H9N2
A/turkey/Poland/09/2014_H9N2
A/turkey/England/13437/2013_H9N2
A/turkey/England/13538/2013_H9N2
A/turkey/Poland/14/13_H9N2
A/turkey/Poland/14/2013_H9N2
A/turkey/Poland/20/13_H9N2
A/Chicken/Netherlands/14004070/2014_H9N1
A/chicken/NL-Uithuizermeeden/14004070/2014_H9N1
A/teal/Finland/10529/2010_H9N2
A/turkey/Netherlands/11015452/2011_H9N2
A/Turkey/Netherlands/11015452/2011_H9N2
A/Anas_platyrhynchos/Belgium/24311pcs5/2012_H9N2
A/mallard/Switzerland/WV3080008/2007_H9N2
A/mallard/Switzerland/WV1070800/2007_H9N2
A/mallard/Switzerland/WV1080879/2008_H9N2
A/mallard/Switzerland/WV1080875/2008_H9N2
A/mallard/Switzerland/WV1080881/2008_H9N2
A/mallard/Finland/13353/2010_H9N2
A/mallard/Finland/13384/2010_H9N2
A/mallard/Finland/13977/2010_H9N2
A/mallard/Switzerland/WV3080036/2008_H9N2
A/teal/Switzerland/WV1070694/2007_H9N2
A/mallard/Austria/WV1090234/2007_H9N2
A/mallard/Switzerland/WV1070805/2007_H9N2
A/mallard/PT/27972-B139/2007_H9N2
A/gadwall_duck/Netherlands/2/2006_H9N2
A/greater_white-fronted_goose/Alaska/81081/2008_H9N2
A/northern_shoveler/Interior_Alaska/8BM3470/2008_H9N2
A/white-fronted_goose/Korea/20-36/2007_H9N2
A/wild_waterfowl/Dongting/C2203/2011_H9N2
A/wild_waterfowl/Dongting/C4316/2012_H9N2
A/wild_waterfowl/Dongting/C2150/2011_H9N2
A/wild_waterfowl/Dongting/PC2574/2012_H9N2
A/wild_waterfowl/Dongting/PC2539/2012_H9N2
A/wild_waterfowl/Dongting/PC2540/2012_H9N2
A/wild_waterfowl/Dongting/C4326/2012_H9N2
A/wild_waterfowl/Dongting/C4327/2012_H9N2
A/wild_waterfowl/Dongting/C4430/2012_H9N2
A/wild_waterfowl/Dongting/PC2553/2012_H9N2
A/wild_waterfowl/Dongting/PC2559/2012_H9N2
A/wild_waterfowl/Dongting/C4329/2012_H9N2
A/wild_waterfowl/Dongting/PC2562/2012_H9N2
A/wild_waterfowl/Dongting/C2149/2011_H9N2
A/wild_waterfowl/Dongting/2123/2011_H9N2
A/emperor_goose/Alaska/2011-0713/2011_H9N2
A/northern_pintail/Alaska/2011-0703/2011_H9N2
A/bean_goose/Korea/220/2011_H9N2
A/northern_shoveler/Missouri/298/2009_H9N2
A/ruddy_turnstone/Delaware_Bay/464/2011_H9N2
A/ruddy_turnstone/Delaware_Bay/268/2011_H9N6
A/ruddy_turnstone/Delaware_Bay/253/2011_H9N7
A/wild_bird/Hunan/12.02_YYDTHBA334/2018_H9N2
A/wild_bird/Hunan/12.02_YYDTHBA326/2018_H9N2
A/wild_bird/Hunan/01.18_YYDTHBDS10/2019_H9N2
A/wild_bird/Hunan/1.18_YYDTHBDF36-1/2019_H9N2
A/wild_bird/Hunan/1.18_YYDTHBD241/2019_H9N2
A/wild_bird/Hunan/1.18_YYDTHBD114/2019_H9N2
A/wild_bird/Hunan/01.18_YYDTHBD51/2019_H9N2
A/wild_bird/Hunan/01.18_YYDTHBD77/2019_H9N2
A/wild_bird/Hunan/01.18_DTHBDF33/2019_H9N2
NAm1&
NAm4-6
A/wild_bird/Hubei/02.07_HHC258-1/2017_H9N2
A/wild_bird/Hubei/02.07_HHC292-1/2017_H9N2
A/wild_bird/Hubei/01.24_FHC166-1/2017_H9N2
A/wild_bird/Hubei/01.24F2A2-1/2017_H9N2
A/wild_bird/Hubei/01.24_FHC201-2/2017_H9N2
A/wild_bird/Hubei/01.24F2A3-1/2017_H9N2
A/wild_bird/Hubei/01.24_FHC158-1/2017_H9N2
A/wild_bird/Hubei/02.18_YJZD55-2/2017_H9N2
A/wild_bird/Hubei/02.18_YJZD59-2/2017_H9N2
A/wild_bird/Hubei/01.24_FHC155-1/2017_H9N2
A/wild_bird/Hubei/01.24F2A1-2/2017_H9N2
A/wild_bird/Hubei/01.24_FHC164-1/2017_H9N2
A/mallard/Southcentral_Alaska/12ML00951/2014_H9N2
A/mallard/Southcentral_Alaska/12ML01469/2014_mixedH9
A/mallard/Southcentral_Alaska/12ML01015/2014_H9N2
A/environment/South_Carolina/UGAI17-2457/2017_H9N2
A/environment/South_Carolina/UGAI17-2216/2017_H9N2
A/environment/South_Carolina/UGAI17-2222/2017_H9N2
A/environment/South_Carolina/UGAI17-2229/2017_H9N2
A/environment/South_Carolina/UGAI17-2232/2017_H9N2
A/mallard_duck/Ohio/16OS0875/2016_H9N3
A/ruddy_turnstone/New_Jersey/UGAI17-3019/2017_H9N2
A/ruddy_turnstone/Delaware_Bay/527/2017_H9N2
A/ruddy_turnstone/New_Jersey/UGAI17-2707/2017_H9N2
A/ruddy_turnstone/New_Jersey/UGAI17-2081/2017_H9N2
A/ruddy_turnstone/New_Jersey/UGAI17-2775/2017_H9N7
A/ruddy_turnstone/New_Jersey/UGAI17-2002/2017_H9N2
A/ruddy_turnstone/New_Jersey/UGAI17-1978/2017_H9N2
A/northern_pintail/California/HS050A/2015_H9N2
A/northern_pintail/California/HS050B/2015_H9N2
A/Mallard/Nevada/D1514013/2015_H9N2
A/Northern_Shoveler/California/D1714883/2017_H9N2
A/Green-Winged_Teal/California/D1800177/2018_mixedH9
A/Mallard/California/D1713634/2017_H9N2
A/Green-Winged_Teal/California/D1800952/2018_H9N2
A/environment/New_Jersey/UGAI16-0877/2016_H9N2
A/blue-winged_teal/Ohio/16OS1068/2016_H9N4
A/blue-winged_teal/Louisiana/UGAI14-2494/2014_H9N1
A/mallard/Ohio/13OS3856/2013_H9N2
A/wild_bird/Korea/8G39/2005_H9N2
1985 1986 1987 1988 1989 1990 1991 1992 1993 1994 1995 1996 1997 1998 1999 2000 2001 2002 2003 2004 2005 2006 2007 2008 2009 2010 2011 2012 2013 2014 2015 2016 2017 2018 2019

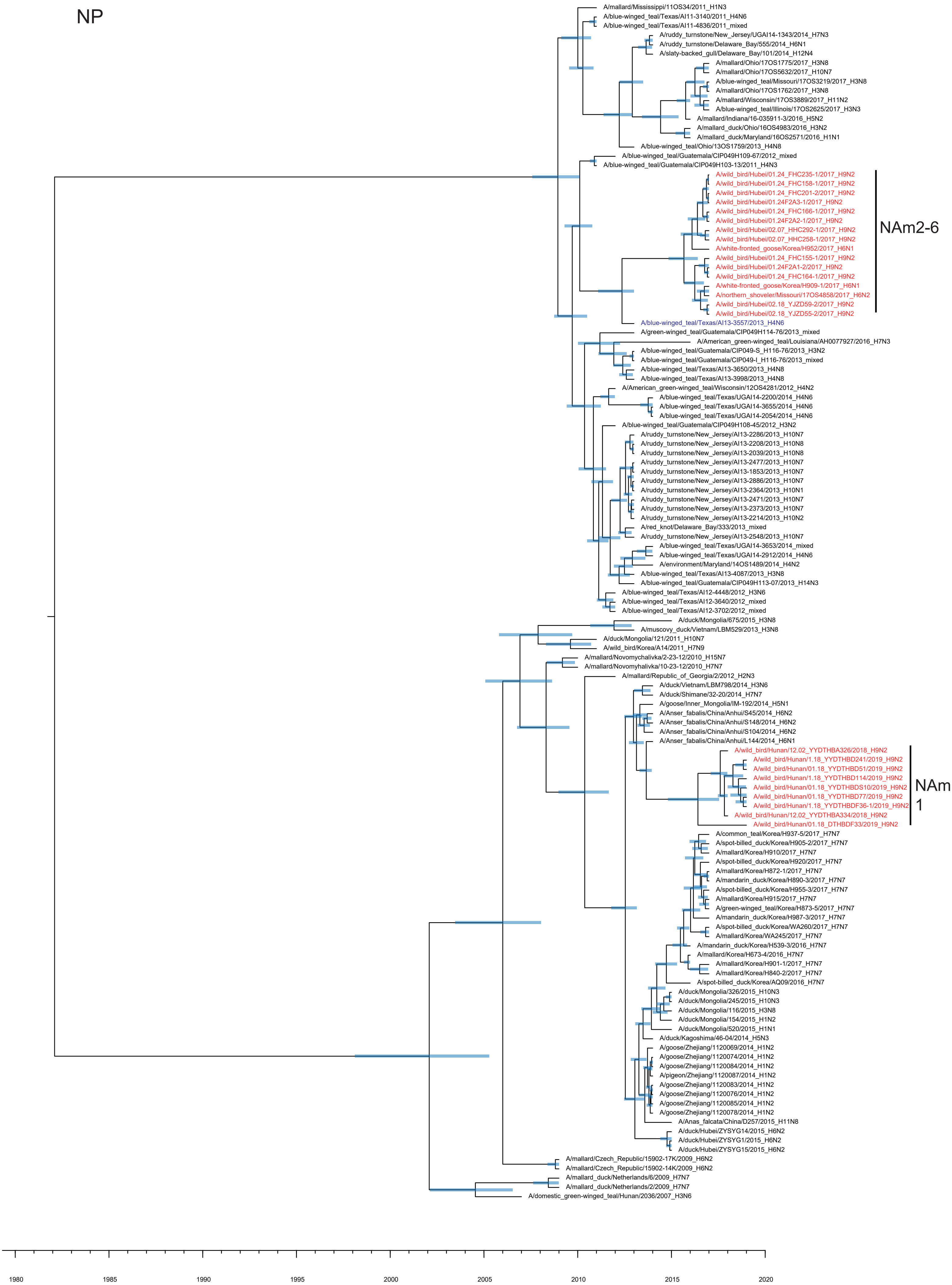

NP
A/mallard/Mississippi/11OS34/2011_H1N3
A/blue-winged_teal/Texas/AI11-3140/2011_H4N6
A/blue-winged_teal/Texas/AI11-4836/2011_mixed
A/ruddy_turnstone/New_Jersey/UGAI14-1343/2014_H7N3
A/ruddy_turnstone/Delaware_Bay/555/2014_H6N1
A/slaty-backed_gull/Delaware_Bay/101/2014_H12N4
A/mallard/Ohio/17OS1775/2017_H3N8
A/mallard/Ohio/17OS5632/2017_H10N7
A/blue-winged_teal/Missouri/17OS3219/2017_H3N8
A/mallard/Ohio/17OS1762/2017_H3N8
A/mallard/Wisconsin/17OS3889/2017_H11N2
A/blue-winged_teal/Illinois/17OS2625/2017_H3N3
A/mallard/Indiana/16-035911-3/2016_H5N2
A/mallard_duck/Ohio/16OS4983/2016_H3N2
A/mallard_duck/Maryland/16OS2571/2016_H1N1
A/blue-winged_teal/Ohio/13OS1759/2013_H4N8
A/blue-winged_teal/Guatemala/CIP049H109-67/2012_mixed
A/blue-winged_teal/Guatemala/CIP049H103-13/2011_H4N3
A/wild_bird/Hubei/01.24_FHC235-1/2017_H9N2
A/wild_bird/Hubei/01.24_FHC158-1/2017_H9N2
A/wild_bird/Hubei/01.24_FHC201-2/2017_H9N2
A/wild_bird/Hubei/01.24F2A3-1/2017_H9N2
A/wild_bird/Hubei/01.24_FHC166-1/2017_H9N2
A/wild_bird/Hubei/01.24F2A2-1/2017_H9N2
A/wild_bird/Hubei/02.07_HHC292-1/2017_H9N2
A/wild_bird/Hubei/02.07_HHC258-1/2017_H9N2
A/white-fronted_goose/Korea/H952/2017_H6N1
A/wild_bird/Hubei/01.24_FHC155-1/2017_H9N2
A/wild_bird/Hubei/01.24F2A1-2/2017_H9N2
A/wild_bird/Hubei/01.24_FHC164-1/2017_H9N2
A/white-fronted_goose/Korea/H909-1/2017_H6N1
A/northern_shoveler/Missouri/17OS4858/2017_H6N2
A/wild_bird/Hubei/02.18_YJZD59-2/2017_H9N2
A/wild_bird/Hubei/02.18_YJZD55-2/2017_H9N2
NAm2-6
A/blue-winged_teal/Texas/AI13-3557/2013_H4N6
A/green-winged_teal/Guatemala/CIP049H114-76/2013_mixed
A/American_green-winged_teal/Louisiana/AH0077927/2016_H7N3
A/blue-winged_teal/Guatemala/CIP049-S_H116-76/2013_H3N2
A/blue-winged_teal/Guatemala/CIP049-I_H116-76/2013_mixed
A/blue-winged_teal/Texas/AI13-3650/2013_H4N8
A/blue-winged_teal/Texas/AI13-3998/2013_H4N8
A/American_green-winged_teal/Wisconsin/12OS4281/2012_H4N2
A/blue-winged_teal/Texas/UGAI14-2200/2014_H4N6
A/blue-winged_teal/Texas/UGAI14-3655/2014_H4N6
A/blue-winged_teal/Texas/UGAI14-2054/2014_H4N6
A/blue-winged_teal/Guatemala/CIP049H108-45/2012_H3N2
A/ruddy_turnstone/New_Jersey/AI13-2286/2013_H10N7
A/ruddy_turnstone/New_Jersey/AI13-2208/2013_H10N8
A/ruddy_turnstone/New_Jersey/AI13-2039/2013_H10N8
A/ruddy_turnstone/New_Jersey/AI13-2477/2013_H10N7
A/ruddy_turnstone/New_Jersey/AI13-1853/2013_H10N7
A/ruddy_turnstone/New_Jersey/AI13-2886/2013_H10N7
A/ruddy_turnstone/New_Jersey/AI13-2364/2013_H10N1
A/ruddy_turnstone/New_Jersey/AI13-2471/2013_H10N7
A/ruddy_turnstone/New_Jersey/AI13-2373/2013_H10N7
A/ruddy_turnstone/New_Jersey/AI13-2214/2013_H10N2
A/red_knot/Delaware_Bay/333/2013_mixed
A/ruddy_turnstone/New_Jersey/AI13-2548/2013_H10N7
A/blue-winged_teal/Texas/UGAI14-3653/2014_mixed
A/blue-winged_teal/Texas/UGAI14-2912/2014_H4N6
A/environment/Maryland/14OS1489/2014_H4N2
A/blue-winged_teal/Texas/AI13-4087/2013_H3N8
A/blue-winged_teal/Guatemala/CIP049H113-07/2013_H14N3
A/blue-winged_teal/Texas/AI12-4448/2012_H3N6
A/blue-winged_teal/Texas/AI12-3640/2012_mixed
A/blue-winged_teal/Texas/AI12-3702/2012_mixed
A/duck/Mongolia/675/2015_H3N8
A/muscovy_duck/Vietnam/LBM529/2013_H3N8
A/duck/Mongolia/121/2011_H10N7
A/wild_bird/Korea/A14/2011_H7N9
A/mallard/Novomychalivka/2-23-12/2010_H15N7
A/mallard/Novomyhalivka/10-23-12/2010_H7N7
A/mallard/Republic_of_Georgia/2/2012_H2N3
A/duck/Vietnam/LBM798/2014_H3N6
A/duck/Shimane/32-20/2014_H7N7
A/goose/Inner_Mongolia/IM-192/2014_H5N1
A/Anser_fabalis/China/Anhui/S45/2014_H6N2
A/Anser_fabalis/China/Anhui/S148/2014_H6N2
A/Anser_fabalis/China/Anhui/S104/2014_H6N2
A/Anser_fabalis/China/Anhui/L144/2014_H6N1
A/wild_bird/Hunan/12.02_YYDTHBA326/2018_H9N2
A/wild_bird/Hunan/1.18_YYDTHBD241/2019_H9N2
A/wild_bird/Hunan/01.18_YYDTHBD51/2019_H9N2
A/wild_bird/Hunan/1.18_YYDTHBD114/2019_H9N2
A/wild_bird/Hunan/01.18_YYDTHBDS10/2019_H9N2
A/wild_bird/Hunan/01.18_YYDTHBD77/2019_H9N2
A/wild_bird/Hunan/1.18_YYDTHBDF36-1/2019_H9N2
A/wild_bird/Hunan/12.02_YYDTHBA334/2018_H9N2
A/wild_bird/Hunan/01.18_DTHBDF33/2019_H9N2
NAm
1
A/common_teal/Korea/H937-5/2017_H7N7
A/spot-billed_duck/Korea/H905-2/2017_H7N7
A/mallard/Korea/H910/2017_H7N7
A/spot-billed_duck/Korea/H920/2017_H7N7
A/mallard/Korea/H872-1/2017_H7N7
A/mandarin_duck/Korea/H890-3/2017_H7N7
A/spot-billed_duck/Korea/H955-3/2017_H7N7
A/mallard/Korea/H915/2017_H7N7
A/green-winged_teal/Korea/H873-5/2017_H7N7
A/mandarin_duck/Korea/H987-3/2017_H7N7
A/spot-billed_duck/Korea/WA260/2017_H7N7
A/mallard/Korea/WA245/2017_H7N7
A/mandarin_duck/Korea/H539-3/2016_H7N7
A/mallard/Korea/H673-4/2016_H7N7
A/mallard/Korea/H901-1/2017_H7N7
A/mallard/Korea/H840-2/2017_H7N7
A/spot-billed_duck/Korea/AQ09/2016_H7N7
A/duck/Mongolia/326/2015_H10N3
A/duck/Mongolia/245/2015_H10N3
A/duck/Mongolia/116/2015_H3N8
A/duck/Mongolia/154/2015_H1N2
A/duck/Mongolia/520/2015_H1N1
A/duck/Kagoshima/46-04/2014_H5N3
A/goose/Zhejiang/1120069/2014_H1N2
A/goose/Zhejiang/1120074/2014_H1N2
A/goose/Zhejiang/1120084/2014_H1N2
A/pigeon/Zhejiang/1120087/2014_H1N2
A/goose/Zhejiang/1120083/2014_H1N2
A/goose/Zhejiang/1120076/2014_H1N2
A/goose/Zhejiang/1120085/2014_H1N2
A/goose/Zhejiang/1120078/2014_H1N2
A/Anas_falcata/China/D257/2015_H11N8
A/duck/Hubei/ZYSYG14/2015_H6N2
A/duck/Hubei/ZYSYG1/2015_H6N2
A/duck/Hubei/ZYSYG15/2015_H6N2
A/mallard/Czech_Republic/15902-17K/2009_H6N2
A/mallard/Czech_Republic/15902-14K/2009_H6N2
A/mallard_duck/Netherlands/6/2009_H7N7
A/mallard_duck/Netherlands/2/2009_H7N7
A/domestic_green-winged_teal/Hunan/2036/2007_H3N6
1980
1985
1990
1995
2000
2005
2010
2015
2020

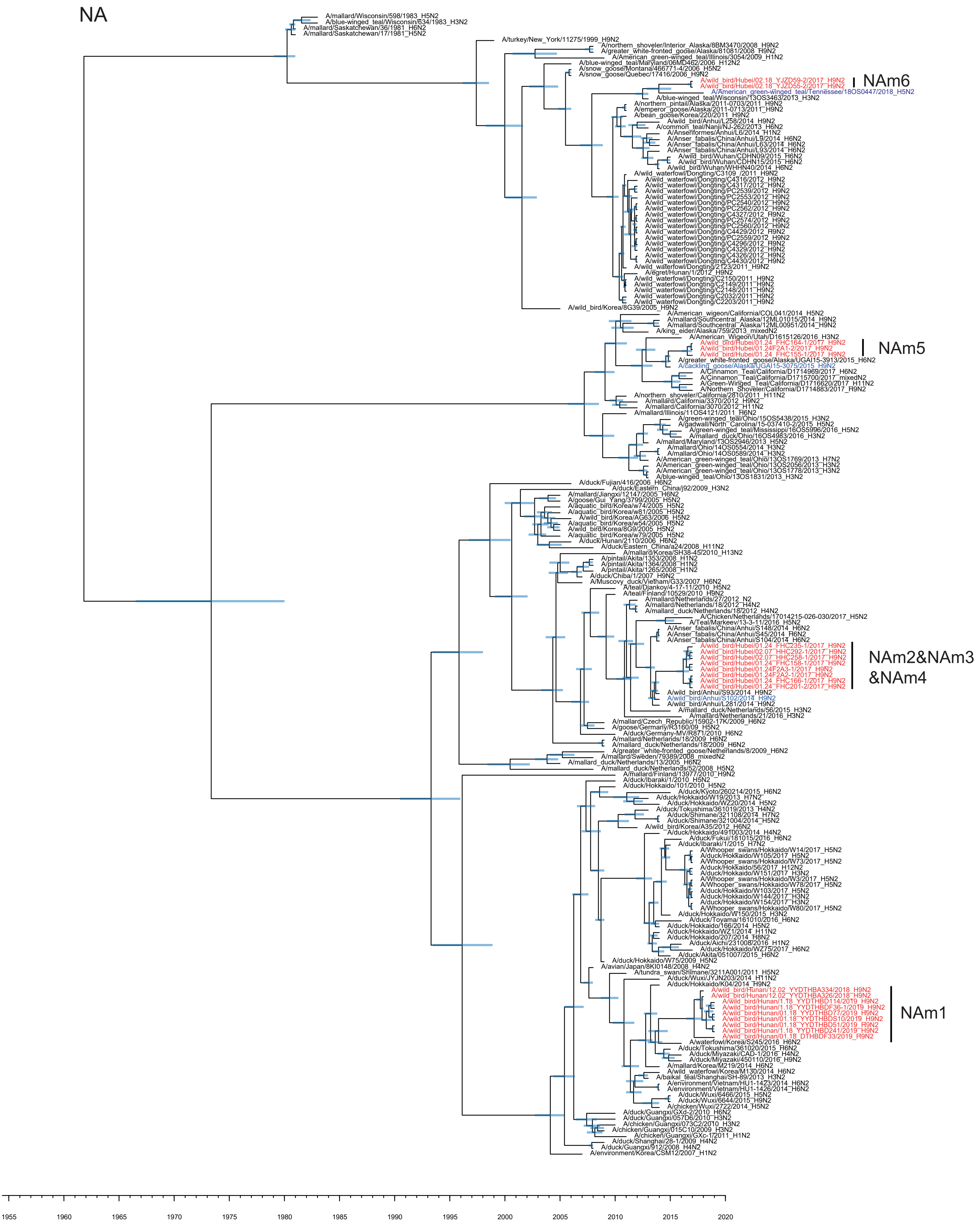
NA
A/mallard/Wisconsin/598/1983_H5N2
A/blue-winged_teal/Wisconsin/634/1983_H3N2
A/mallard/Saskatchewan/36/1981_H6N2
A/mallard/Saskatchewan/17/1981_H5N2
A/turkey/New_York/11275/1999_H9N2
A/northern_shoveler/Interior_Alaska/8BM3470/2008_H9N2
A/greater_white-fronted_goose/Alaska/81081/2008_H9N2
A/American_green-winged_teal/Illinois/3054/2009_H1N2
A/blue-winged_teal/Maryland/06MD462/2006_H12N2
A/snow_goose/Montana/466771-4/2006_H5N2
A/snow_goose/Quebec/17416/2006_H9N2
A/wild_bird/Hubei/02.18_YJZD59-2/2017_H9N2
A/wild_bird/Hubei/02.18_YJZD55-2/2017_H9N2
NAm6
A/American_green-winged_teal/Tennessee/18OS0447/2018_H5N2
A/blue-winged_teal/Wisconsin/13OS3463/2013_H3N2
A/northern_pintail/Alaska/2011-0703/2011_H9N2
A/emperor_goose/Alaska/2011-0713/2011_H9N2
A/bean_goose/Korea/220/2011_H9N2
A/wild_bird/Anhui/L258/2014_H9N2
A/common_teal/Nanji/NJ-262/2013_H6N2
A/Anseriformes/Anhui/L6/2014_H1N2
A/Anser_fabalis/China/Anhui/L9/2014_H6N2
A/Anser_fabalis/China/Anhui/L63/2014_H6N2
A/Anser_fabalis/China/Anhui/L93/2014_H6N2
A/wild_bird/Wuhan/CDHN09/2015_H6N2
A/wild_bird/Wuhan/CDHN15/2015_H6N2
A/wild_bird/Wuhan/WHHN40/2014_H6N2
A/wild_waterfowl/Dongting/C3109_/2011_H9N2
A/wild_waterfowl/Dongting/C4316/2012_H9N2
A/wild_waterfowl/Dongting/C4317/2012_H9N2
A/wild_waterfowl/Dongting/PC2539/2012_H9N2
A/wild_waterfowl/Dongting/PC2553/2012_H9N2
A/wild_waterfowl/Dongting/PC2540/2012_H9N2
A/wild_waterfowl/Dongting/PC2562/2012_H9N2
A/wild_waterfowl/Dongting/C4327/2012_H9N2
A/wild_waterfowl/Dongting/PC2574/2012_H9N2
A/wild_waterfowl/Dongting/PC2560/2012_H9N2
A/wild_waterfowl/Dongting/C4429/2012_H9N2
A/wild_waterfowl/Dongting/PC2559/2012_H9N2
A/wild_waterfowl/Dongting/C4296/2012_H9N2
A/wild_waterfowl/Dongting/C4329/2012_H9N2
A/wild_waterfowl/Dongting/C4326/2012_H9N2
A/wild_waterfowl/Dongting/C4430/2012_H9N2
A/wild_waterfowl/Dongting/2123/2011_H9N2
A/egret/Hunan/1/2012_H9N2
A/wild_waterfowl/Dongting/C2150/2011_H9N2
A/wild_waterfowl/Dongting/C2149/2011_H9N2
A/wild_waterfowl/Dongting/C2148/2011_H9N2
A/wild_waterfowl/Dongting/C2032/2011_H9N2
A/wild_waterfowl/Dongting/C2203/2011_H9N2
A/wild_bird/Korea/8G39/2005_H9N2
A/American_wigeon/California/COL041/2014_H5N2
A/mallard/Southcentral_Alaska/12ML01015/2014_H9N2
A/mallard/Southcentral_Alaska/12ML00951/2014_H9N2
A/king_eider/Alaska/759/2013_mixedN2
A/American_Wigeon/Utah/D1615126/2016_H3N2
A/wild_bird/Hubei/01.24_FHC164-1/2017_H9N2
A/wild_bird/Hubei/01.24F2A1-2/2017_H9N2
A/wild_bird/Hubei/01.24_FHC155-1/2017_H9N2
NAm5
A/greater_white-fronted_goose/Alaska/UGAI15-3913/2015_H6N2
A/cackling_goose/Alaska/UGAI15-3075/2015_H9N2
A/Cinnamon_Teal/California/D1714969/2017_H6N2
A/Cinnamon_Teal/California/D1715700/2017_mixedN2
A/Green-Winged_Teal/California/D1716620/2017_H11N2
A/Northern_Shoveler/California/D1714883/2017_H9N2
A/northern_shoveler/California/2810/2011_H11N2
A/mallard/California/3370/2012_H9N2
A/mallard/California/3070/2012_H11N2
A/mallard/Illinois/11OS4121/2011_H6N2
A/green-winged_teal/Ohio/15OS5438/2015_H3N2
A/gadwall/North_Carolina/15-037410-2/2015_H5N2
A/green-winged_teal/Mississippi/16OS5996/2016_H5N2
A/mallard_duck/Ohio/16OS4983/2016_H3N2
A/mallard/Maryland/13OS2946/2013_H5N2
A/mallard/Ohio/14OS0554/2014_H3N2
A/mallard/Ohio/14OS0589/2014_H3N2
A/American_green-winged_teal/Ohio/13OS1769/2013_H7N2
A/American_green-winged_teal/Ohio/13OS2056/2013_H3N2
A/American_green-winged_teal/Ohio/13OS1778/2013_H3N2
A/blue-winged_teal/Ohio/13OS1831/2013_H3N2
A/duck/Fujian/416/2006_H6N2
A/duck/Eastern_China/j92/2009_H3N2
A/mallard/Jiangxi/12147/2005_H6N2
A/goose/Gui_Yang/3799/2005_H5N2
A/aquatic_bird/Korea/w74/2005_H5N2
A/aquatic_bird/Korea/w81/2005_H5N2
A/wild_bird/Korea/AG63/2006_H5N2
A/aquatic_bird/Korea/w54/2005_H5N2
A/wild_bird/Korea/8G9/2005_H5N2
A/aquatic_bird/Korea/w79/2005_H5N2
A/duck/Hunan/2110/2006_H6N2
A/duck/Eastern_China/a24/2008_H11N2
A/mallard/Korea/SH38-45/2010_H13N2
A/pintail/Akita/1353/2008_H1N2
A/pintail/Akita/1364/2008_H1N2
A/pintail/Akita/1265/2008_H1N2
A/duck/Chiba/1/2007_H9N2
A/Muscovy_duck/Vietnam/G33/2007_H6N2
A/teal/Djankoy/4-17-11/2010_H5N2
A/teal/Finland/10529/2010_H9N2
A/mallard/Netherlands/27/2012_N2
A/mallard/Netherlands/18/2012_H4N2
A/mallard_duck/Netherlands/18/2012_H4N2
A/Chicken/Netherlands/17014215-026-030/2017_H5N2
A/Teal/Markeev/13-3-11/2016_H5N2
A/Anser_fabalis/China/Anhui/S148/2014_H6N2
A/Anser_fabalis/China/Anhui/S45/2014_H6N2
A/Anser_fabalis/China/Anhui/S104/2014_H6N2
A/wild_bird/Hubei/01.24_FHC235-1/2017_H9N2
A/wild_bird/Hubei/02.07_HHC292-1/2017_H9N2
A/wild_bird/Hubei/02.07_HHC258-1/2017_H9N2
A/wild_bird/Hubei/01.24_FHC158-1/2017_H9N2
A/wild_bird/Hubei/01.24F2A3-1/2017_H9N2
A/wild_bird/Hubei/01.24F2A2-1/2017_H9N2
A/wild_bird/Hubei/01.24_FHC166-1/2017_H9N2
A/wild_bird/Hubei/01.24_FHC201-2/2017_H9N2
NAm2&NAm3
&NAm4
A/wild_bird/Anhui/S93/2014_H9N2
A/wild_bird/Anhui/S102/2014_H9N2
A/wild_bird/Anhui/L281/2014_H9N2
A/mallard_duck/Netherlands/56/2015_H3N2
A/mallard/Netherlands/21/2016_H3N2
A/mallard/Czech_Republic/15902-17K/2009_H6N2
A/goose/Germany/R3160/09_H5N2
A/duck/Germany-MV/R871/2010_H6N2
A/mallard/Netherlands/18/2009_H6N2
A/mallard_duck/Netherlands/18/2009_H6N2
A/greater_white-fronted_goose/Netherlands/8/2009_H6N2
A/mallard/Sweden/79389/2008_mixedN2
A/mallard_duck/Netherlands/13/2005_H6N2
A/mallard_duck/Netherlands/52/2008_H5N2
A/mallard/Finland/13977/2010_H9N2
A/duck/Ibaraki/1/2010_H5N2
A/duck/Hokkaido/101/2010_H5N2
A/duck/Kyoto/260214/2015_H6N2
A/duck/Hokkaido/W19/2013_H7N2
A/duck/Hokkaido/WZ20/2014_H5N2
A/duck/Tokushima/361019/2013_H4N2
A/duck/Shimane/321108/2014_H7N2
A/duck/Shimane/321004/2014_H5N2
A/wild_bird/Korea/A35/2012_H6N2
A/duck/Hokkaido/491003/2014_H4N2
A/duck/Fukui/181015/2016_H6N2
A/duck/Ibaraki/1/2015_H7N2
A/Whooper_swans/Hokkaido/W14/2017_H5N2
A/duck/Hokkaido/W105/2017_H5N2
A/Whooper_swans/Hokkaido/W73/2017_H5N2
A/duck/Hokkaido/56/2017_H12N2
A/duck/Hokkaido/W151/2017_H3N2
A/Whooper_swans/Hokkaido/W3/2017_H5N2
A/Whooper_swans/Hokkaido/W78/2017_H5N2
A/duck/Hokkaido/W103/2017_H5N2
A/duck/Hokkaido/W144/2017_H3N2
A/duck/Hokkaido/W154/2017_H3N2
A/Whooper_swans/Hokkaido/W80/2017_H5N2
A/duck/Hokkaido/W150/2015_H3N2
A/duck/Toyama/161010/2016_H6N2
A/duck/Hokkaido/166/2014_H5N2
A/duck/Hokkaido/WZ1/2014_H11N2
A/duck/Hokkaido/207/2014_H8N2
A/duck/Aichi/231008/2016_H1N2
A/duck/Hokkaido/WZ75/2017_H6N2
A/duck/Akita/051007/2015_H6N2
A/duck/Hokkaido/W75/2009_H5N2
A/avian/Japan/8KI0148/2008_H4N2
A/tundra_swan/Shimane/3211A001/2011_H5N2
A/duck/Wuxi/JYJN203/2014_H11N2
A/duck/Hokkaido/K04/2014_H9N2
A/wild_bird/Hunan/12.02_YYDTHBA334/2018_H9N2
A/wild_bird/Hunan/12.02_YYDTHBA326/2018_H9N2
A/wild_bird/Hunan/1.18_YYDTHBD114/2019_H9N2
A/wild_bird/Hunan/1.18_YYDTHBDF36-1/2019_H9N2
A/wild_bird/Hunan/01.18_YYDTHBD77/2019_H9N2
A/wild_bird/Hunan/01.18_YYDTHBDS10/2019_H9N2
A/wild_bird/Hunan/01.18_YYDTHBD51/2019_H9N2
A/wild_bird/Hunan/1.18_YYDTHBD241/2019_H9N2
A/wild_bird/Hunan/01.18_DTHBDF33/2019_H9N2
NAm1
A/waterfowl/Korea/S245/2016_H6N2
A/duck/Tokushima/361020/2015_H6N2
A/duck/Miyazaki/CAD-1/2016_H4N2
A/duck/Miyazaki/450110/2016_H9N2
A/mallard/Korea/M219/2014_H6N2
A/wild_waterfowl/Korea/M130/2014_H6N2
A/baikal_teal/Shanghai/SH-89/2013_H3N2
A/environment/Vietnam/HU1-1423/2014_H6N2
A/environment/Vietnam/HU1-1426/2014_H6N2
A/duck/Wuxi/6466/2015_H5N2
A/duck/Wuxi/6644/2015_H9N2
A/chicken/Wuxi/2722/2014_H5N2
A/duck/Guangxi/GXd-2/2010_H6N2
A/duck/Guangxi/057D6/2010_H3N2
A/chicken/Guangxi/073C2/2010_H3N2
A/chicken/Guangxi/015C10/2009_H3N2
A/chicken/Guangxi/GXc-1/2011_H1N2
A/duck/Shanghai/28-1/2009_H4N2
A/duck/Guangxi/912/2008_H4N2
A/environment/Korea/CSM12/2007_H1N2
1955
1960
1965
1970
1975
1980
1985
1990
1995
2000
2005
2010
2015
2020

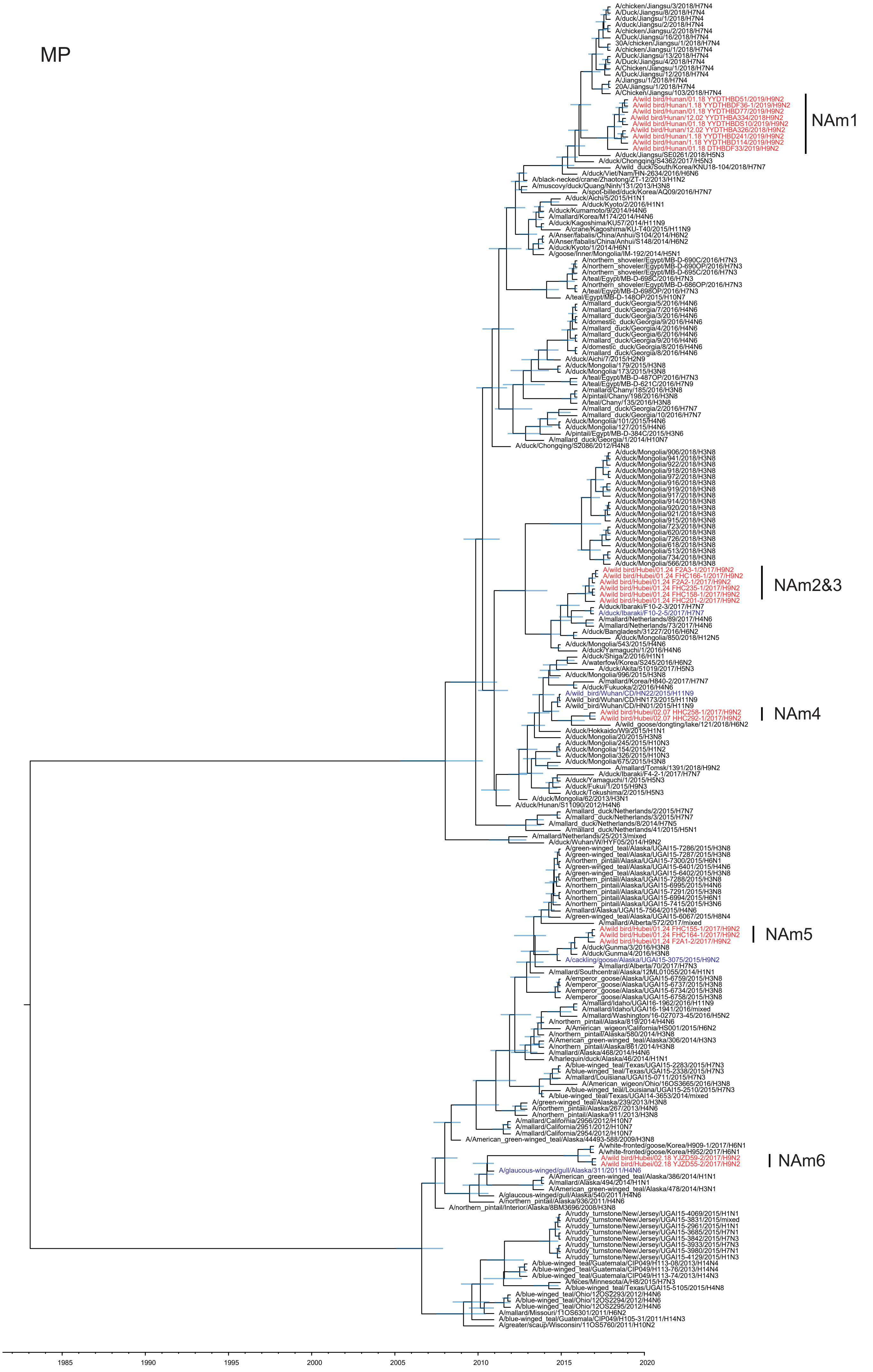

MP
NAm1
NAm2&3
NAm4
NAm5
NAm6
1985
1990
1995
2000
2005
2010
2015
2020

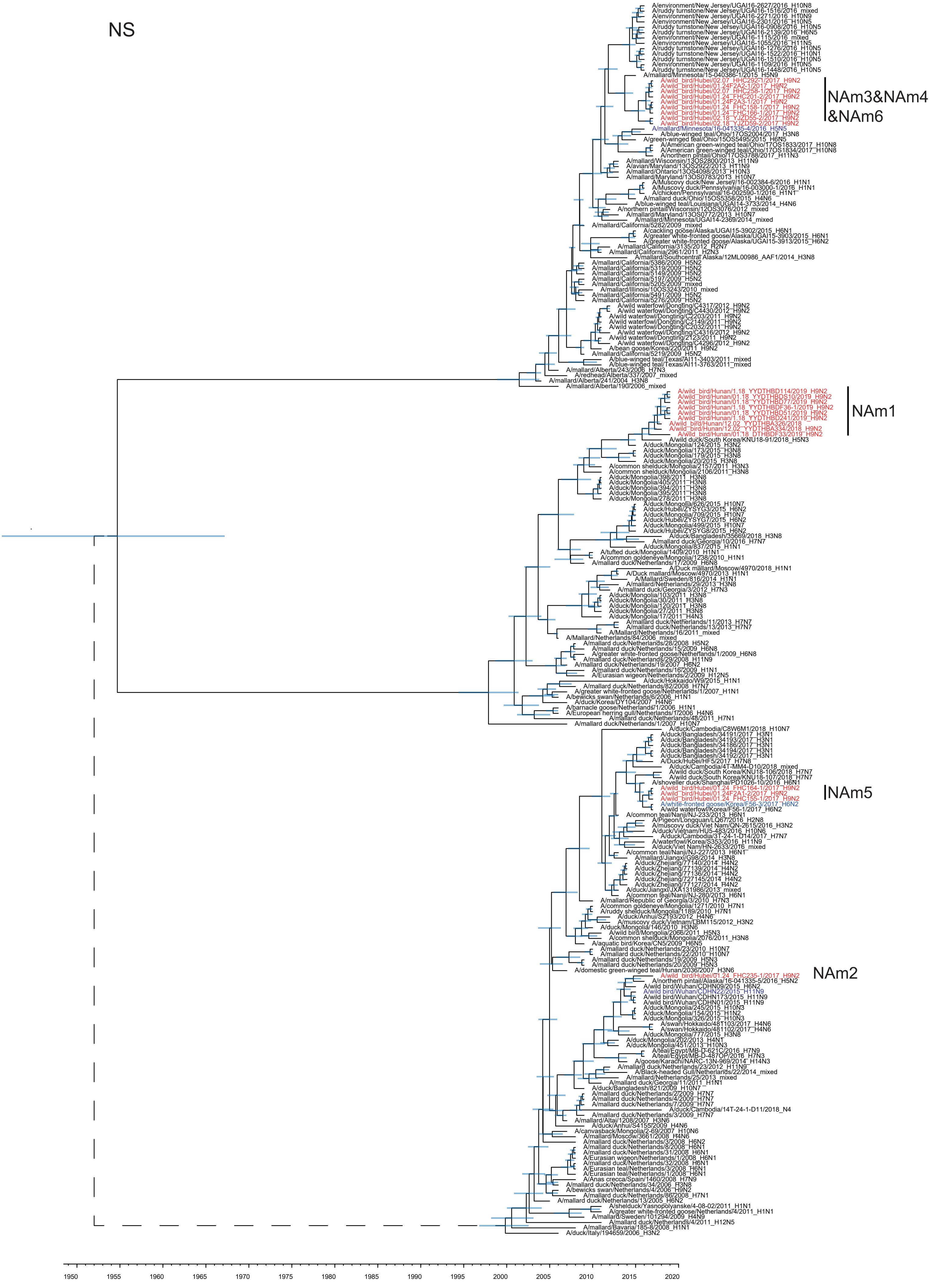
NS
NAm3&NAm4
&NAm6
NAm1
NAm5
NAm2
1950
1955
1960
1965
1970
1975
1980
1985
1990
1995
2000
2005
2010
2015
2020

Fig S4. Molecular dating analysis of 22 H9N2 viruses containing genes of North American lineage. Maximum clade credibility trees of each gene segments were established from Bayesian Evolutionary Analysis using Sampling Trees (BEAST) v1.8.3 under the SRD06 substitution model, with a strict clock and chain length of 100,000,000. Furthermore, Tracer v1.6 was used to confirm the reliability of the results. The trees were summarized by Tree Annotator with 10% burn-in cutoffs. Sequences reported in this study are marked in red. 95% highest posterior density (HPD) of age estimates of nodes are shown as horizontal bars.
